# Polymer-supported triphenylphosphine: application in organic synthesis and organometallic reactions

**DOI:** 10.1039/c9ra07094j

**Published:** 2019-11-05

**Authors:** Ziad Moussa, Zaher M. A. Judeh, Saleh A. Ahmed

**Affiliations:** Department of Chemistry, College of Science, United Arab Emirates University P.O. Box 15551 Al Ain United Arab Emirates zmoussa@uaeu.ac.ae +971-3-7134928 +971-3-7135396; School of Chemical and Biomedical Engineering, Nanyang Technological University 62 Nanyang Drive, N1.2-B1-14 Singapore 637459 Zaher@ntu.edu.sg +65-67947553 +65-67906738; Department of Chemistry, Faculty of Applied Science, Umm Al-Qura University 21955 Makkah Saudi Arabia saahmed@uqu.edu.sa saleh.a.ahmed@aun.edu.eg saleh_63@hotmail.com; Department of Chemistry, Faculty of Science, Assiut University 71516 Assiut Egypt

## Abstract

This comprehensive review highlights the diverse chemistry and applications of polymer-supported triphenylphosphine (PS-TPP) in organic synthesis since its inception. Specifically, the review describes applications of the preceding reagent in functional group interconversions, heterocycle synthesis, metal complexes and their application in synthesis, and total synthesis of natural products. Many examples are provided from the literature to show the scope and selectivity (regio, stereo, and chemo) in these transformations.

## Abstract 2

This comprehensive review highlights the diverse chemistry and applications of polymer-supported triphenylphosphine (PS-TPP) in organic synthesis since its inception.

## Graphical abstract

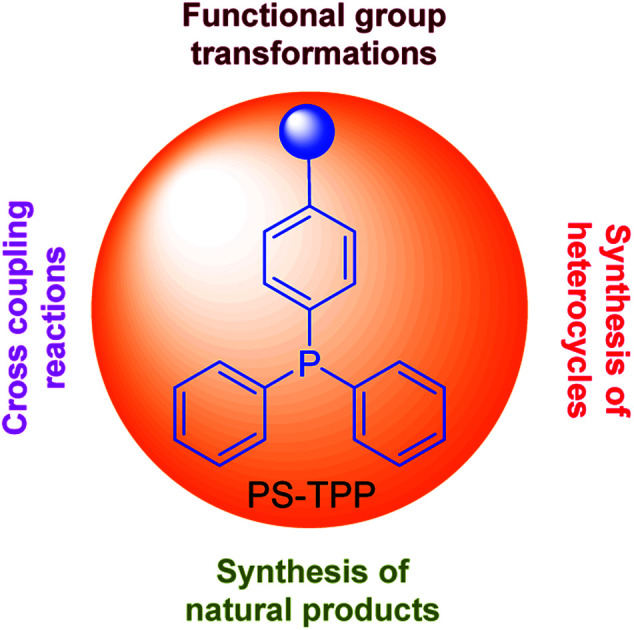

## 1. Introduction

One of the latest advances in recent years has been the utility of solid-phase synthesis as a strategy to prepare chemical libraries,^1^ biologically active molecules, and natural products.^2,3^ Adaptation of solution phase synthetic techniques to use on solid supports offers some key advantages over solution-phase chemistry such as ease of purification, recyclability of the solid matrix, and use of excess reagents to achieve complete reaction conversion. Consequently, polymer-supported reagents have found many applications in synthetic organic chemistry. As such, the use of supported reagents in medicinal chemistry has been nicely showcased by Ley in the multi-step synthesis of drug targets and natural products such as sildenafil (Viagra),^4^ epimaritidine^5^ and epibatidine.^6^ One of the most useful reagents to which many chemical transformations can be credited is polymer-supported triphenylphosphine (diphenyl-polystyrylphosphine or polystyryldiphenylphosphine (1), PS-PPh_2_), an analogue of the ubiquitous triphenylphosphine (PPh_3_). The latter is a common reagent used in many chemical transformations where in many cases it gets oxidized to triphenylphosphine oxide (Ph_3_PO). Removal of Ph_3_PO from the product requires tedious chromatographic separation and/or crystallization processes which renders PPh_3_ impractical. As an alternative, polystyrene-supported triphenylphosphine was introduced. This reagent has an advantage over the its soluble counterpart because it can be separated along with any oxidation byproduct by filtration. The reagent can be easily prepared in one step and for convenience is commercially available from several chemical suppliers (100–200 mesh, extent of labeling: ∼3 mmol g^−1^ triphenylphosphine loading). The immobilized reagent was first reported in 1971 and has been extensively used ever since.^7^ However, the full synthetic potential of the reagent and variations thereof is still being established and several research groups around the globe are still aggressively pursuing such endeavors as demonstrated by the recent surge of research articles published by various groups on a wide range of transformations mediated by this reagent. The PS-PPh_2_ reagent has not yet been thoroughly reviewed in the literature except in some instances where it was briefly featured as a part of reviews of much wider scope.^8–10^ Thus, in this comprehensive review, an attempt to give an overall detailed picture of the synthetic utility of PS-PPh_2_ has been made by taking many examples from the literature covering almost 50 years. The review focuses on the preparation of PS-PPh_2_ and its utility in functional group interconversions, transition metal complexes and their reactions, heterocyclic synthesis, and preparation of natural products. Special focus has been dedicated to the impact of the structural framework of the substrate, substitution pattern, and configuration on reaction times and conditions, as well as on the stereo-, chemo, and regiochemical outcome of the reaction. Therefore we have discussed the synthetic schemes and entries in every table for the various transformations in detail. The scope of the review will be limited to PS-PPh_2_ and its reactions.

## 2. Preparation of polystyryldiphenylphosphine (1)

**Scheme 1:**
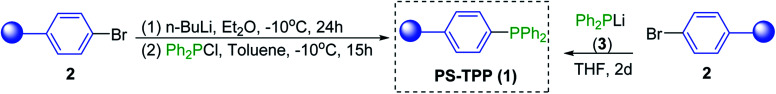
Synthesis of DVB-crosslinked polystyryldiphenylphosphine 1.

Generally, one of the simplest approaches to immobilize a phosphine ligand on a polymer support involves direct reaction of the desired ligand with a functionalised polymer such as bromopolystyrene or Merrifield's resin. Polystyrene resins are commercial available in a variety types, cross-link densities, and particle sizes. The cross-linked diphenyl-polystyrylphosphine (PS-PPh_2_, 1) can be prepared from bromopolystyrene (2) *via* a lithium–halogen exchange process by initial lithiation of the polymer support 2 to form lithiated polystyrene (Scheme 1), followed by reaction with chlorodiphenylphosphine (PPh_2_Cl).^7,11–13^ There are however drawbacks associated with this approach. Reaction of cross-linked bromopolystyrene with butyllithium often leads to unwanted side-reactions involving the C=C bonds of the divinylbenzene crosslinking. Consequently, the product resin becomes contaminated and shows poor swelling properties. More efficient preparation of the polystyryl-lithium species that precludes attack of the divinylbenzene crosslinking moiety involves reaction of cross-linked polystyrene with the 1 : 1 complex of *n*-butyl-lithium and *N*,*N*,*N*′*N*′-tetramethylethylenediamine (TMEDA).^14^ An alternative preparation of PS-PPh_2_ (1) involves reaction of cross-linked bromopolystyrene (2) with lithium diphenylphosphide (3).^7,12,13,15^ This method represents the more common route for the attachment of monodentate phosphines to polymer resins. PS-PPh_2_ is insoluble in all typical organic solvents and is readily available through several chemical vendors as 2% divinylbenzene crosslinked polystyrene beads (100–400 mesh size) with loading ranges from 1.0 mmol P per g to over 3.0 mmol P per g. The reagent can be handled like a typical solid and is amenable to long term storage, although preferably under inert atmosphere to avoid aerial oxidation.

## 3. Polystyryldiphenylphosphine-mediated functional group interconversions

### 3.1. Wittig reagents

#### 3.1.1. Carbonyl olefination with *p*-polystyryldiphenyl-benzyl-phosphonium chloride

The Wittig reaction^16^ represents on of the most used chemical methods for olefin synthesis involving phosphines in solution. Not surprisingly, the first reported application of PS-TPP involved a Wittig reaction. Castells was the first to report the use of polymer-bound triphenylphosphine in this capacity to synthesize stilbene (40–60% yield) from the reaction of benzaldehyde with *p*-polystyryldiphenyl-benzyl-phosphonium chloride, prepared by reacting PS-TPP with neat benzyl chloride.^7^ A concurrent investigation by Heitz and Michels^17^ on polymer-supported Wittig synthesis of olefins using polystyrene crosslinked with 0.5 and 2.0 wt% of divinylbenzene (DVB) found that higher yields were possible with a 0.5% crosslinked polystyrene support. This was because only about 75% of the pore volume in a swollen polymer crosslinked with 2% DVB was accessible to substrates with molar masses 300–400, according to gel-chromatographic studies. Heitz and Michels^18^ also investigated steric control of the Wittig reaction on triphenylphosphane resins. They found that the adduct formed from the reaction of the aldehyde with the ylide can be selectively converted either to the *trans*- or *cis*-olefin as the major product in the mixture. The formation of the *cis*-olefin is kinetically controlled and favored in salt-free medium while the *trans*-olefin is thermodynamically controlled and predominates if the adduct is formed at low temperature (−78 °C) as the lithium salt is generated by adding lithium perchlorate as a Lewis acid. The study was, however, very limited in scope as the diastereoselectivity was poor and only reported for three olefins. Another concurrent study on the preparation and application of polymeric phosphoranes in the Wittig reaction was reported by Mckinley and Rakshys using the standard 2% crosslinked polystyryldiphenyl phosphine.^19^ Although the carbonyl–olefination reactions proceeded with methylene, ethylene, and benzylidene polymeric ylides using aromatic and aliphatic aldehydes and ketones, the conversion of the carbonyl compound was incomplete and yields were generally low (24–72%). It is noteworthy that these earlier papers reported syntheses of alkenes in fluctuating yields, depending on the polymer used and particular reaction conditions. Thus, further improvements were required for a useful methodology of general applicability.

#### 3.1.2. Polymer-supported phase transfer catalysed Wittig reaction

**Scheme 2:**
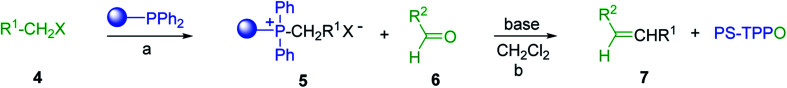
Polymer-supported phase transfer catalysed Wittig reaction. Reagents and conditions: (a) PS-TPP, chlorobenzene; (b) 5 : 6 (1.5 : 1 equiv.), 50% NaOH, TBAI or CTAB (2 mol%), 2–16 h.

Phase transfer catalysed reactions^20^ have been used extensively in organic synthesis, although they have been used to a much lesser extent in polymer chemistry. Thus, chemical reactions that combine the advantages of both experimental techniques are of interest. Hodge *et al.* described a phase transfer catalysed Wittig reaction between aldehydes 6 and polymer-bound phosphonium salt residues 5, prepared from PS-TPP and various organic halides 4 (Scheme 2).^21,22^ The reaction proceeds at room temperature and the small molecules are tethered to the polymer with bonds which are stable to both acid and base. The Wittig reaction was carried out in CH_2_Cl_2_ with 50% aqueous NaOH and a phase transfer catalyst (tetrabutylammonium iodide (TBAI) or cetyltrimethylammonium bromide (CTAB)). These conditions offered the highest reported olefin 7 yields compared to those reported by earlier work on polymer-supported Wittig reagents.^17–19,23,24^

As shown in Table 1, high yields of essentially pure alkenes were obtained from arylalkylphosphonium salts and various aldehydes (entries 1–9). However, while the alkylphosphonium salt (entry 10) reacted with a moderate yield with a reactive aldehyde, alkylphosphonium salts did not react with any aldehydes (entry 11). The Wittig reactions were performed with both, crosslinked and linear PS-TPP, although the former was more convenient since the resulting oxidized byproduct could be directly removed by filtration whilst the latter and it oxide were soluble and required precipitation workup. This renders linear polymers generally unsuitable. The isomeric ratios (*cis* : *trans*) were not determined for all olefins, although the measured ones were comparable to those obtained under conventional phase transfer catalysed conditions (entry 1; *E* : *Z* (100 : 0); entry 3; *E* : *Z* (57 : 43); entry 4; *E* : *Z* (44 : 56)). A point that merits comment is that the Wittig reaction can take place without any added catalyst, albeit more slowly, producing much lower yields as well. Ketones also failed to react with the phosphonium salts.

**Table 1:** Reactions of polymer-bound phosphonium salts 5 derived from PS-TPP (1) and aldehydes 6 under phase-transfer conditions

Entry	Halide 4 (R^1^-X)	Aldehyde 6	Catalyst	Yield 7 (%)
1	Benzyl chloride	9-Formylanthracene	CTAB	98
2	Benzyl chloride	Benzaldehyde	CTAB	92
3	Benzyl chloride	*p*-Methyl-benzaldehyde	CTAB	100
4	Benzyl chloride	*p*-Chloro-benzaldehyde	TBAI	97
5	Benzyl chloride	Furan-2-aldehyde	CTAB	90
6	Benzyl chloride	Heptaldehyde	TBAI	93
7	4-*t*-Butylbenzyl chloride	Formaldehyde	CTAB	95
8	2-Bromoethylnaphthalene	β-Naphthaldehyde	CTAB	65
9	2-Bromoethylnaphthalene	Formaldehyde	CTAB	67
10	Allyl bromide	*p*-Chloro-benzaldehyde	None	78
11	MeI or *n*-hexyl bromide	*p*-Chloro-benzaldehyde	TBAI	0

#### 3.1.3. Wittig reagents bound to crosslinked polystyrenes with variable crosslinking densities

**Scheme 3:**
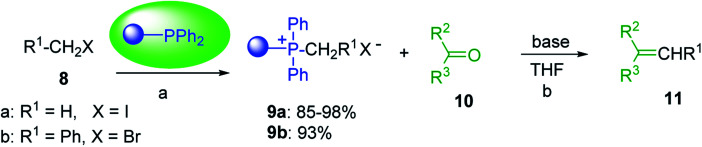
Polymer-supported Wittig reactions. Reagents and conditions: (a) PS-TPP 1 : halide 8 (1 : 2 molar ratio), DMF, 70 °C, 2 d; (b) 9 : sodium methylsulfinylmethylide (1 : 3 molar ratio), THF/DMSO (1 : 1), −10 °C, 6 h, then 10 (1 molar equiv.), RT, 6 h, then reflux 24 h.

Building on the earlier work describing polymer-supported Wittig reagents, Ford *et al.* reported a detailed study on the impact of varying the extent of crosslinking of the polystyrene support on yields of Wittig products.^13^ The polymer-supported reagents with 0.5% and 2% cross-linked polystyrenes that had been used earlier were too gelatinous and unsuitable for large-scale filtration.^17^ Thus, more highly crosslinked and rigid polystyrene supports were being sought out, although on the expense of lower Wittig yields, since penetration of reagents into all of the functional sites and out of the more highly cross-linked polymer matrices would be expected to be poor especially with large substrates. Ford described the use of Wittig reagents supported on polystyrenes with up to 20% cross-linking and with reactants as bulky as 10-nonadecanone (Table 2, entries 8 and 9) and the 3-keto steroid, cholest-4-en-3-one (Table 2, entries 10 and 11). The Wittig reagents were prepared on 2%, 8%, and 20% DVB crosslinked polystyrene from the reaction of crosslinked PS-TPP and either MeI (8a) or BnBr (8b), to afford the methyl-(9a) and benzylphosphonium (9b) salts, respectively (Scheme 3).

**Table 2:** Alkenes from polymer-bound methylphosphonium salts 8a

Entry	Copolymer, % crosslinking	Carbonyl compound 10	Wittig product 11	Yield (%)
1	2	(CH_2_)_5_C=O	(CH_2_)_5_C=CH_2_	99
2	2	PhCH=CHCHO	PhCH=CHCH=CH_2_	95
3	8	PhCH=CHCHO	PhCH=CHCH=CH_2_	52
4	20	PhCH=CHCHO	PhCH=CHCH=CH_2_	83
5	2	Ph_2_C=O	Ph_2_C=CH_2_	94
6	8	Ph_2_C=O	Ph_2_C=CH_2_	61
7	20	Ph_2_C=O	Ph_2_C=CH_2_	74
8	2	(*n*-C_9_H_19_)C=O	(*n*-C_9_H_19_)C=CH_2_	96
9	20	(*n*-C_9_H_19_)C=O	(*n*-C_9_H_19_)C=CH_2_	62
10	2	Cholest-4-en-3-one	3-Methylenecholest-4-ene	91
11	20	Cholest-4-en-3-one	3-Methylenecholest-4-ene	87

As shown in Scheme 3 and Table 2, Wittig reactions of the methylidenephosphoranes, generated from phosphonium salts 9 under basic conditions, were complete after 6 h at room temperature, followed by heating for 24 h at 60 °C. The chemical yields in all cases depended on the polymer as follows: 2% crosslinked polymer produced higher yields than >20% crosslinked polymer >8% crosslinked polymer. A similar trend was observed for olefins generated from polymer-bound benzylphosphonium salts. In this case, *E*/*Z* diastereomeric ratios ranged from 72/28 to 43/57. In general, reactions of the phosphoranes 9 with aldehydes and ketones afforded olefins in 73–96% yields with the 2% crosslinked polymer, 52–77% yields with the 8% cross-linked polymer, and 72–87% yields with the 20% cross-linked macroporous polymer. Ford showcased the utility of phosphonium salts on 2% cross-linked polystyrene and on 20% cross-linked macroporous polystyrene in the synthesis of ethyl retinoate.^25^ The preparation of olefins from polymer-supported phosphonium salts derived from PS-TPP (1) and various carbonyl compounds and elaboration thereafter has also been exploited by Ley for the synthesis of β-hydroxyamines.^26^

#### 3.1.4. Mono-olefination of symmetrical dialdehydes with polymer-supported Wittig reagents derived from PS-TPP and various halides

**Scheme 4:**
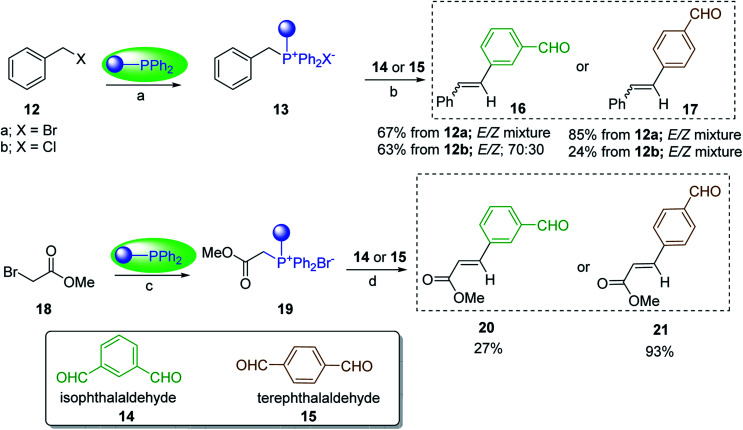
Mono-olefinization of dialdehydes. Reagents and conditions: (a) PS-TPP : BnBr (or BnCl); (1 : 6.5 (or 1 : 16.3 for BnCl) molar ratio), benzene, reflux, 16 h (with BnBr) or 5 h (with BnCl); (b) 13 : 14: (or 15); (1 : 1 molar ratio), ethylene oxide (10.5 equiv.), benzene, 68 h at RT, then reflux at 45–50 °C for 6 h; (c) PS-TPP : methyl bromoacetate (18); (1 : 3.6 molar ratio), benzene, 7 d; (d) 18 : 14 or 15; (1 : 1 molar ratio), ethylene oxide (14.3 equiv.), benzene.

Castells and co-workers exploited the pseudo high dilution environment provided by polymer supported phosphonium salts to effect mono-olefination of symmetrical dialdehydes (Scheme 4).^27^ The olefination reactions were carried out using 1 : 1 molar ratio of polymer supported phosphonium salts 13 and 19 to dialdehydes (isophthalaldehyde (14) and terephthalaldehyde (15)). The phosphonium salts were prepared from PS-TPP 1 and benzyl bromide (12a), benzyl chloride (12b), or methyl bromoacetate (18). The Wittig reaction afforded olefins in variable yields as *E*/*Z* geometric isomers (16 and 17), and in the case of phosphonium salt 19 as single diastereomers (20 and 21). The exclusive mono-olefinization was attributed to the formation of a betaine intermediate with one of the two aldehydes which blocks all active sites on the polymer, preventing the second carbonyl group from reacting. Interestingly, mono-olefinization still took place even when excess phosphonium salt to dialdehyde (2 : 1 molar ratio) was employed.

#### 3.1.5. Preparation of vinyl ethers and thioethers *via* Wittig reagents

**Scheme 5:**
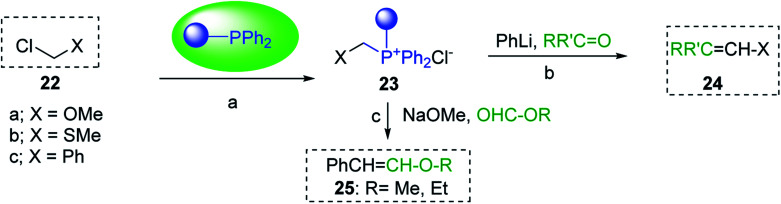
Preparation of vinyl ethers, thioethers, and alky-β-styrylethers. Reagents and conditions: (a) 23a; PS-TPP : MeOCH_2_Cl : carbonyl (1 : 1.5 : 1), toluene, 50 °C, 48 h; 23b; PS-TPP : MeSeCH_2_Cl : carbonyl (1 : 1.5 : 1), toluene, 50 °C, 48 h; 23c; PS-TPP : BnCl (1 : 2), toluene, 50 °C, 24 h; (b) 24a : PhLi (1 : 1), ether, RT, 24 h; 24b : PhLi (1 : 1), THF, RT, 24 h; (c) 25: PS-TPP : 23c (1 : 1), NaOMe, MeOH, RT.

Polystyryldiphenyl-methoxymethyl- (23a), methylthiomethyl-phosphonium (23b), and benzyl-phosphonium chlorides (23c) were prepared by Akelah^23^ by treating PS-TPP (1) with chloromethylmethylether (22a), chloromethylthioether (22b), and benzyl chloride (22c) respectively (Scheme 5). These reagents were employed for the conversion of carbonyl compounds and formate esters into vinyl-ethers (24a), thioethers (24b), and alky-β-styrylethers (25) in high yields.

### 3.2. PS-TPP halophosphorane complexes

#### 3.2.1. Polymer-supported dichlorophosphorane

**Scheme 6:**
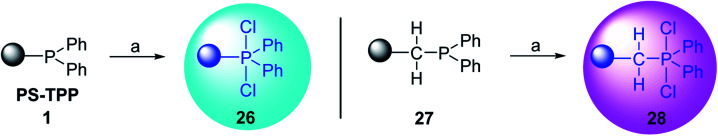
Preparation of halophosphorane complexes. Reagents and conditions: (a) (i) 40% peracetic acid in acetic acid, CH_2_Cl_2_, 25 °C, 3 h; (ii) phosgene (excess), 25 °C, 1 h.

Polymer-supported dichlorophosphoranes (26 and 28) were first prepared in 1974 from PS-TPP (1) and its related benzyl analogue 27 by initial oxidation with peracetic acid to the corresponding polymer-bound phosphine oxides, followed by treatment with carbon oxychloride (phosgene) to provide the desired halophosphorane complexes 26, and 28 (Scheme 6).^28^

Reagents 26 and 28 were used successfully for the synthesis of acid chlorides from carboxylic acids (Table 3, entries 1–3 and 5–7), a nitrile from a primary amide (entry 4), a chloroalkene from a ketone (entry 10), an imidoyl chloride from an anilide (entry 8), and an alkyl chloride from an alcohol (entry 9). The recovered resin-bound phosphine oxide was rephosgenated back to the halophosphorane reagent for reuse.

**Table 3:** Chemical conversions using cross-linked polymeric phosphine dichlorides^a^

Entry	Polymeric phosphine dichloride	Reactant	Product	Yield (%)
1	26	PhCH_2_CO_2_H	PhCH_2_COCl	100
2	26	*p*-CH_3_C_6_H_4_CO_2_H	*p*-CH_3_C_6_H_4_COCl	98
3	26	HO_2_CC_6_H_4_CO_2_H (*p*)	ClOCC_6_H_4_COCl (*p*)	91
4	26	PhCH_2_CONH_2_	PhCH_2_CN	78
5	28	HO_2_CC_6_H_4_CO_2_H (*p*)	ClOCC_6_H_4_COCl (*p*)	95
6	28	HO_2_CC_6_H_4_CO_2_H (*p*)	ClOCC_6_H_4_COCl (*p*)	98
7	28	HO_2_CC_6_H_4_CO_2_H (*m*)	ClOCC_6_H_4_COCl (*m*)	87
8	28	PhCH_2_CONHC_6_H_5_	PhCH_2_C(Cl)=NC_6_H_5_	93
9	28	PhCH_2_OH	PhCH_2_Cl	88
10	28	PhCH_2_COCH_3_	PhCH_2_C(Cl)=CH_2_	75

a Reagents and conditions: 1 : 1 molar ratios of polymeric phosphine dichloride : reactant, CH_2_Cl_2_, RT, 30 min.

#### 3.2.2. Condensation of *N*-alkoxycarbonyl α-amino acids with primary amines using PS-TPP and CCl_4_

The combination of PS-TPP and carbon tetrachloride comprises a convenient coupling system for the amidation of *N*-alkoxycarbonyl α-amino acids and primary amines.^29^ Such a process is often beset with shortcomings associated with epimerization of the *N*-terminal residue and purification of the product. However, the preceding method reported by Landi and Brinkman is not afflicted by the aforementioned problems. The reagent system proved effective for *N*-Boc (*t*-butoxycarbonyl) and *N*-Fmoc (9-fluorenylmethoxycarbonyl) amino acids, as well as *N*-CBz (benzyloxycarbonyl) substrates (Table 4). Typically, the procedure involved refluxing an equimolar mixture of the *N*-protected amino acid and the amine with 4-methylmorpholine (29) (1.1 equiv. or 2.2 equiv. if the amine was an acid salt) and PS-TPP (2 equiv.) in a binary solvent mixture of CH_2_Cl_2_ and CCl_4_ (2 : 1). Interestingly, although earlier work of Relles^28^ and Hodge^30^ described the formation of acid chlorides from the reaction of polymer-supported dichlorotriphenylphosphorane with carboxylic acids, these intermediates were not present or involved in the current condensation. The active electrophile was found to be a resin-bound mixed phosphinic anhydride.

**Table 4:** Condensation of *N*-alkoxycarbonyl α-amino acids and primary amines

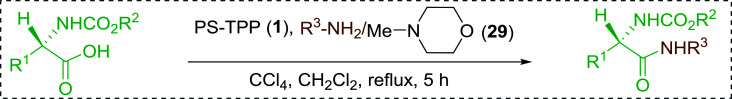				
Entry	R^1^	R^2^	Amine	Yield (%)
1	CH_2_CO_2_Bn	Bn	l-Phenylalanine-OMe·HCl	96
2	CH_2_C_6_H_4_(OBu-*t*)-4	CH_2_C_13_H_9_	l-(−)-α-Methylbenzylamine	89
3	CH_2_OBn	*t*-Bu	l-(−)-α-Methylbenzylamine	99
4	CH_2_CO_2_Bn	Bn	d-Phenylalanine-OMe·HCl	100
5	Bn	*t*-Bu	l-(−)-α-Methylbenzylamine	88
6	CH_2_CO_2_Bn	*t*-Bu	l-(−)-α-Methylbenzylamine	96
7	CH_2_CO_2_Bu-t	*t*-Bu	l-Phenylalanine-OMe·HCl	100
8	(CH_2_)_2_SMe	*t*-Bu	l-(−)-α-Methylbenzylamine	79
9	(CH_2_)_4_NHCO_2_CH_2_C_6_H_4_Cl-2	*t*-Bu	l-(−)-α-Methylbenzylamine	78

#### 3.2.3. Preparation of dipeptides using PS-TPP and CCl_4_

Polymer-supported triphenylphosphine and carbon tetrachloride have also been successfully used for the synthesis of dipeptides. Appel described the synthesis of three small peptides from *N*-protected amino acid and amino acid ester salts using PS-TPP and CCl_4_. The reaction was heated at 40 °C in acetonitrile as solvent to afford the peptides in 70–76% yields (Table 5).^31^

**Table 5:** Preparation of dipeptides with polymeric triphenylphosphine and CCl_4_^a^

Entry	N-Protected amino acid	Amino acid ester	Peptide	Yield (%)
1	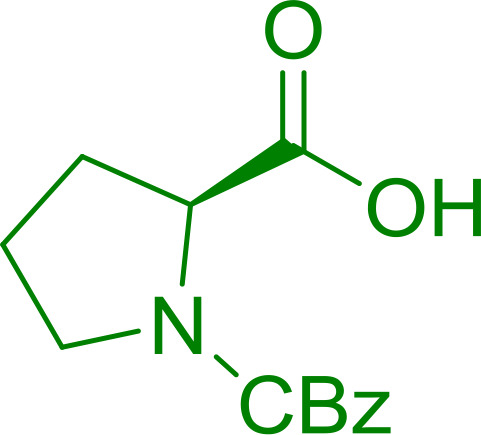	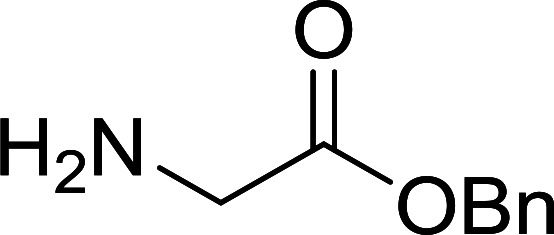	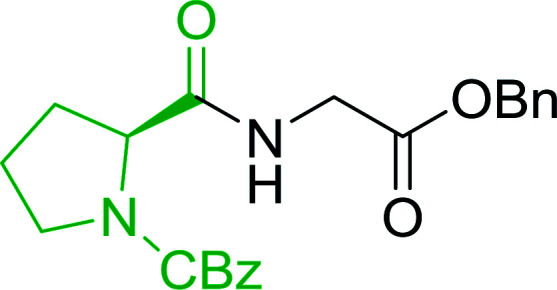	76
2	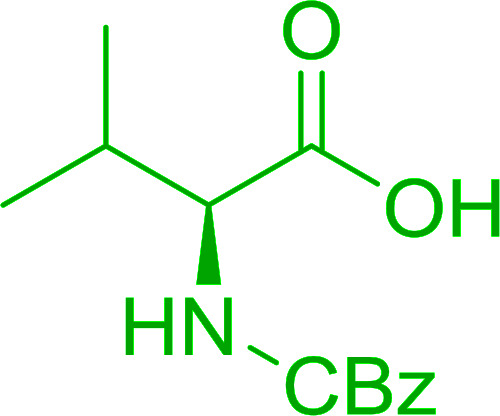	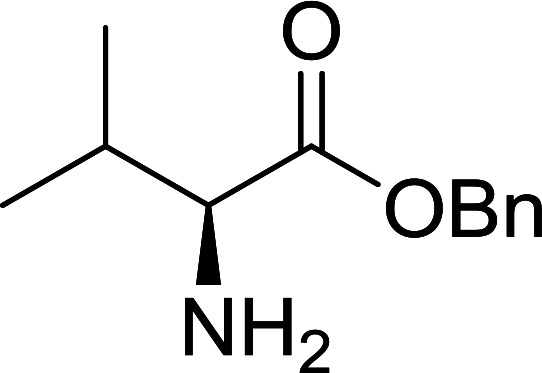	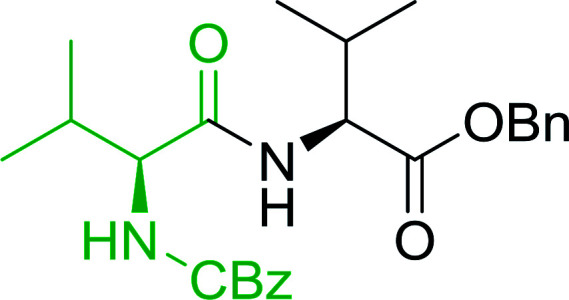	70
3	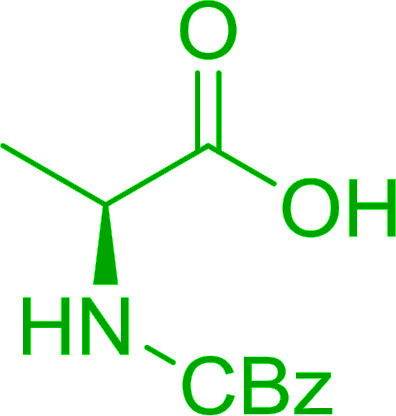	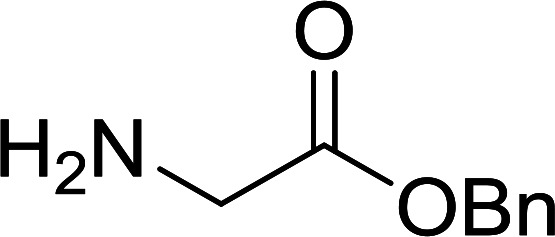	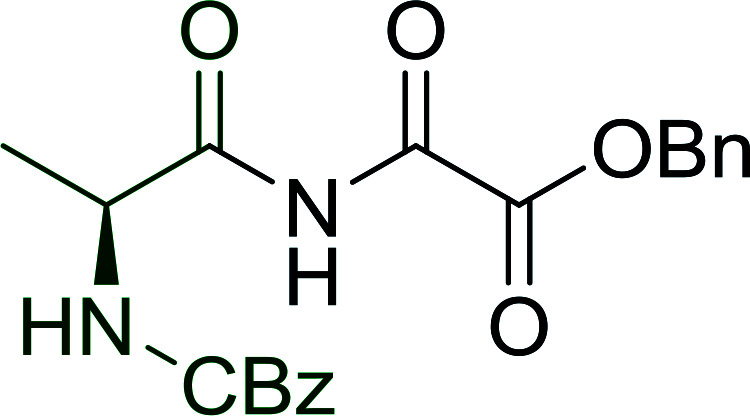	70

a Reagents and conditions: 3 : 1 : 1 : 2 molar ratios of PS-TPP/CCl_4_ : *N*-protected amino acid : amino acid ester : Et_3_N, acetonitrile, 40 °C, 1 d.

#### 3.2.4. Preparation of acid chlorides and alkyl chlorides using PS-TPP and CCl_4_

**Scheme 7:**
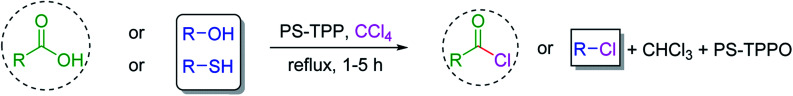
Reactions of carboxylic acids, thiols, and alcohols with PS-TPP in CCl_4_.

The PS-TPP–CCl_4_ reagent system has also been applied successfully to convert carboxylic acids into acid chlorides,^30,32^ and thiols^32^ as well as alcohols^15,30,32^ into alkyl chlorides (Scheme 7). The reactions do not generate HCl and thus the conditions are essentially neutral.

Some representative examples of the conversion of carboxylic acids into acid chlorides, and alcohols/thiols into alkyl chlorides are summarized in Table 6. Generally, a carbon tetrachloride solution of the carboxylic acid, alcohol, or thiols in the presence of excess PS-TPP (1.3–2 molar equiv.) was heated at reflux for 1–5 h and the oxidized polymer resin was removed at the end of reaction period by filtration. Carbon tetrachloride was used as a co-reagent and as a solvent. A range of aliphatic and aromatic carboxylic acids, thiols, and alcohols were suitable substrates and gave good yields (50–98%), except for the secondary alcohol, cyclohexanol (entry 13). In this case, some elimination occurred, giving a mixture of chlorocyclohexane and cyclohexene. The acetonide protecting group (entry 12) remained intact, underscoring the advantage of neutral conditions. Interestingly, substrate selectivity based on size was not a factor in determining the reaction rate considering that substrates need to diffuse into the polymer to react. For instance, 5β-cholan-24-ol (entry 14), which represents a relatively large substrate, reacted satisfactorily with the reagent. Mechanistically, the polymer-supported reactions involving the conversion of alcohols into alkyl chlorides seem to follow similar pathways to those observed for the free Ph_3_P reagent as judged by the quantity of phosphine (2 equiv.) consumed per mole of alkyl chloride and by the quantity of chloroform produced at the end of the reaction. Formation of the polymeric chlorinating species appears to be the slow step, although the PS-TPP reactions have been shown to be faster than those using Ph_3_P due to a microenvironmental effect.^32,33^ The suggested mechanism also required that polymer supported phosphine residues react together.^33^ This is consistent with Sherrington's work in which the conversion of alcohols into alkyl chlorides was retarded or even inhibited when highly crosslinked PS-TPP (>15% crosslink ratio of styrene to DVB) was utilized.^34^ Although highly crosslinked polymers favour the formation of chloroalkanes because the alcohol penetrates the matrix more easily, the inherent rigidity and lack of backbone mobility hinders the approach and reaction of non-adjacent phosphine residues, and therefore inhibit the preceding transformation. As a result, the aforementioned chlorination reactions are best carried with Ph_3_P supported on polystyrene of low crosslink ratio (1–2%).^35^

**Table 6:** Conversion of carboxylic acids into acid chlorides and alcohols into alkyl chlorides^a^

Entry	Carboxylic acid/alcohol	Acid chloride/alkyl chloride	Yield (%)
1	*n*-Octanoic	Octanoyl chloride	63
2	Phenoxyacetic	Phenoxyacetyl chloride	50
3	Stearic	Stearoyl chloride	82
4	Cinnamic	Cinnamoyl chloride	77
5	Benzoic	Benzoyl chloride	90
6	α-Furoic	2-Furanoyl chloride	82
7	β-Naphthoic	2-Naphthoyl chloride	84
8	*n*-Octanol	1-Chlorooctane	98
9	2-*n*-Butoxyethanol	1-(2-Chloroethoxy)butane	94
10	Benzyl alcohol	Benzyl chloride	88
11	Hexadecyl alcohol	1-Chlorohexadecane	98
12	4-Hydroxymethyl-2,2-dimethyl-1,3-dioxolane	4-(Chloromethyl)-2,2-dimethyl-1,3-dioxolane	78
13	Cyclohexanol	Chlorocyclohexane	40
14	5β-Cholan-24-ol	24-Chloro-5β-cholane	78
15	Octadecanethiol	1-Chlorooctadecane	78
16	3-Phenylpropanethiol	(3-Chloropropyl)benzene	82
17	Phenylmethanethiol	Benzyl chloride	95

a Reagents and conditions: 1.3 : 2 molar ratios of PS-TPP : carboxylic acid or alcohol, CCl_4_, 77 °C, 1–5 h.

#### 3.2.5. Synthesis of amides from carboxylic acids and amines using PS-TPP and CCl_4_

Hodge *et al.* also demonstrated that carboxylic acids could be directly converted into amides by treatment with carbon tetrachloride and PS-TPP in the presence of at least 2 molar equivalents of the amine (Table 7).^32^ The reaction was reminiscent to that reported earlier by Appel and Willms whereby several peptides were prepared using the same reagent.^31^ Amides were prepared in 1,2-dichloroethane (DCE) containing 10% CCl_4_ by volume (entries 1, 2 and 5) or in CCl_4_ under reflux for 3 h to afford the amides in 57–94% yield. The same system was used by Hodge to convert primary carboxamides and oximes into nitriles or imidoyl chlorides in good yields.

**Table 7:** Preparation of amides from carboxylic acids and amines^a^

Entry	Acid	Amine	Amide	Yield (%)
1	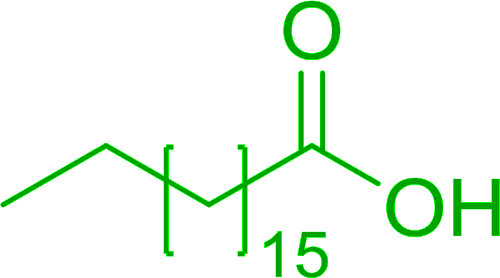	Ph–NH_2_	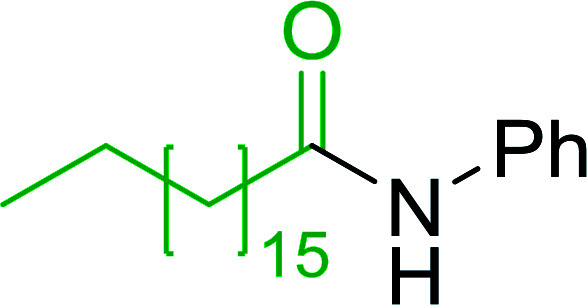	86
2	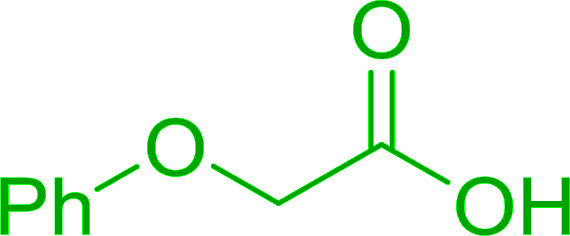	Ph–NH_2_	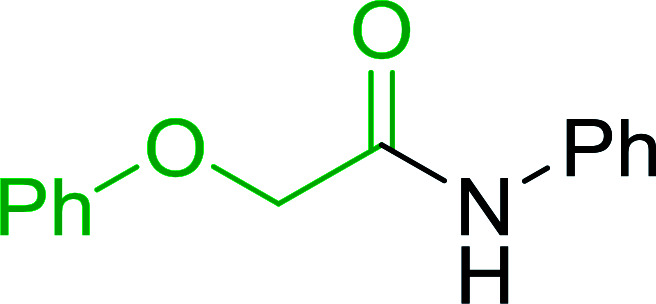	57
3	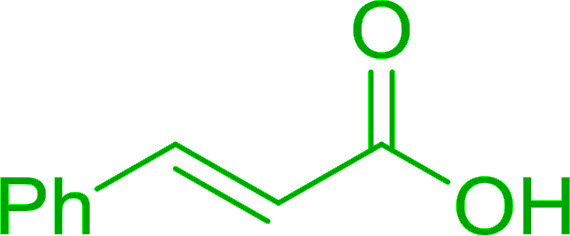	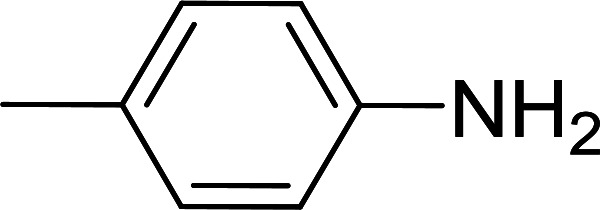	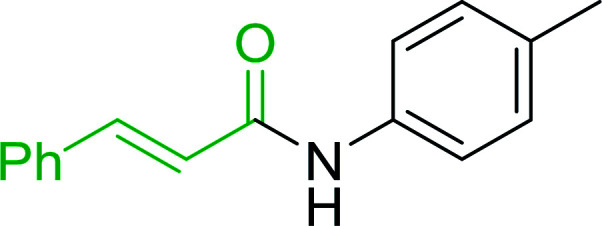	72
4	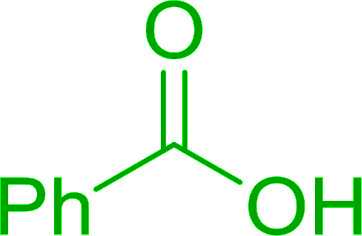	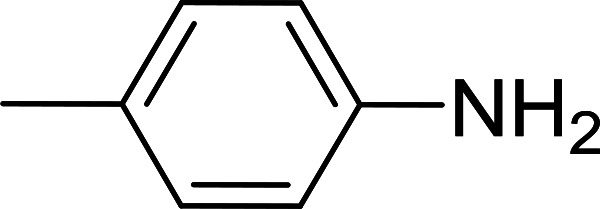	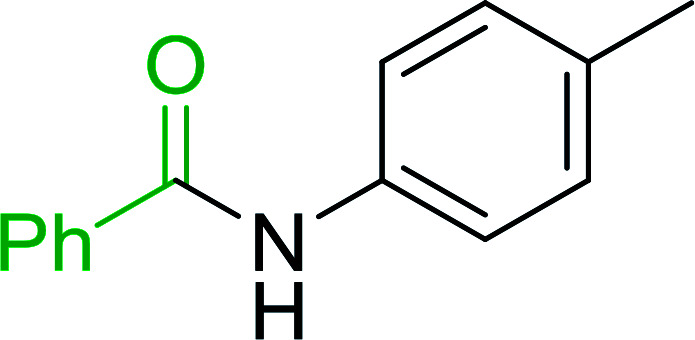	83
5	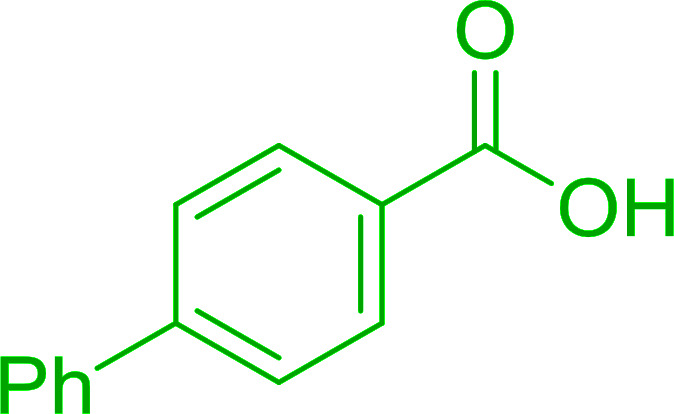	Ph–NH_2_	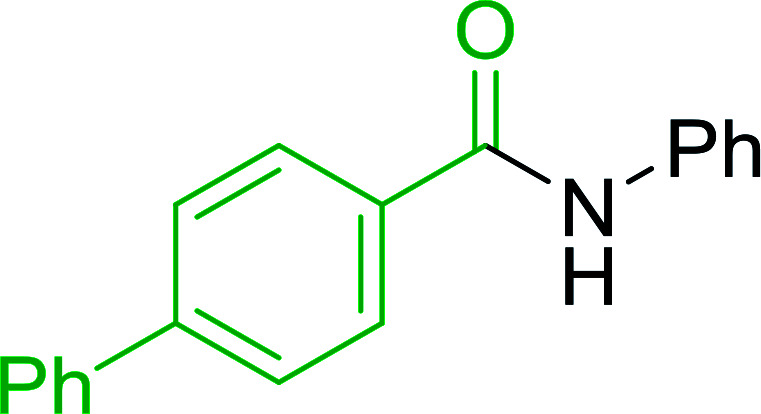	94

a Reagents and conditions: 2.2 : 1 : 2 molar ratios of PS-TPP : carboxylic acid : amine, DCE/10% CCl_4_ or CCl_4_, reflux, 3 h.

#### 3.2.6. Polymer-supported triphenylphosphine-carbon tetrabromide and triphenylphosphine dibromide: conversion of alcohols into alkyl bromides

Hodge *et al.* reported the use of polymer-supported triphenylphosphine dibromide (PS-TPP–Br_2_) and the combination of polymer-supported triphenylphosphine and carbon tetrabromide (30) (PS-TPP–CBr_4_) to convert alcohols into alkyl bromides (Table 8).^36^ The former reagent had been used earlier for the cleavage of ethers,^37^ and to prepare imidoyl bromides, ketenimines, and carbodiimides.^38^ The required PS-TPP–Br_2_ was prepared from PS-TPP and bromine in CHCl_3_ and was used to convert a range of alcohols to bromides at reflux temperature. However, the use of PS-TPP–CBr_4_ was more advantageous since HBr is not a byproduct and reactions were faster (5–40 min), cleaner, and did not require heating in the case of primary and some secondary alcohols (Table 8, entries 1–8). Bulky substrates (entries 9–11) required more vigorous conditions but nevertheless still gave good yields of the bromides. Inversion of configuration was observed with trinorbornan-*endo*-2-ol (entry 10) and (−)-bornan-2-ol (entry 11).

**Table 8:** Conversion of alcohols to bromides with PS-TPP and CBr_4_^a^

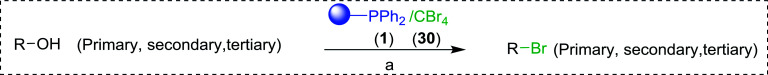			
Entry	Alcohol	Bromide product	Yield (%)
1	1-Octanol	1-Bromooctane	98
2	Benzyl alcohol	Benzyl bromide	80
3	3-Phenylpropan-1-o1	(3-Bromopropyl)benzene	81
4	4-Phenylbutan-1-o1	(4-Bromobutyl)benzene	82
5	4,4,4-Triphenylbutan-1-o1	(4-Bromobutane-1,1,1-triyl)tribenzene	73
6	Undec-10-en-1-o1	11-Bromoundec-1-ene	89
7	Cinnamyl alcohol	Cinnamyl bromide	89
8	Octan-2-o1	2-Bromooctane	76
9	Adamantanol	1-Bromoadamantane	97
10	Trinorbornan-*endo*-2-ol	Trinorbornan-*exo*-2-yl bromide	89
11	(−)-Bornan-2-ol	Bornan-*exo*-2-yl bromide	71

a Reagents and conditions: 2.2 : 1.1 : 1 molar ratios of PS-TPP : CBr_4_ : alcohol, CHCl_3_; entries 1–8 (RT, 5–40 min); entries 9–11 (61 °C, 16 h).

#### 3.2.7. Synthesis of dibromoalkenes by reaction of carbonyl compounds with PS-TPP and CBr_4_

**Scheme 8:**
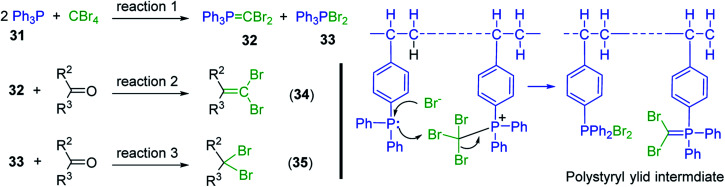
Synthesis of dibromoalkenes and *gem*-dibromides from carbonyl compounds with PS-TPP/CBr_4_ reagent system.

Aldehydes and ketones react with dibromomethylene ylides 32, generated from the reaction of carbon tetrabromide with Ph_3_P (Scheme 8, reaction 1), to give 1,1-dibromoalkenes 34 (reaction 2). Additionally, the carbonyl group may react with the accompanying triphenylphosphine dibromide (33) produced in the first step (reaction 1) to produce *gem*-dibromides 35 (reaction 3). Hodge investigated the synthesis of 1,1-dihaloalkenes by reacting carbonyl compounds with PS-TPP and carbon tetrahalides (CBr_4_ and CCl_4_).^39^ The reactions with carbon tetrachloride required harsh conditions and generally produced inseparable mixtures of dichloroalkene and *gem*-dichloride. However, the reaction with CBr_4_ proceeded at 20 °C and in most cases afforded dibromoalkenes (34) in good and consistent yields (87–98%) with minimal formation of *gem*-dibromides 35 (3–13%). Mechanistically, the olefination reactions using the PS-TPP/CBr_4_ reagent system proceeds through the polystyryl ylide intermediate shown in Scheme 8.

#### 3.2.8. Conversion of epoxides to halohydrins

Polystyryldiphenylphosphine–halogen complexes (PS-TPP–X_2_) comprise convenient reagents for the conversion of epoxides to halohydrins under very mild and non-acidic conditions.^40^ They are semi-crystalline solids which are easy to handle, stable at room temperature, and amenable to storage for weeks. However, they undergo rapid decomposition if they are exposed to moisture giving the corresponding hydrogen halide and phosphine oxide. The halogen complexes were prepared by treating PS-TPP with an equimolar amount of bromine (1 M) or iodine (1 M) solution, or by bubbling gently gaseous chlorine through the suspended polymer resin in CH_2_Cl_2_. The halogens got consumed immediately and the formation of the complexes was almost instantaneous. Epoxide-ring opening reactions proceeded in high yields (Table 9) and maintained regio- and stereo-selectivity when compared to the same transformation carried out with Ph_3_P–X_2_ complexes.

**Table 9:** Conversion of epoxides to halohydrins using PS-TPP–X_2_ complexes^a^

Entry	Epoxide	Halohydrin	Yield (%)
1	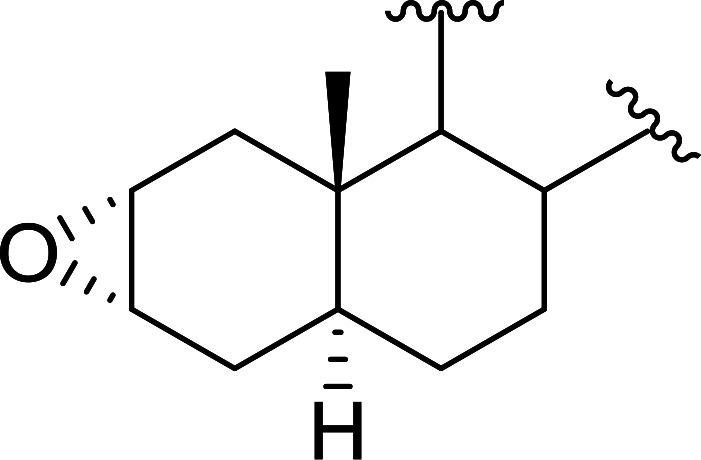	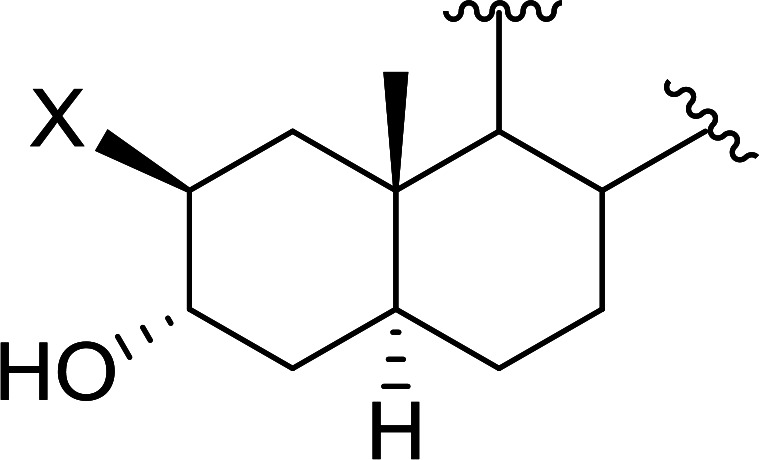	X = I: 97
			X = Br: 95
			X = Cl: 96
2	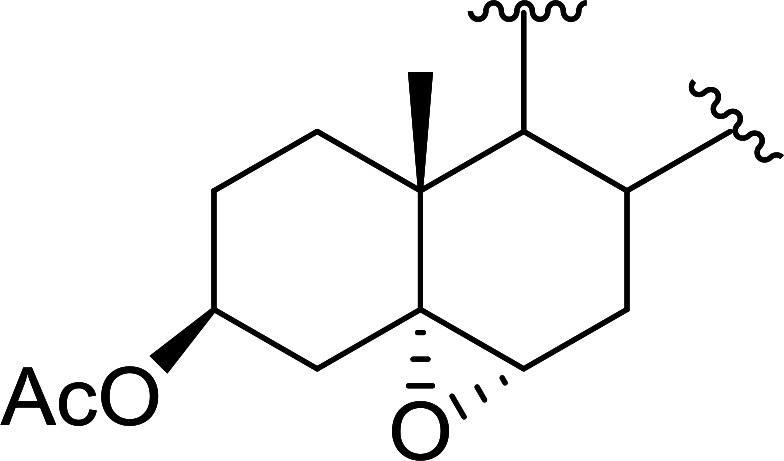	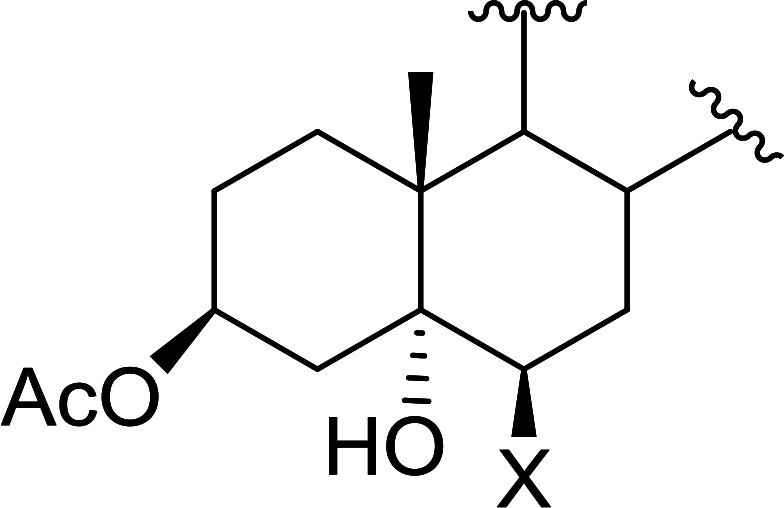	X = I: 95
			X = Br: 96
			X = Cl: 98
3	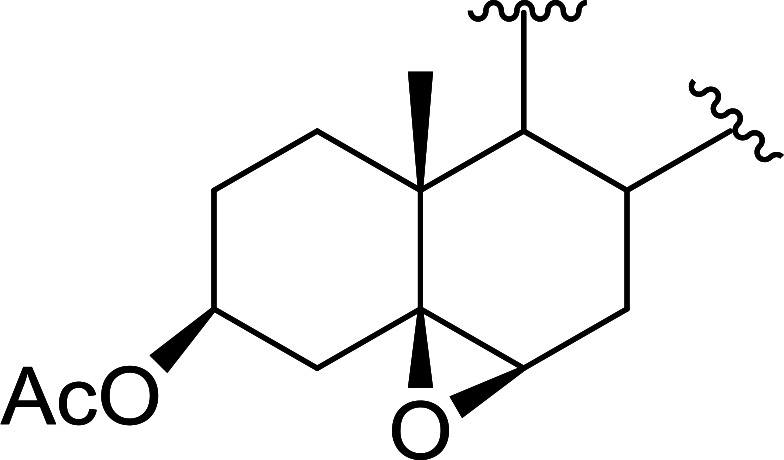	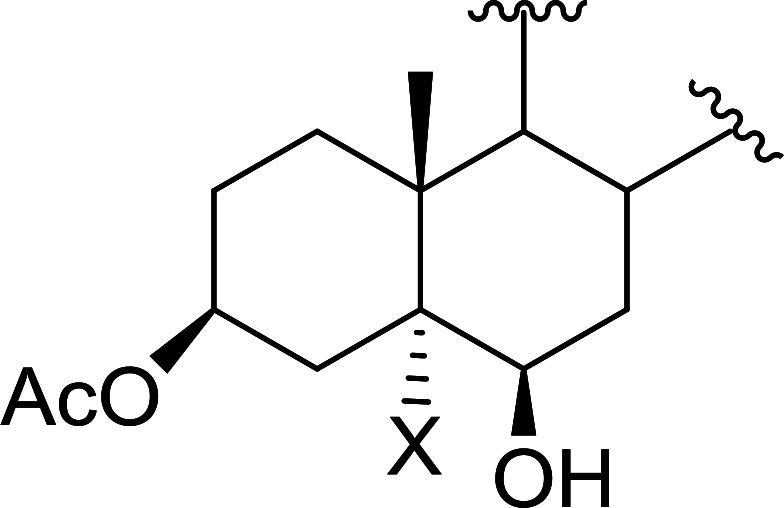	X = I: 95
			X = Br: 96
			X = Cl: 97
4	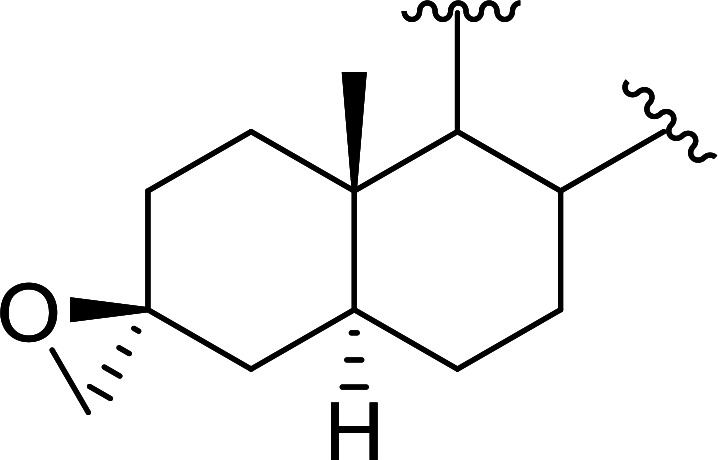	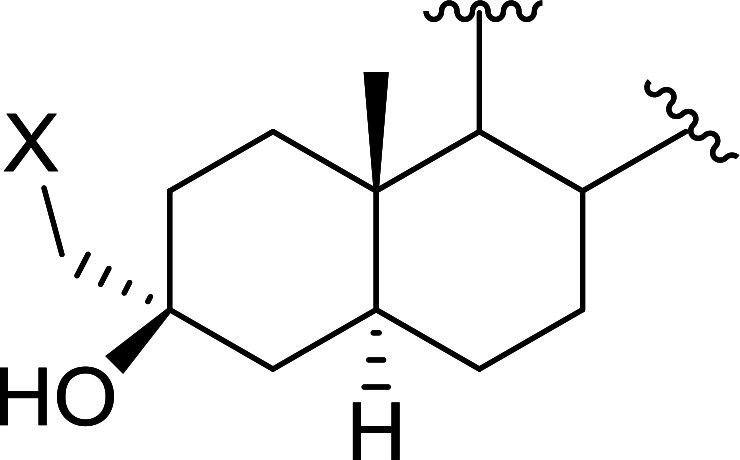	X = I: 95
			X = Cl: 97
5	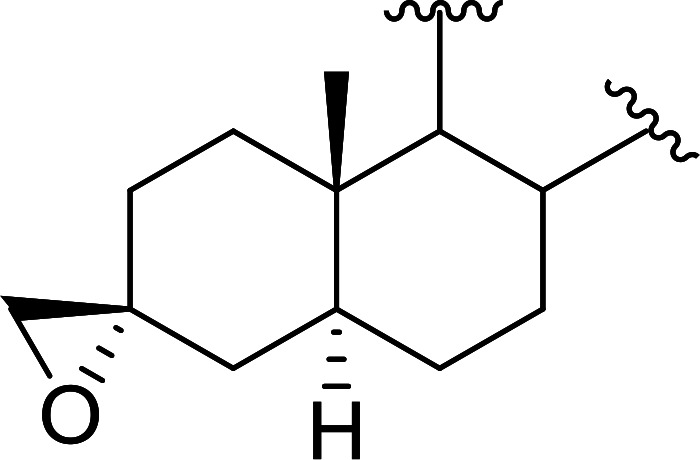	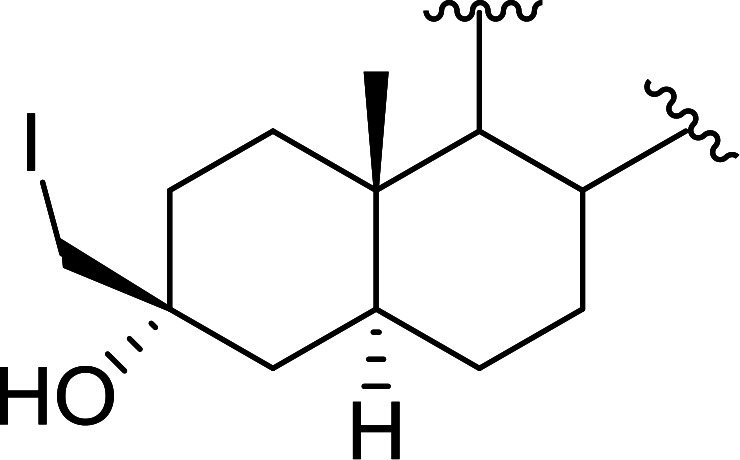	95

a Reagents and conditions: 1.2 : 1 molar ratio of PS-TPP–X_2_ : epoxide, CH_2_Cl_2_, RT, 10 min.

#### 3.2.9. Esterification of carboxylic acids using polystyryldiphenylphosphine–halogen complexes (PS-TPP–X_2_)

Caputo *et al.* also demonstrated the utility of polystyryldiphenylphosphine–halogen complexes (PS-TPP–X_2_) in the condensation of carboxylic acids and alcohols to afford esters in high yields and under mild conditions (Table 10).^41^ The complexes were prepared as before^40^ and the procedure involved adding the acid to an equimolar amount of the complex, followed by stirring at room temperature for 10–15 min. Subsequent addition of the alcohol and warming up to 40–50 °C gave the desired ester in high yield within 1–3 h. The halogen complexes act both as condensating agents and acid catalysts. The reaction was assumed to involve protonated acyl halides as the key intermediates that react with the added alcohol. The advantage of this protocol include rapid ester formation, directly from the acid, broad range of alcohol and carboxylic acid substrates, and simple workup. The iodine complex (PS-TPP–I_2_) was most practical, among the three halogen complexes, as the formation of esters with PS-TPP–Cl_2_ was slow, whereas the use of PS-TPP–Br_2_ resulted in some bromination of double bonds for unsaturated substrates.

**Table 10:** Esterification of carboxylic acids using PS-TPP–I_2_ complexes^a^

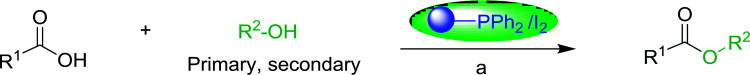			
Entry	Carboxylic acid	Alcohol	Yield (%) of ester
1	CH_3_CO_2_H	5α-Cholestan-3β-ol	96
2	CH_3_CO_2_H	5α-Cholestan-3α-ol	85
3	PhCO_2_H	CH_3_OH	90
4	PhCO_2_H	5α-Cholestan-3β-ol	85
5	CH_3_(CH_2_)_6_CO_2_H	5α-Cholestan-3β-ol	93
6	CH_3_(CH_2_)_14_CO_2_H	CH_3_OH	98
7	CH_3_(CH_2_)_14_CO_2_H	5α-Cholestan-3β-ol	92
8	CH_3_(CH_2_)_16_CO_2_H	CH_3_CH_2_OH	95
9	CH_3_(CH_2_)_16_CO_2_H	5α-Cholestan-3β-ol	94
10	Linoleic acid	CH_3_OH	87
11	Linoleic acid	5α-Cholestan-3β-ol	84

a Reagents and conditions: 1.1 : 1.1 : 1 molar ratio of PS-TPP–I_2_ : carboxylic acid : alcohol, CH_2_Cl_2_, 40–50 °C, 1–3 h.

#### 3.2.10. Microwave assisted conversion of alcohols to alkyl halides with PS-TPP–X_2_ complexes

Microwave assisted organic synthesis has been implemented as a technology in organic chemistry since the mid-1980's.^42^ Recently, a microwave assisted procedure for the conversion of allylic, benzylic and aliphatic alcohols to the corresponding alkyl bromides and iodides using polymer-supported triphenylphosphine and iodine or bromine was described by Rokhum *et al.*^43^ The iodination and bromination reactions were complete within 7 minutes under microwave irradiation conditions and gave alkyl halides in yields ranging from 76–96% (Table 11). Primary alcohols converted to the corresponding halides at a faster rate than secondary alcohols and preference of secondary over tertiary substitution was observed with unsymmetrical diols (entry 13). In case of symmetrical diols (entry 14), the mono-iodinated products were obtained in very high yields (88–93%). The methodology features high chemo- and regio-selectivity behaviour, short reaction times, product isolation requiring only filtration and solvent removal.

**Table 11:** Synthesis of alkyl halides from alcohols^a^

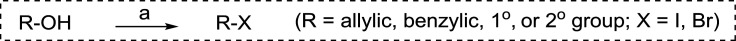			
Entry	Alcohol	Product	Yield (%)
1	Benzyl alcohol	Benzyl iodide	93
2	4-Methoxy-benzyl alcohol	4-Methoxy-benzyl iodide	96
3	3,4-Dimethoxy-benzyl alcohol	3,4-Dimethoxy-benzyl iodide	89
4	4-Nitro-benzyl alcohol	4-Nitro-benzyl iodide	96
5	3-Chloro-benzyl alcohol	3-Chloro-benzyl iodide	95
6	4-Chloro-benzyl alcohol	4-Chloro-benzyl bromide	91
7	4-Bromo-benzyl alcohol	4-Bromo-benzyl bromide	92
8	3-Phenylpropan-1-ol	(3-Bromopropyl)benzene	81
9	Octan-1-ol	1-Iodooctane	92
10	3-Methylbut-2-en-1-ol	1-Iodo-3-methylbut-2-ene	87
11	Cyclohexanol	Iodocyclohexane	76
12	Diphenylmethanol	(Iodomethylene)dibenzene	85
13	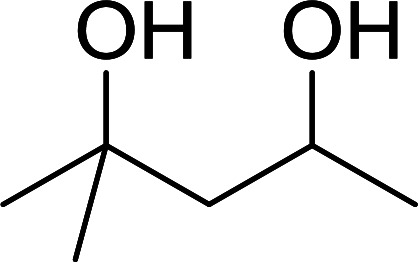	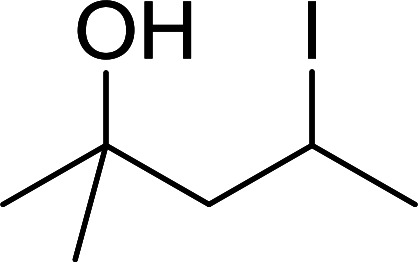	79
14	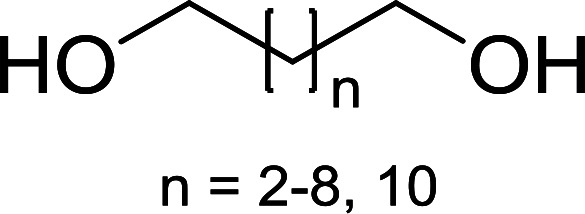	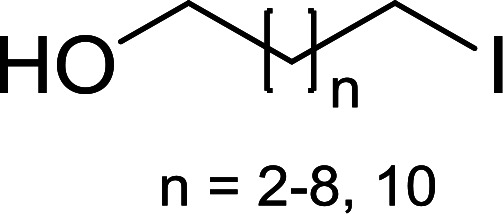	88–93

a Reagents and conditions: 1 : 1.2 : 1 molar ratio of alcohol : PS-TPP : X_2_ (X= I, Br), MW, 120 °C, CH_3_CN, 3–7 min.

#### 3.2.11. Formylation of primary and secondary alcohols using PS-TPP and iodine

The conversion of alcohols into formic acid esters is a well known protection strategy.^44^ Unfortunately, many of the methods used to prepared formate esters use drastic conditions and suffer from serious limitations which include heating the alcohol in 85% formic acid or employing uncommon reagents. Palumbo *et al.* exploited PS-TPP–halogen complexes in the synthesis of formic acid esters from various primary and secondary alcohols (Table 12).^45^ It is noted that tertiary alcohols under the same conditions failed to give the desired formate esters and instead afforded the alkyl halides. Both iodine and chlorine complexes (PS-TPP–I_2_ and PS-TPP–Cl_2_) were suitable, whereas the bromine complex (PS-TPP–Br_2_) was not tolerated at least with unsaturated alcohols due the co-occurrence of a side reaction involving some bromination of double bonds. The iodine complex was the most convenient considering the ease of handling compared with chlorine.

**Table 12:** Conversion of alcohols into formate esters by PS-TPP–I_2_–dimethylformamide adducts^a^

Entry	Alcohol	Formate ester	Yield (%)
1	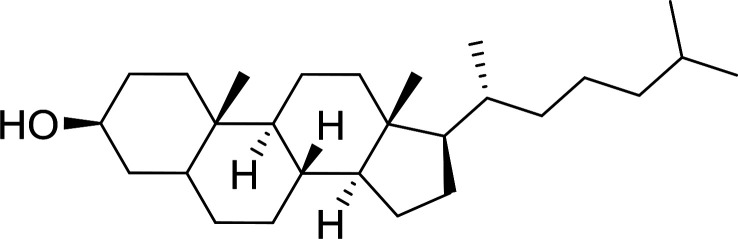	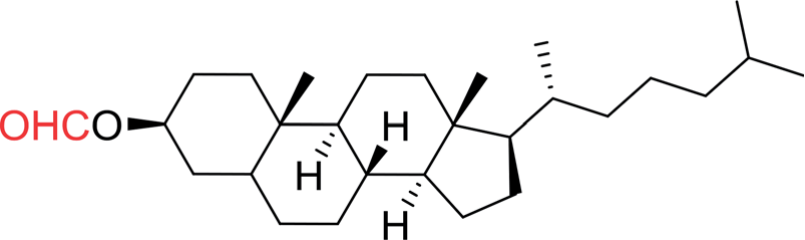	93
2	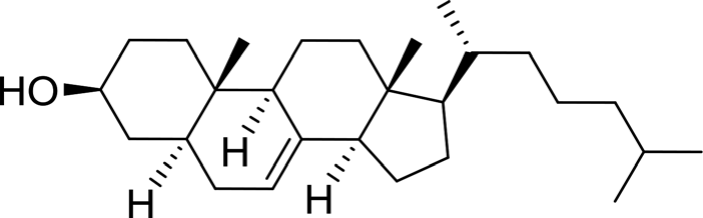	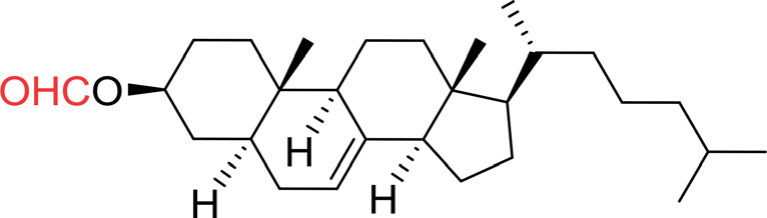	90
3	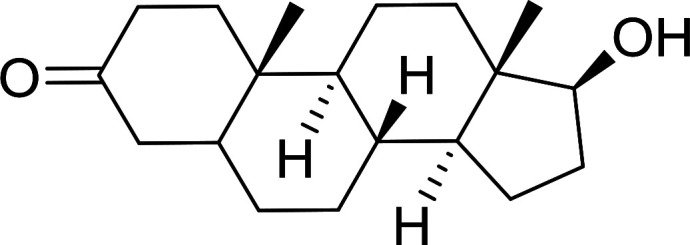	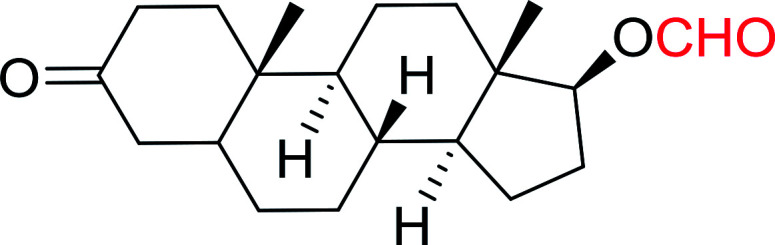	90
4	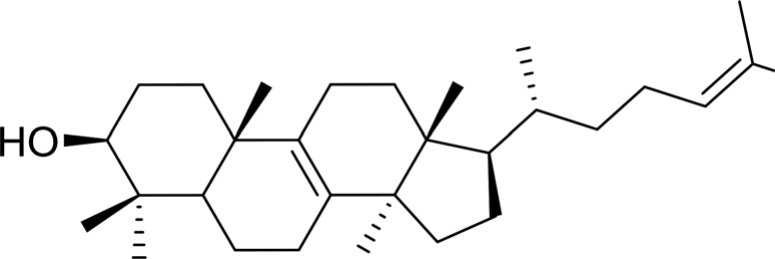	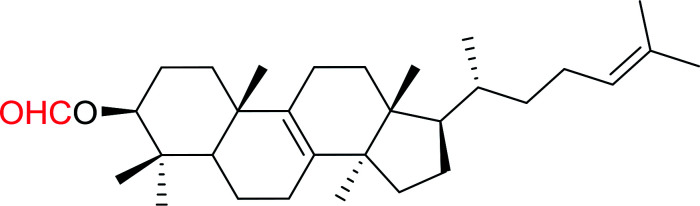	92
5	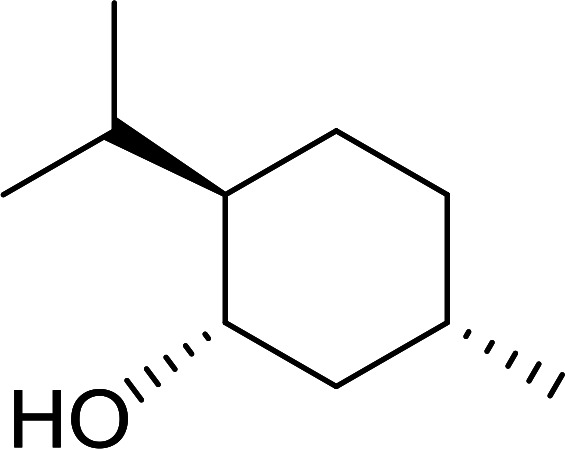	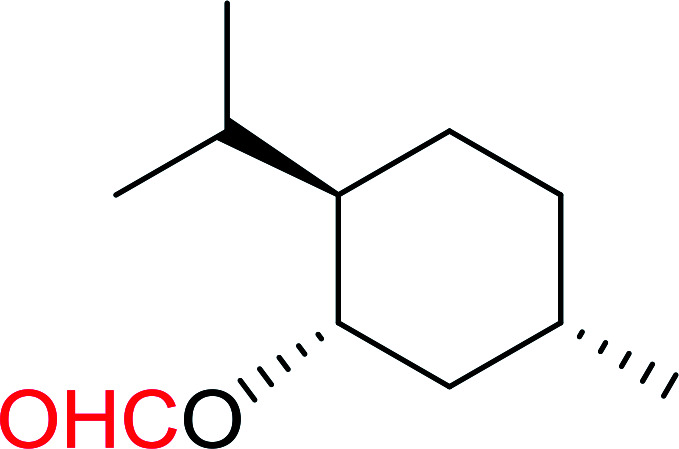	78
6	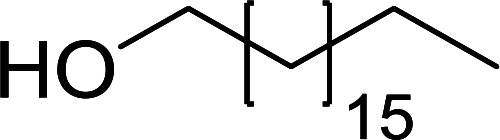	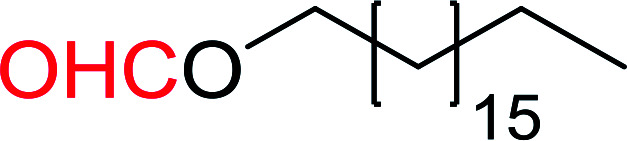	84
7	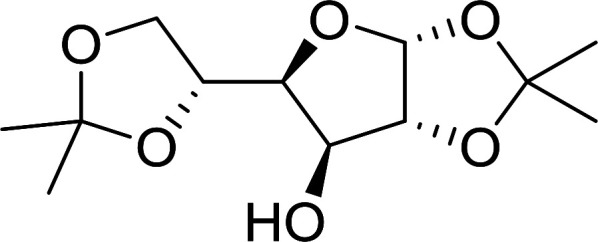	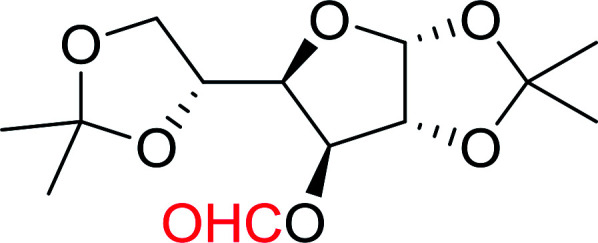	92

a Reagents and conditions: 2.3 : 2.3 : 2.4 : 1 molar ratios of PS-TPP : I_2_ : DMF : alcohol, RT, 30 min.

#### 3.2.12. Acetalization of carbonyl compounds with PS-TPP–I_2_ complex

**Scheme 9:**
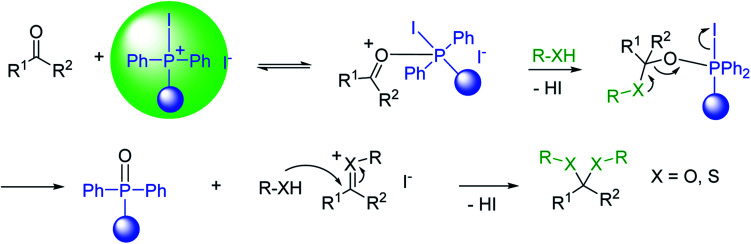
Mechanism of acetalization of carbonyl compounds using PS-TPP–I_2_.

Cyclic and acyclic acetals, dithioacetals, and oxathioacetals are very common groups used to protect the carbonyl function of aldehydes and ketones.^46^ One drawback of acetalization, besides using alcoholic media and Brønsted acid catalysts, is the generation of water during the reaction and the need to remove it by physical or chemical means to drive the equilibrium forward. Thus, a strategy to prepare acetals from carbonyl compounds and alcohol, diols, dithiols, or hydroxythiols under anhydrous conditions and devoid of water formation has been described using the combination of PS-TPP and I_2_.^47^ In this approach, the carbonyl compound in anhydrous acetonitrile is treated with a pre-formed PS-TPP–I_2_ complex, followed by the desired protecting reagent. An adduct is formed between the positively charged phosphorus atom and carbonyl oxygen, thus activating the carbonyl carbon center for nucleophilic addition by a molecule of the protecting reagent (Scheme 9). A subsequent non-equilibrium step involves the elimination of PS-TPPO and generation of an oxygen-stabilized carbocation which undergoes further reaction with a second reagent molecule or a tethered nucleophile on the same reagent to afford the final product.

The devised procedure for acetalization tolerates aliphatic and aromatic aldehydes and ketones, as shown in Table 13. It is noted that in order to avoid acidic medium for certain substrates, anhydrous Et_3_N was added in portions throughout the reaction.

**Table 13:** Acetalization reactions of ketones and aldehydes catalysed by PS-TPP–I_2_ complex^a^

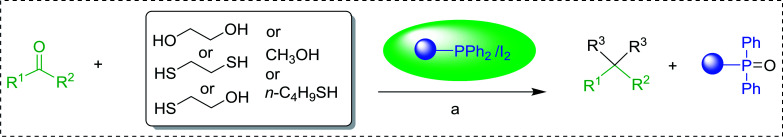				
Entry	R^1^	R^2^	R^3^	Yield (%)
1	Ph	Me	–O-(CH_2_)_2_-O–	80
2	Ph	Me	–S-(CH_2_)_2_-S–	98
3	Ph	Me	–S-(CH_2_)_2_-O–	92
4	Ph	Ph	–S-(CH_2_)_2_-S–	86
5	Ph	Ph	–S-(CH_2_)_2_-*O*-	88
6	Ph	H	–S-(CH_2_)_2_-S–	98
7	Ph	H	–S-(CH_2_)_2_-O–	85
8	*n*-C_7_H_15_	H	–O-(CH_2_)_2_-O–	84
9	*n*-C_7_H_15_	H	–S-(CH_2_)_2_-S–	98
10	*n*-C_7_H_15_	H	–S-(CH_2_)_2_-O–	85
11	–(CH_2_)_2_CH(*t*-C_4_H_9_)(CH_2_)_2_		*n*-C_4_H_9_S–	80
12	–(CH_2_)_2_CH(*t*-C_4_H_9_)(CH_2_)_2_		CH_3_O–	92

a Reagents and conditions: 1.2 : 1 : 4 molar ratio of PS-TPP–I_2_ : carbonyl compound : protecting reagent, Et_3_N (for entries 1, 3, 8 and 12), MeCN, RT, 40 min.

#### 3.2.13. Polymer-supported triphenylphosphine dibromide (PS-TPP–Br_2_–imiH) and diiodide (PS-TPP–I_2_–imiH): conversion of alcohols into alkyl iodides

**Scheme 10:**
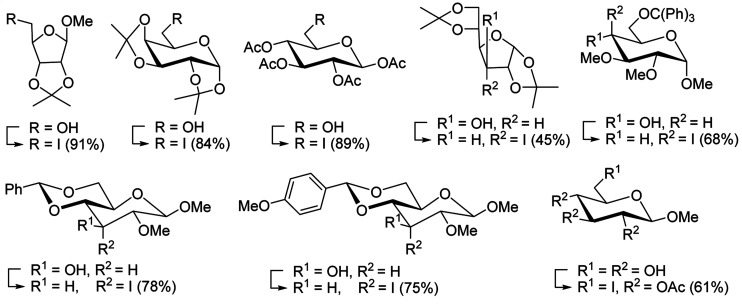
Conversion of alcohols into alkyl iodides with PS-TPP–I_2_ complex. Reagents and conditions: 3 : 1.3 : 3 : 1 molar ratios of PS-TPP : I_2_ : imidazole : alcohol, toluene, 50–120 °C, 3–8 h.

Building on Hodge's earlier work regarding the use of polymer-supported triphenylphosphine dibromide (PS-TPP–Br_2_) in the conversion of alcohols into alkyl bromides,^36^ Classon prepared a similar polymer-supported phosphine–halogen complex using iodine in combination with imidazole (PS-TPP–I_2_–imiH).^48^ The addition of imidazole enhanced reactivity and neutralized liberated HI, resolving reactivity and yield issues encountered earlier by Hodge *et al.* in the bromination of alcohols using the related PS-TPP–Br_2_ complex.^36^ Other bases such as pyridine or Et_3_N were examined, although much lower yields were generally obtained. The halogenation reactions were performed in heated toluene which provided a two phase liquid–liquid reaction system and promoted faster reactivity than other solvents because the non-polar character of the solvent appears to destabilize the charged phosphonium halide intermediate and drive decomposition to the uncharged alkyl iodide and phosphine oxide byproduct. The iodinations examined involved the synthesis of iodides from alcohols, using various carbohydrates as substrates. Acid-sensitive groups such as acetals, glycosidic linkages, and triphenylmethyl ethers remained intact under reaction conditions (Scheme 10). However unprotected polyhydroxy sugars were unsuitable as substrates and gave complicated reaction mixtures.

#### 3.2.14. Synthesis of (*E*)-nitroalkenes from aldehydes and nitroalkanes PS-TPP and I_2_

Bez *et al.* described the first solid phase one-pot procedure for the synthesis of (*E*)-nitroalkenes 38 (ref. 49) by reacting diverse aromatic, aliphatic, and α,β-unsaturated aldehydes (36) with nitroalkanes (37) in the presence of polymer-bound triphenylphosphine, iodine and imidazole (Table 14). Although the same transformation proceeded with similar efficiency using triphenylphosphine instead of its polymer-bound analogue, easy removal of the unwanted polymer-bound triphenylphosphine oxide provided the edge for practical application of the protocol. The reaction generally gave good yields except with less electrophilic aldehydes such as 3,4-dimethoxybenzaldehyde (entry 8) and 3,4-methylenedioxybenzaldehyde (entry 9) which also required longer reaction time (3 h) for complete conversion.

**Table 14:** Synthesis of (*E*)-nitroalkenes from aldehydes and nitroalkanes^a^

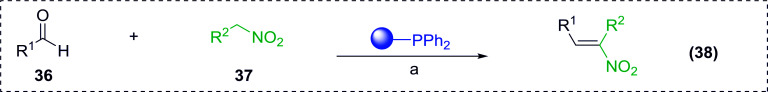				
Entry	Aldehyde	R^2^	Nitroalkene product	Yield (%)
1	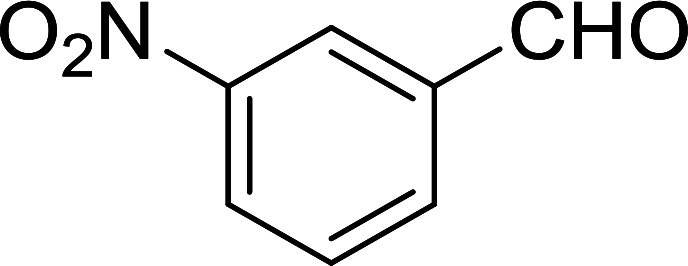	H	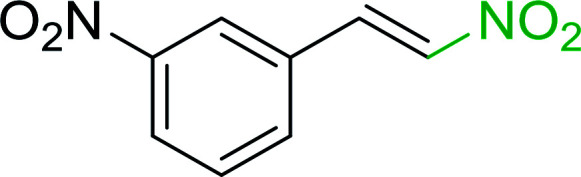	93
2	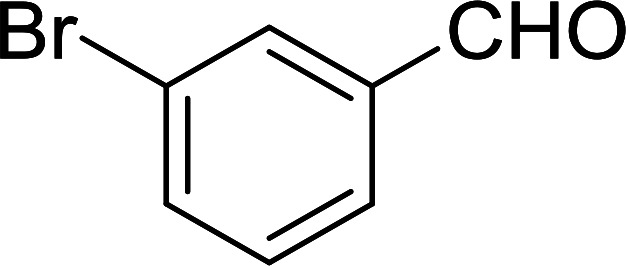	CH_3_	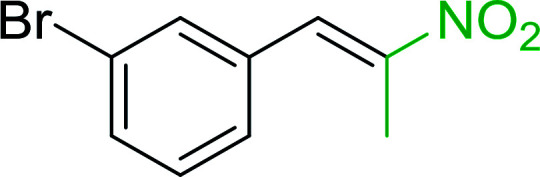	89
3	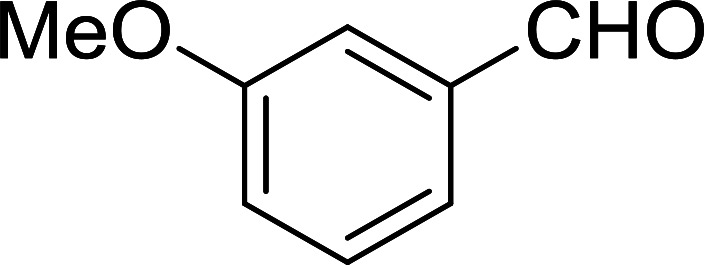	H	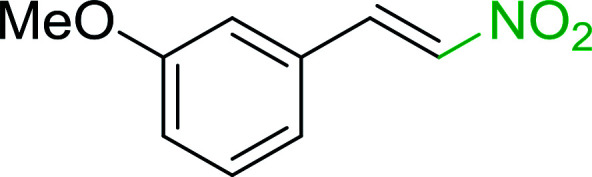	84
4	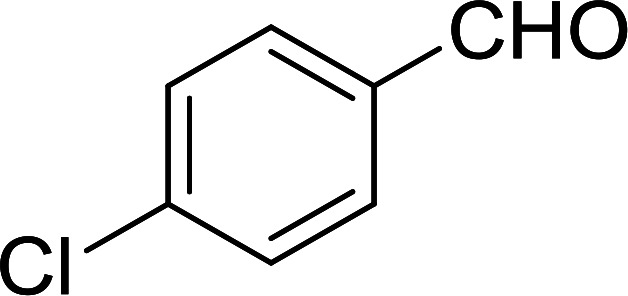	H	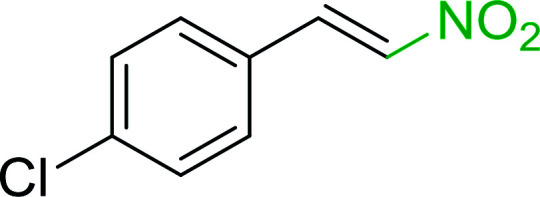	93
5	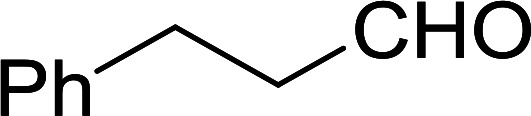	CH_3_	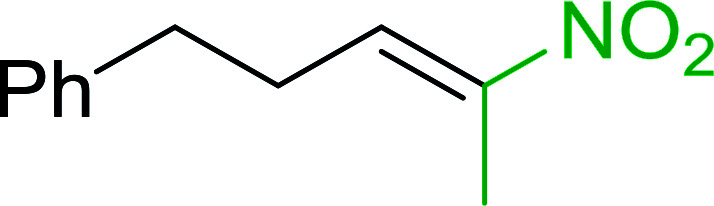	89
6	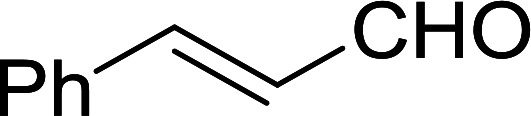	CH_3_	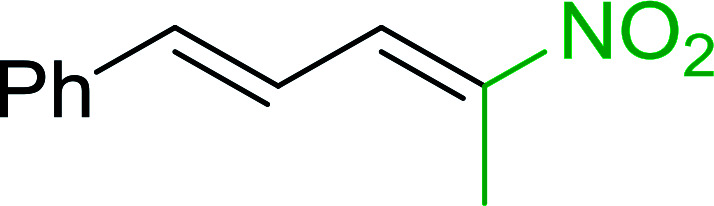	89
7	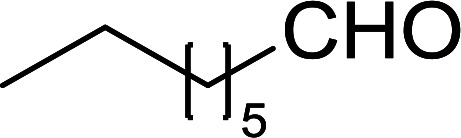	H	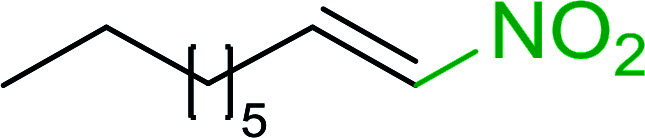	81
8	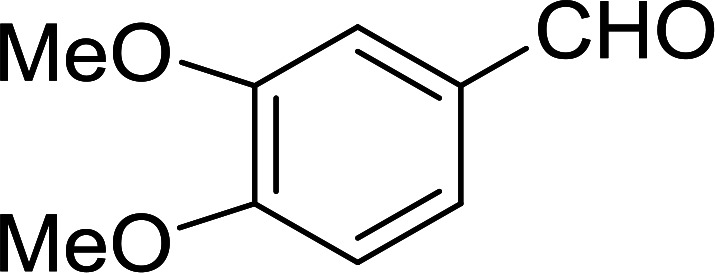	H	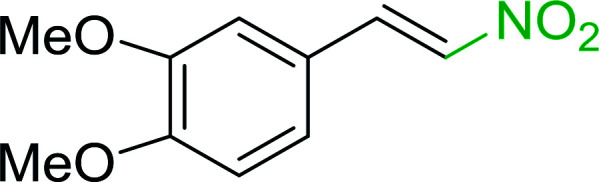	55
9	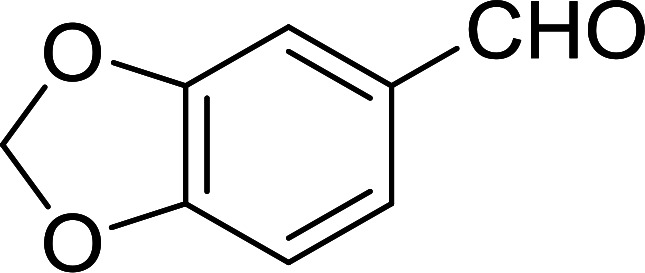	CH_3_	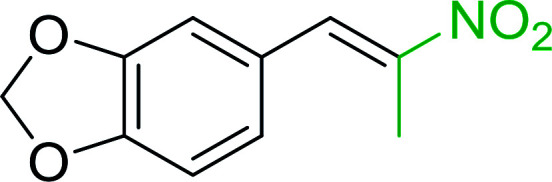	58

a Reagents and conditions: 1 : 1.5 : 1.5 : 1.5 : 2 molar ratios of aldehyde : nitroalkane : PS-TPP : iodine : imidazole, RT, CH_2_Cl_2_, 1–3 h.

#### 3.2.15. Preparation of *N*-protected β-iodoamines from β-amino alcohols using PS-TPP–I_2_ complex

Enantiomerically pure *N*-protected β-iodoamines are important synthetic precursors used for the preparation of d- or l-β-amino acids, which themselves represent challenging synthetic targets.^50^ One approach to the preparation of β-amino acids consists of homologating the requisite α-aminoacid, whereby the α-carboxyl group gets converted into an alkyl halide in preparation for the subsequent homologation step. Longobardo *et al.* developed a general synthesis of chiral *N*-protected β-iodoamines 40, possessing either d- or l-configuration, by treating the precursor chiral *N*-protected β-aminols 39 with PS-TPP–I_2_ complex.^51^ The β-aminoalcohols were accessed from *N*-protected α-aminoacid or commercial β-aminols. As shown in Table 15, the conversion was effective for β-aminols protected with the most frequently used *N*-protecting groups (Cbz, Boc, Fmoc) without any detectable racemization of the stereocenter. The utility of this methodology was later demonstrated by Longobardo *et al.* in the synthesis of enantiopure *N*- and *C*-protected *homo*-β-amino acids by direct homologation of α-aminoacid.^52^ The devised process involved reducing the carboxyl group of *N*-protected α-amino acids and converting the resulting *N*-protected β-amino alcohols into the corresponding β-iodoamines with PS-TPP–I_2_, which represented the key step of the strategy. Subsequent cyanation and acidic hydrolysis of the β-amino cyanides afforded enantiopure *homo*-β-amino acids.

**Table 15:** Synthesis of *N*-protected β-iodoamines using PS-TPP–I_2_ complex^a^

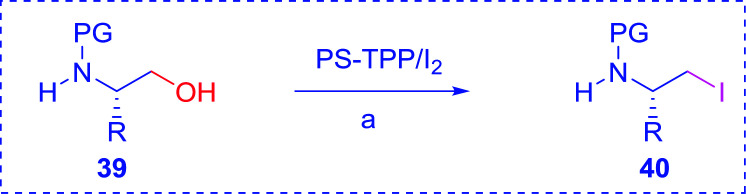			
Entry	PG	R	Yield (%)
1	Cbz	Me	89
2	Cbz	Bn	90
3	Boc	CH(CH_3_)_3_	90
4	Boc	CH_2_COOBn	82
5	Boc	Ph	95
6	Fmoc	Me	92
7	Fmoc	Bn	94
8	Fmoc	CH_2_CH(CH_3_)_3_	94

a Reagents and conditions: 2.2 : 2.5 : 1 molar ratios of PS-TPP/I_2_ : imidazole : alcohol, CH_2_Cl_2_, reflux, 1 h.

#### 3.2.16. Peptide synthesis using PS-TPP–I_2_ complex

The utility of the polystyryl diphenylphosphine–iodine complex (PS-TPP–I_2_) has also been showcased in peptide synthesis.^53^ The action of the complex involves converting the free carboxylic acid group of a *N*-protected α-amino acid into an acyl donor intermediate, followed by nucleophilic substitution by the amino function of a second, *C*-protected α-amino acid, affording fully protected dipeptides. Using this approach, Longobardo and coworkers were able to couple various *N*-protected α-amino acid with α-aminoacyl esters, by using PS-TPP–I_2_, in high yields and without detectable racemization of the reacting substrates (Table 16).^53^ Neutral reaction conditions were secured by the addition of excess imidazole to neutralize the released hydrogen iodide. Thus, the process was effective for substrates carrying the common *N*-protecting groups (Fmoc, Boc, Cbz) and tolerated acid sensitive *S*- and *O*-protecting groups like the trityl (entry 3), methyl (entries 2 and 3), *tert*-butyl (entry 4), and benzyl groups (entry 5).

**Table 16:** Preparation of dipeptides with polymeric triphenylphosphine and I_2_^a^

Entry	*N*-Protected α-AA	*O*-Protected α-AA	Dipeptide	Yield (%)
1	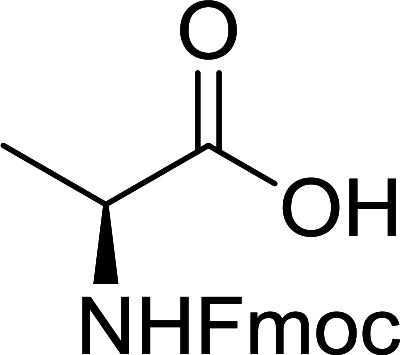	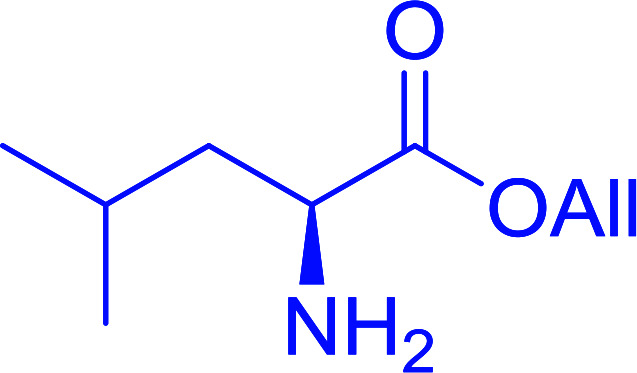	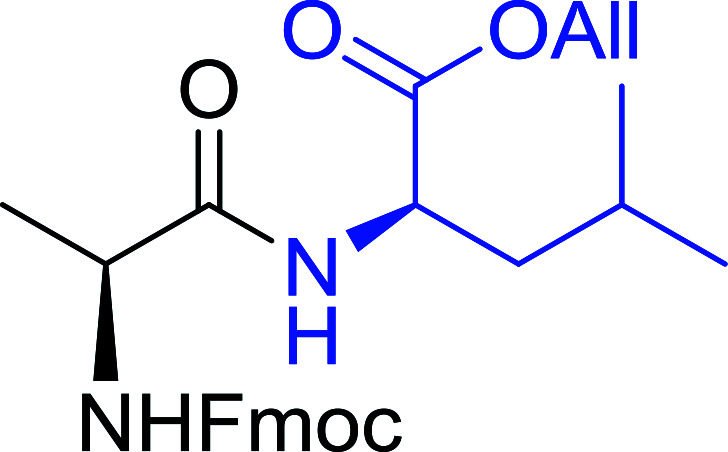	99
2	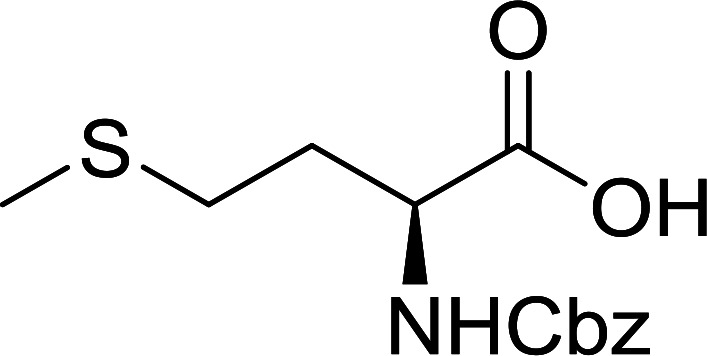	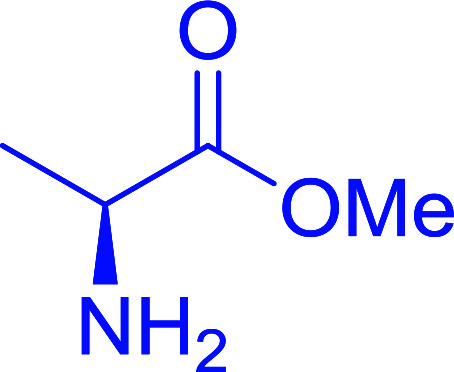	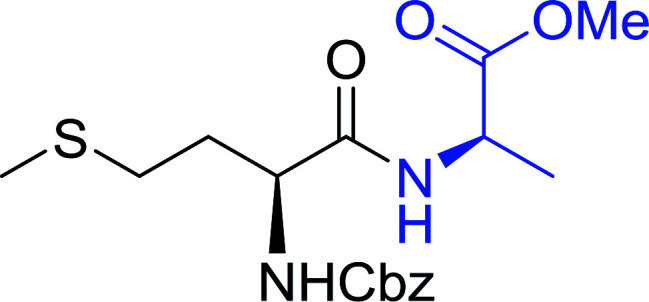	98
3	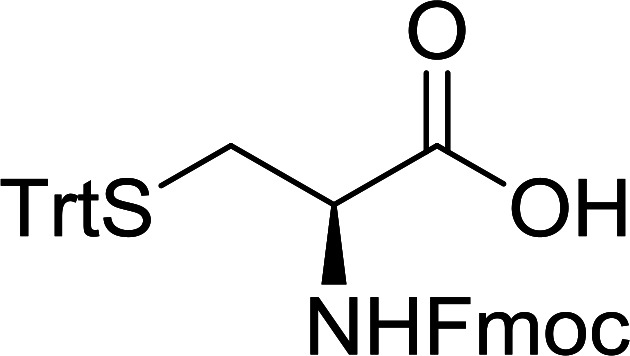	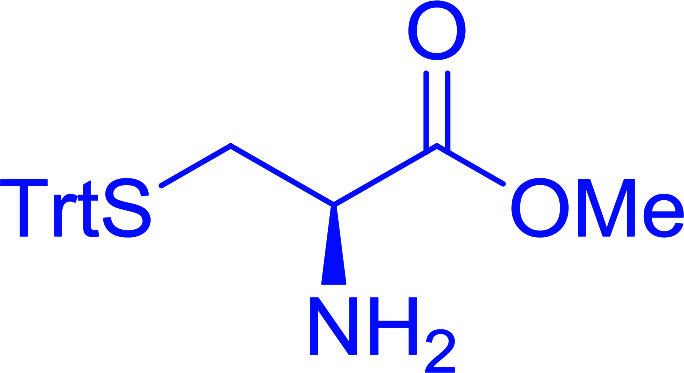	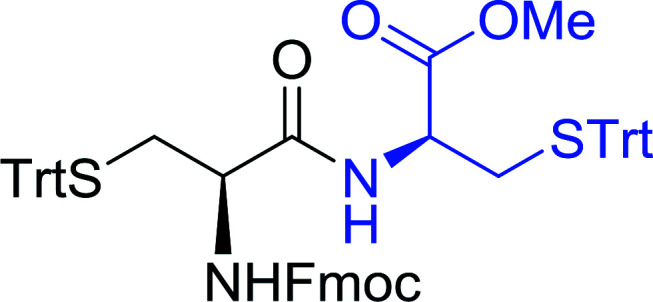	95
4	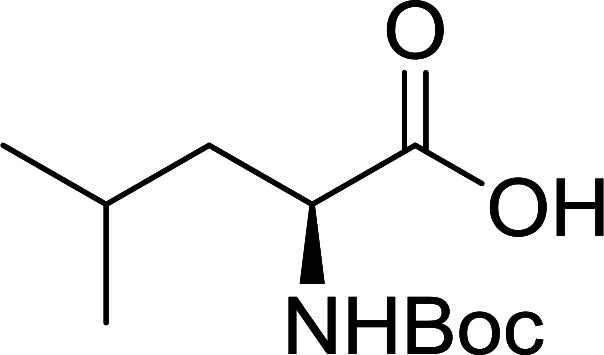	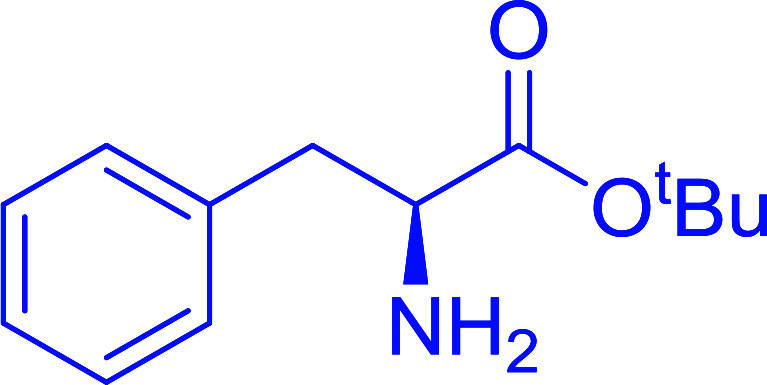	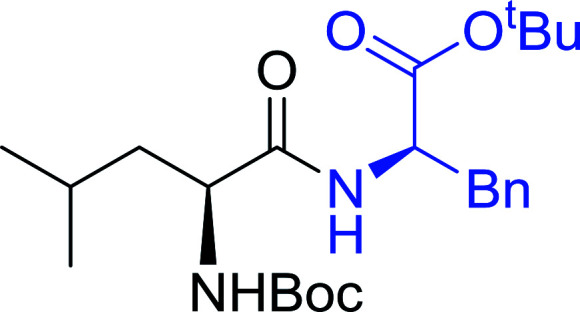	95
5	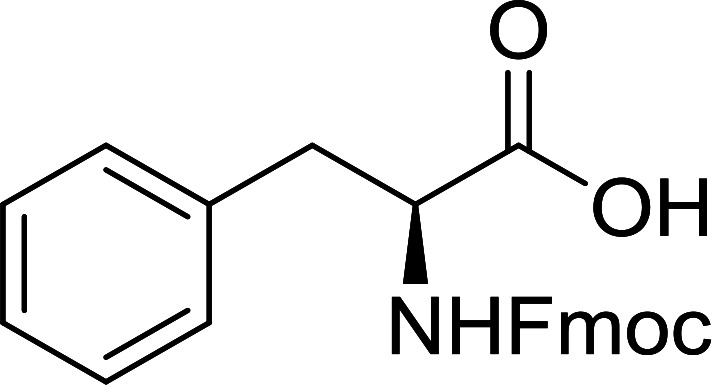	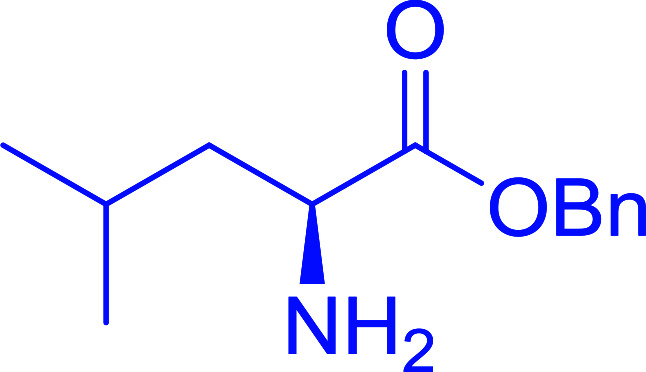	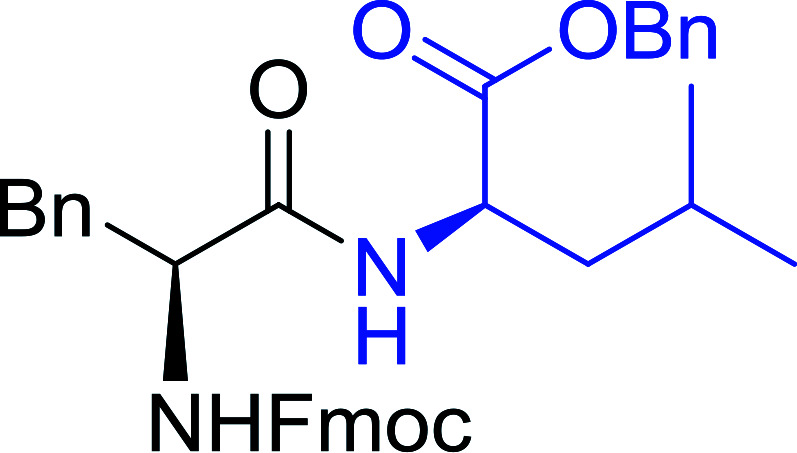	95

a Reagents and conditions: 2.2 : 2 : 1 : 1 : 2.5 molar ratios of PS-TPP : I_2_ : *N*-protected α-amino acid : *O*-protected α-amino acid : imidazole, CH_2_Cl_2_, RT, 30 min.

#### 3.2.17. Monoesterification of symmetric diols using PS-TPP–I_2_ complex

The preparation of carboxylic acid esters from di- or polyhydroxylated substrates is a challenging synthetic task and the introduction of reliable strategies towards the selective monoesterification of such alcohols is useful.^54^ Rokhum and Pathak reported the use of PS-TPP/iodine in coupling reactions between carboxylic acids and alcohols or amines to produce esters and amides, respectively.^55^ More importantly, the PS-TPP–I_2_ reagent has been shown to affect the monoesterification of symmetrical diols without resorting to high dilution or slow addition conditions. As shown in Table 17, the monoesterification of 1,6-hexanediol (entries 1–5) and 1,8-hexanediol (entries 6–8) with various carboxylic acids was achieved in high yields (84–92%) in the presence of DMAP and preformed PS-TPP–I_2_ complex.

**Table 17:** Preparation of monoesters from diols using PS-TPP and iodine^a^

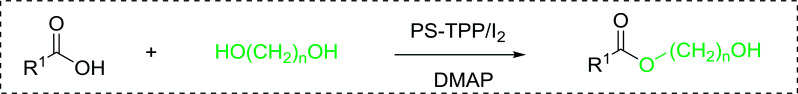				
Entry	Alcohol	Carboxylic acid	Product	Yield (%)
1	HO(CH_2_)_6_OH	Benzoic acid	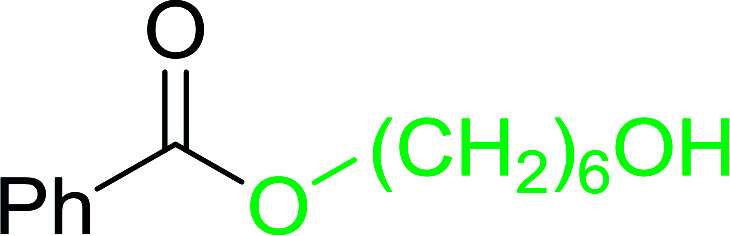	88
2		Propanoic acid	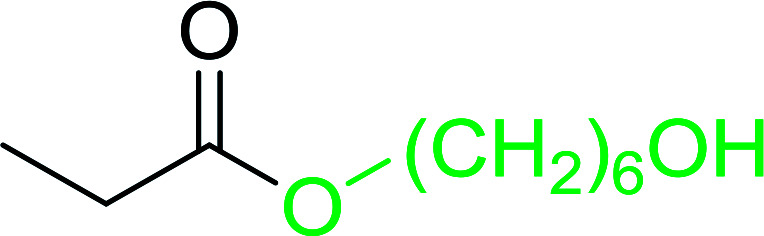	91
3		Pentanoic acid	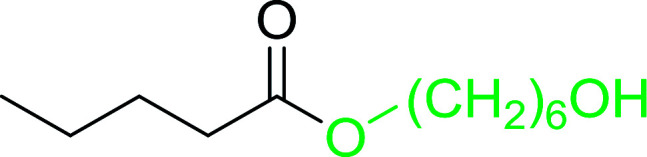	87
4		4-Methylbenzoic acid	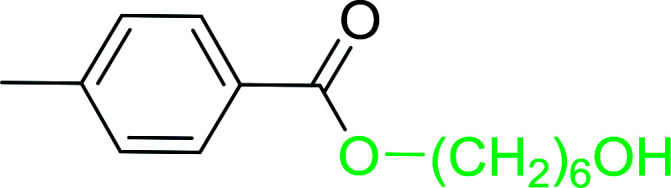	84
5		4-Nitrobenzoic acid	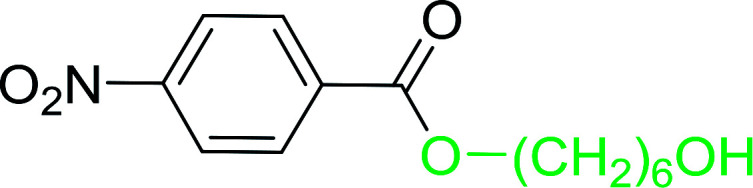	92
6	HO(CH_2_)_8_OH	3-Nitrobenzoic acid	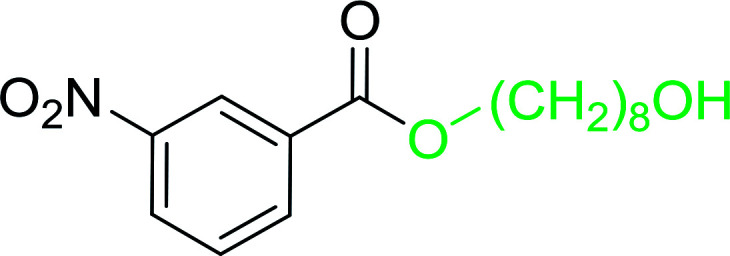	84
7		Benzoic acid	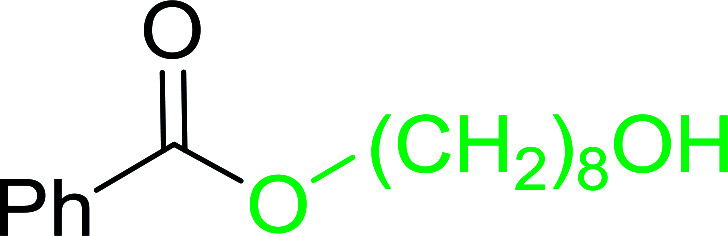	85
8		Lauric acid	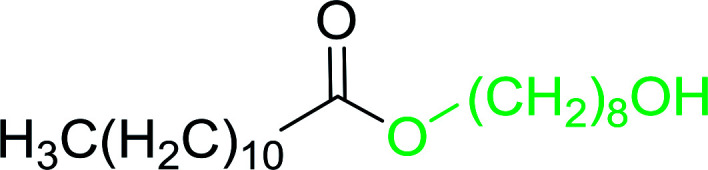	91

a Reagents and conditions: 1.5 : 1.5 : 1 : 1 : 3 molar ratios of PS-TPP/I_2_ : alcohol : carboxylic acid : DMAP, THF/CH_2_Cl_2_ (1 : 3 v/v), RT, 18–22 h.

#### 3.2.18. Iodination, bromination, and chlorination of alcohols with PS-TPP/X_2_ and PS-TPP/NCS imidazole systems

Building on the previous reports describing the use of PS-TPP and iodine for the conversion of α-amino acids and sugars into the corresponding iodides,^48,52,56^ Kita *et al.* extended the methodology to the halogenation of alcohols under new conditions.^57^ The reagent system was prepared by treating a suspension of PS-TPP in CH_2_Cl_2_ at room temperature with imidazole and iodine, bromine, or *N*-chlorosuccinimide (NCS). Subsequent addition of various allylic, benzylic, and primary alcohols gave the halogenated products in very high yields (88–98%) (Table 18). The reactions worked well with *ortho*, *meta*, *para*-, and multisubstituted benzylic alcohols (entries 2–9), as well as allylic (entries 10–12) and simple alcohols (entries 1, 13 and 14). While the iodination and bromination reactions were complete within 2 h in all cases, the chlorination reaction (entry 14) required 24 h for complete conversion. Finally, when the reagent system was tested on secondary alcohols, the reaction was very slow and unpractical for such systems.

**Table 18:** Halogenation of alcohols^a^

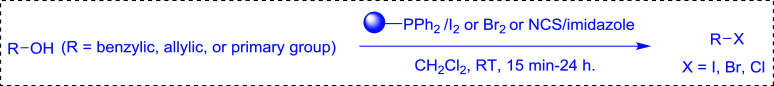			
Entry	Alcohol	Alkyl halide	Yield (%)
1	Benzyl alcohol	Benzyl iodide	97
2	*o*-Tolylmethanol	1-(Iodomethyl)-2-methylbenzene	95
3	(2-Bromophenyl)methanol	1-Bromo-2-(iodomethyl)benzene	95
4	(2-Methoxyphenyl)methanol	1-(Iodomethyl)-2-methoxybenzene	96
5	3-(Hydroxymethyl)benzonitrile	3-(Iodomethyl)benzonitrile	98
6	(3-Nitrophenyl)methanol	1-(Iodomethyl)-3-nitrobenzene	98
7	(4-Methoxyphenyl)methanol	1-(Iodomethyl)-4-methoxybenzene	92
8	(2,4-Dichlorophenyl)methanol	1-(Iodomethyl)-2,4-dichlorobenzene	97
9	(2,4-Dimethoxyphenyl)methanol	1-(Iodomethyl)-2,4-dimethoxybenzene	95
10	(*E*)-3-Phenylprop-2-en-1-ol	(*E*)-(3-Iodoprop-1-en-1-yl)benzene	95
11	(*Z*)-3,7-Dimethylocta-2,6-dien-1-ol	(*Z*)-1-Iodo-3,7-dimethylocta-2,6-diene	91
12	(*E*)-3,7-Dimethylocta-2,6-dien-1-ol	(*E*)-1-Iodo-3,7-dimethylocta-2,6-diene	88
13	*p*-Tolylmethanol	1-(Bromomethyl)-4-methylbenzene	96
14	*p*-Tolylmethanol	1-(Chloromethyl)-4-methylbenzene	90

a Reagents and conditions: entries 1–12; 1.3 : 1.3 : 1.3 : 1 molar ratios of PS-TPP : I_2_ : imidazole : alcohol, CH_2_Cl_2_, RT, 15–120 min; entry 13; same conditions except Br_2_ was used; entry 14; same conditions except NCS was used and the reaction was stirred for 24 h.

#### 3.2.19. Synthesis of glycosyl iodides

A new and stereoselective synthesis of α-d-glycosyl iodides obtained by the replacement of the free anomeric hydroxyl functional group of fully protected sugars using PS-TPP–iodine complex was reported by Caputo and co-workers.^56^ Thus, the addition of various protected sugars to a mixture of PS-TPP, iodine, and imidazole, at room temperature, resulted in the rapid conversion to the α-glycosyl iodide anomer (Table 19). No traces of the β-anomers were observed in any of the examples, suggesting that the process is thermodynamically controlled.

**Table 19:** Preparation of glycosyl iodides from protected sugars^a^

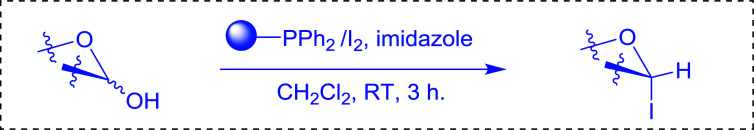			
Entry	Carbohydrate	Protection group	Yield (%)
1	d-Glucopyranose	2,3,4,6-Tetra-*O*-acetyl	97
2	d-Glucopyranose	2,3,4,6-Tetra-*O*-benzyl	95
3	d-Galactopyranose	2,3,4,6-Tetra-*O*-acetyl	95
4	d-Galactopyranose	2,3,4,6-Tetra-*O*-benzyl	92
5	d-Mannopyranose	2,3,4,6-Tetra-*O*-acetyl	95
6	d-Mannopyranose	2,3,4,6-Tetra-*O*-benzyl	94
7	d-Mannofuranose	2,3,5,6-Di-*O*-isopropylidene	98

a Reagents and conditions: 1.9 : 1.9 : 3.5 : 1 Molar ratios of PS-TPP : I_2_ : imidazole : sugar.

#### 3.2.20. Esterification of alkylphosphonic acids

Alkylphosphonates have many synthetic and pharmaceutical applications.^58^ In addition, methyl, ethyl, i-propyl and *n*-propyl esters of alkyl phosphonic acids are registered chemical warfare agents (CWAs).^59^ The related *O*,*O*′-dialkyl alkylphosphonates (DAPs) are very useful reference chemicals that have been used as markers of nerve agents.^60^ However, since they are also classified as CWAs, they are not available commercially and access to this class of compounds becomes inevitable during verification analysis. Dubey *et al.* reported a procedure for the preparation of DAPs by esterification of alkylphosphonic acids using primary alcohols, iodine, imidazole, and PS-TPP (Table 20).^61^ Pure phosphonate esters were obtained by vacuum distillation in 83–94% yield following removal of PS-TPP oxide and imidazolium iodide by filtration.

**Table 20:** Esterification of alkylphosphonic acids^a^

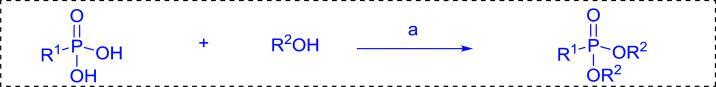				
Entry	R^1^	R^2^	Product	Yield (%)
1	CH_3_	CH_3_	*O*,*O*′-Dimethyl methylphosphonate	85
2	CH_3_	C_3_H_7_	*O*,*O*′-Dipropyl methylphosphonate	90
3	CH_3_	C_5_H_11_	*O*,*O*′-Dipentyl methylphosphonate	94
4	CH_3_	C_10_H_21_	*O*,*O*′-Didecyl methylphosphonate	82
5	C_2_H_5_	C_2_H_5_	*O*,*O*′-Diethyl ethylphosphonate	90
6	C_2_H_5_	C_5_H_11_	*O*,*O*′-Dipentyl ethylphosphonate	92
7	C_2_H_5_	C_10_H_21_	*O*,*O*′-Didecyl ethylphosphonate	84
8	C_3_H_7_	CH_3_	*O*,*O*′-Dimethyl propylphosphonate	87
9	C_3_H_7_	C_5_H_11_	*O*,*O*′-Dipentyl propylphosphonate	92
10	C_3_H_7_	C_10_H_21_	*O*,*O*′-Didecyl propylphosphonate	83

a Reagents and conditions: 1 : 3 : 6.6 : 3 : 2.5 molar ratios of alkyl phosphonic acid : iodine : imidazole : PS-TPP : alcohol, 45–50 °C, CH_2_Cl_2_, 2 h.

#### 3.2.21. Acetonation of sugars (protection of diols) with triphenylphosphine polymer-bound/iodine complex

The triphenylphosphine polymer-bound/iodine complex is a strong Lewis acid and a dehydrating agent as shown by the many transformations presented thus far.^62^ Palumbo *et al.* exploited this feature to prepare *O*-isopropylidene derivatives of various sugars by condensing the sugar with acetone.^63^ The isopropylidene function has been widely used in carbohydrate chemistry to protect diols and in certain cases sugar derivatives incorporating such a group have shown antipyretic and anti-inflammatory activities.^64^ The synthesis of the reagent complex involved adding a solution of iodine to an equimolar amount of polystyryl diphenylphosphine suspension at room temperature under dry N_2_ atmosphere and dark conditions. Subsequent addition of an acetone solution of the sugar to the suspension then afforded the thermodynamically more stable acetonides within 30 minutes in very high purity and yields (Table 21).

**Table 21:** Preparation of *O*-isopropylidene sugar derivatives^a^

Entry	Sugar	*O*-Isopropylidene derivatives	Yield (%)
1	d-Glucose	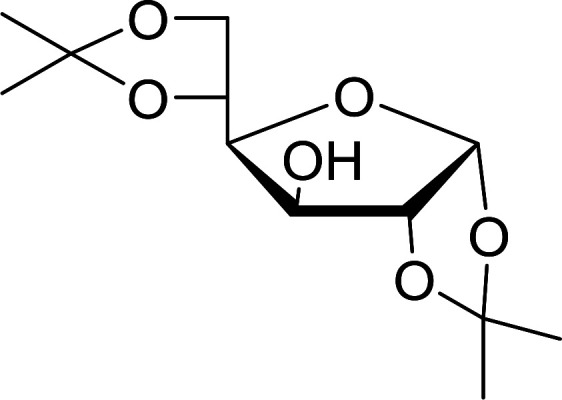	95
2	d-Ribose	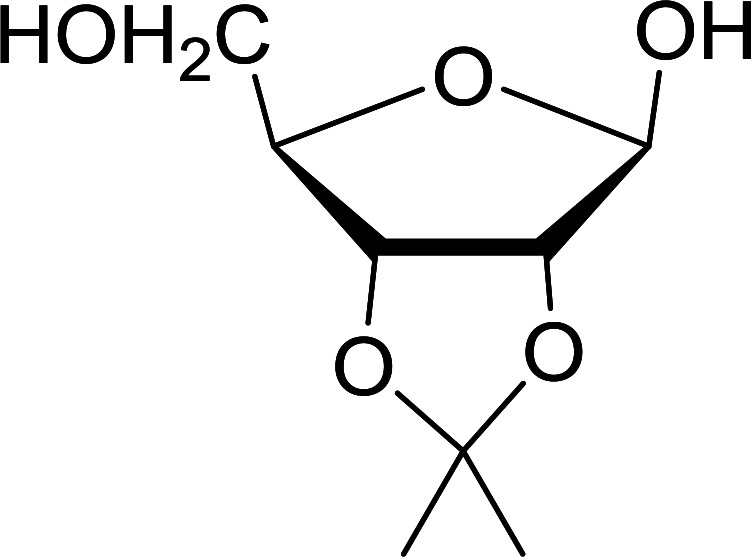	95
3	d-Mannose	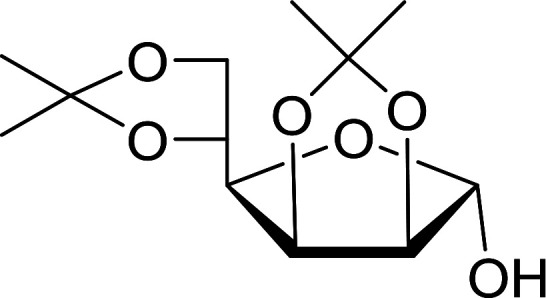	95
4	l-Arabinose	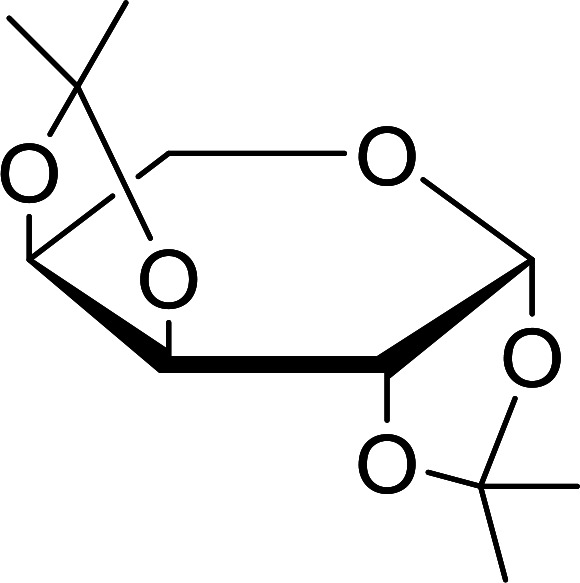	95
5	d-Galactose	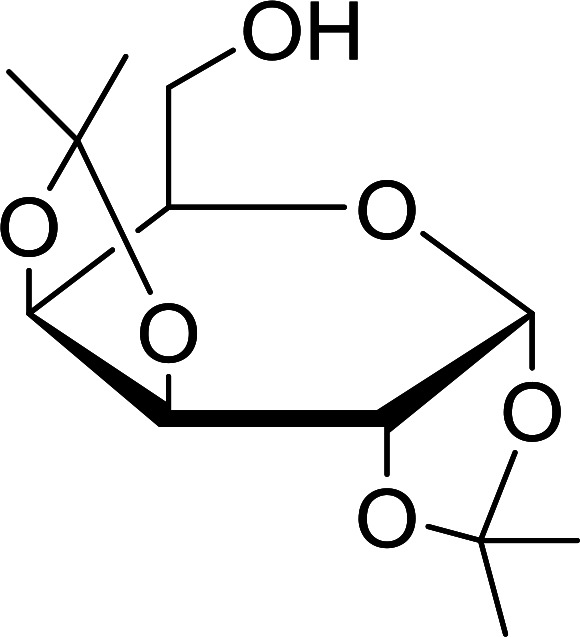	97

a Reagents and conditions: 2 : 2 : 1 molar ratios of PS-TPP : I_2_ : sugar, acetone, RT, 45 min.

#### 3.2.22. Conversion of alcohols to alkyl halides with catalytic amounts of PS-TPP

**Scheme 11:**
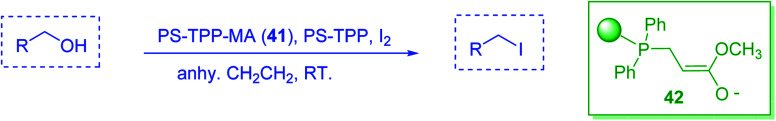
Solid phase conversion of alcohols to iodides using PS-TPP–MA and soluble Ph_3_P.

As shown earlier, Kita *et al.*^57^ had already reported a convenient method of iodination of alcohols using PS-TPP/iodine/imidazole reagent system. Rokhum reported a modification of Kita's method, switching imidazole with DMAP.^65^ However in both approaches the catalyst (imidazole/DMAP) was used in excess and the recovery and reuse of the reagent system was not possible. A major drawback of using equimolar amounts of PS-TPP for the halogenation of alcohols is the formation of polymer-supported triphenylphosphine oxide byproduct as the end of the reaction, which in principle may be recycled using reducing conditions.^66,67^ However, in order to avoid the inconvenience associated with recovering and recycling the polymer through reduction, Rokhum described a clever approach to the selective halogenation of alcohols using catalytic amounts of polymer-supported triphenylphosphine–methylacrylate complex 41.^68^ The protocol involves reactions of PS-TPP and methylacrylate in dichloromethane (10 mL) at room temperature for two minutes to generate *in situ* the triphenylphosphine–methylacrylate complex 42 (40 mol%). Subsequent addition of iodine and polymer supported triphenylphosphine, followed by the alcohol afforded the desired iodide products in 78–93% yields. The procedure was successful with primary aliphatic alcohols, as well as secondary and tertiary ones, although the latter took longer time from complete conversion. Allyl alcohols were also converted into the corresponding iodides without affecting the olefinic bonds. Direct bromination using the reported reaction conditions proceeded as well to afford the desired bromides in high yields (87–91%) (Scheme 11).

### 3.3. Application of polymer-supported phosphonium salts derived from PS-TPP as traceless linkers for solid phase synthesis of alkenyl, alkyl and heterocyclic products

**Scheme 12:**
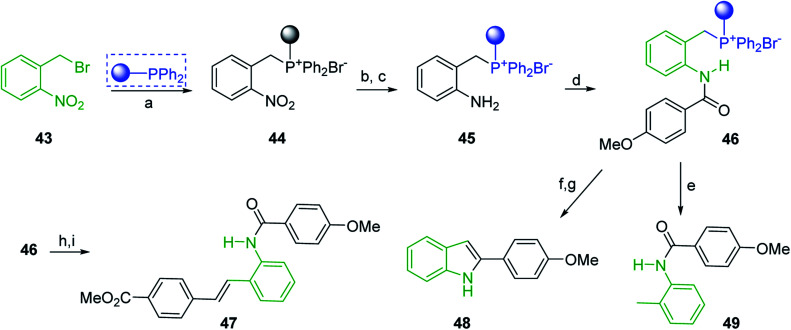
Solid-phase synthesis polymer-supported phosphonium salts derived from PS-TPP. Reagents and conditions: (a) DMF, 70 °C, 48 h; (b) Na_2_S_2_O_4_, EtOH, reflux, 90 min; (c) HBr, MeOH, dioxane; (d) 4-methoxybenzoyl chloride, pyridine, CH_2_Cl_2_, 5 h; (e) NaOMe, MeOH, reflux, 4 h, 81%; (f) toluene, DMF, distill; (g) KO*t*Bu, reflux, 45 min, 78% for both steps; (h) methyl 4-formylbenzoate (2 eq.), NaOMe (2 equiv.), MeOH, reflux, 2 h; (i) Girard's Reagent T (3 equiv.), AcOH, 18 h, 5 was produced in 82% overall yield as a 3 : 1 mixture of *E*/*Z* isomers.

Solid-phase synthesis of small organic molecule libraries for drug discovery programs has been one of the key driving forces responsible for the rapidly expanding field of combinatorial chemistry.^69^ Consequently, a large number of polymeric supports have been devised to immobilise compounds *via* polar functional groups such as alcohols, amides, and carboxylic acids.^69^ With the growing need to access a diverse range of small molecules, additional linker groups were required to meet the demand. One particular type, termed “traceless” linkers, leave no remnants in the cleaved product of the functionality utilized to tether the substrate to its support. Hughes showcased the utility of polymer-bound phosphonium salts derived from PS-TPP as traceless linkers for the solid-phase synthesis of small molecules.^70^ Thus, as shown in Scheme 12, the phosphonium salt 44 was prepared from 2-nitrobenzyl bromide (43) and PS-TPP, followed by reduction of the nitro group to generate the polymer-bound aniline 45. Finally, the aniline was acylated with 4-methoxybenzoyl chloride to afford anilide 46. The polymer-supported substrate 46 was elaborated and cleaved, *via* hydrolysis or an intramolecular Wittig/cyclization reaction in a traceless manner, to afford alkenyl, heteroaryl, and alkyl products (47–49) in 78–82% yield.

### 3.4. Staudinger reaction

#### 3.4.1. Reduction of nucleoside azides to amines

Functionalization of nucleosides by converting the sugar moiety into an aminosugar has been extensively studied.^71,72^ Reducing an azido function attached to the carbohydrate part represents one of the most convenient means for the preparation of the amine group.^72^ Building on this early work, Holletz and Cech described the reduction of nucleoside azides to amines under mild conditions and good yields using PS-TPP (Table 22).^73^ The reaction was executed in two steps using pyridine or dioxane as solvents. In the first step, reaction of PS-TPP with the azido nucleoside afforded an iminophosphorane, which upon subsequent hydrolysis in the second step with water or concentrated ammonia, led to the formation of the amine. While water hydrolysis left the base protecting group intact (entries 1, 2 and 3), ammonia was used in cases where simultaneous hydrolysis and *N*-deprotection was desired (entries 5 and 6). The reaction workup required only filtration of the suspended PS-TPPO and evaporation of the filtrate *in vacuo* to afford pure products in nearly quantitative yields (89–100%).

**Table 22:** Reduction of azido nucleoside to amines using PS-TPP^a^

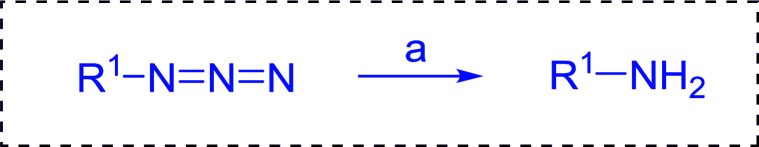			
Entry	Azido nucleoside	Amine product	Yield (%)
1	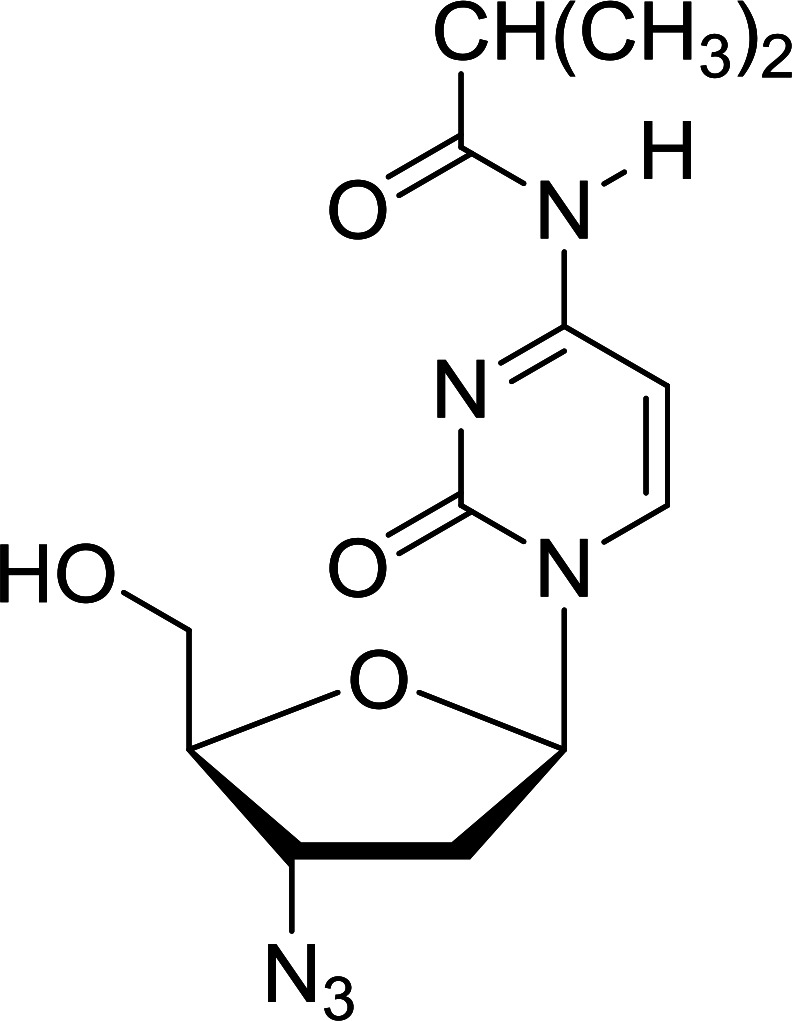	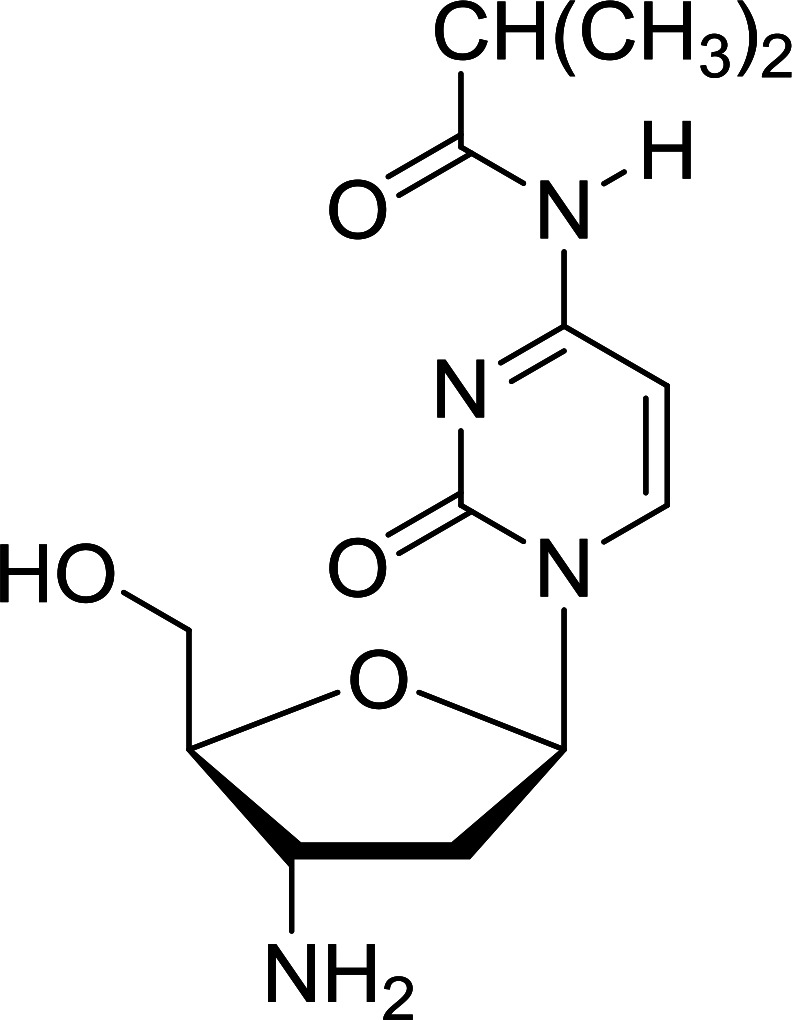	98
2	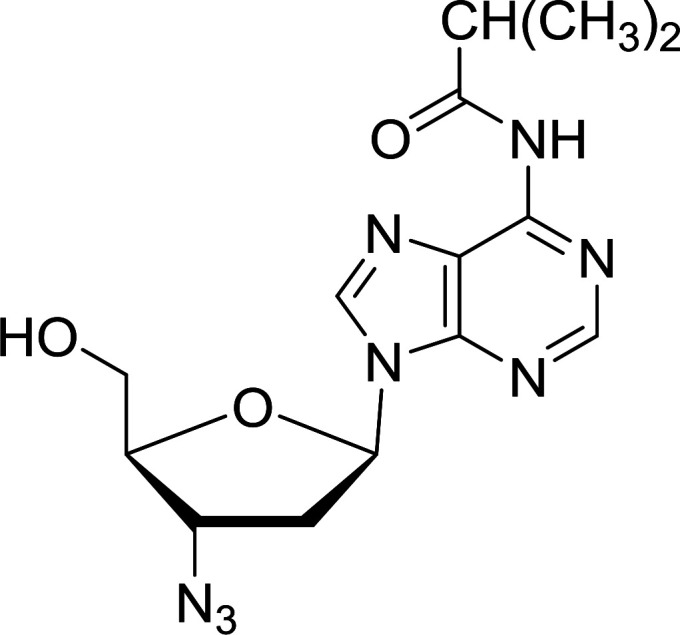	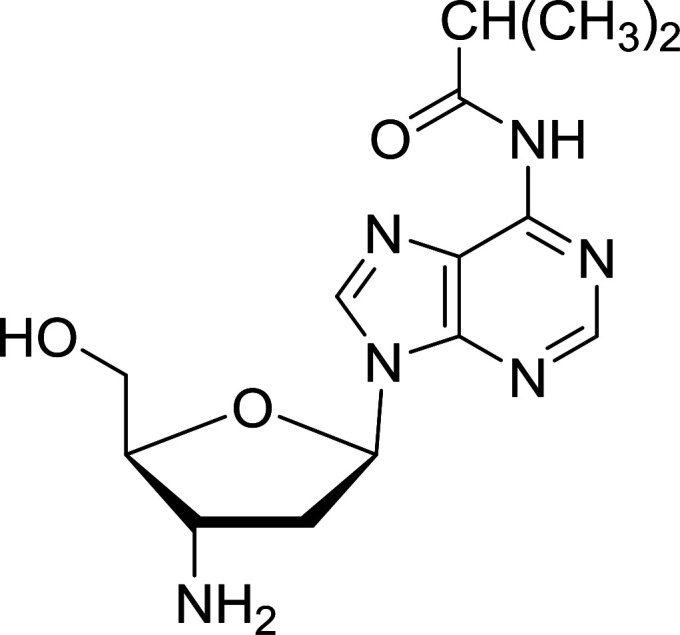	100
3	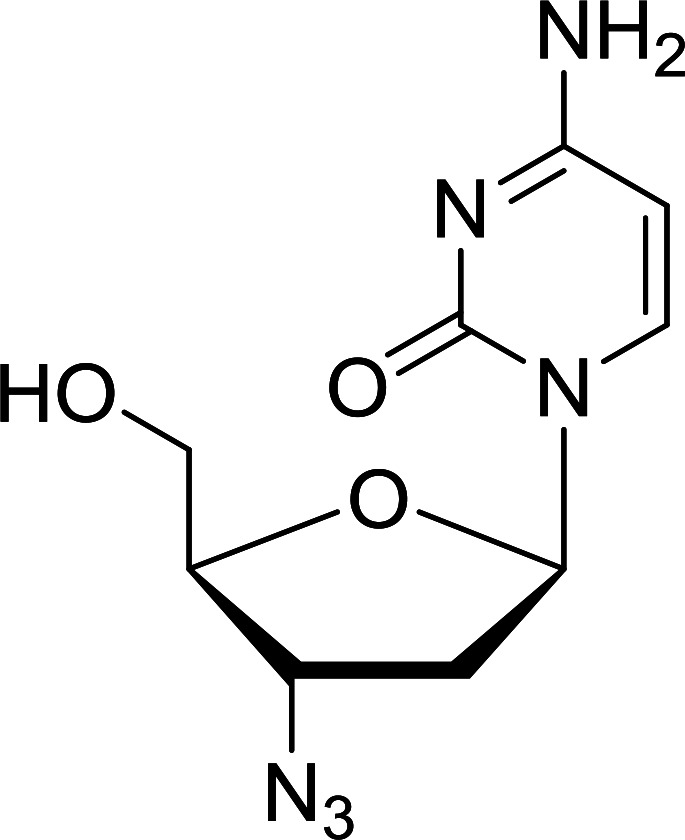	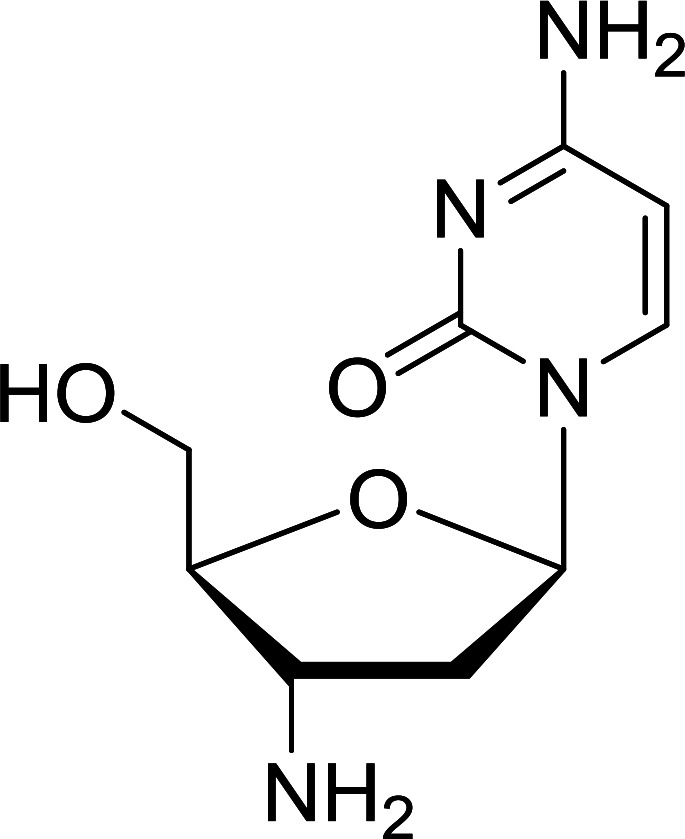	100
4	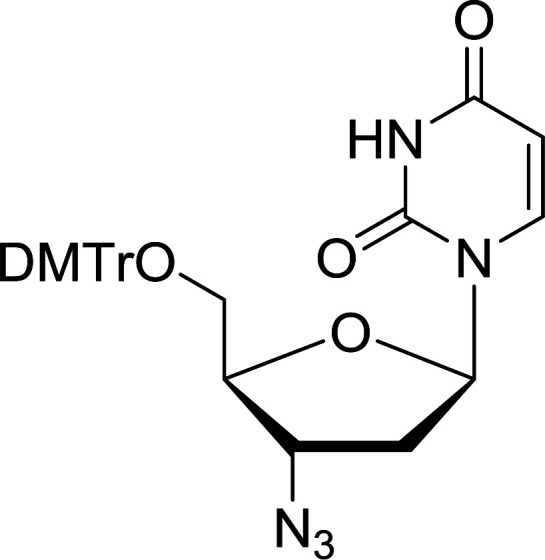	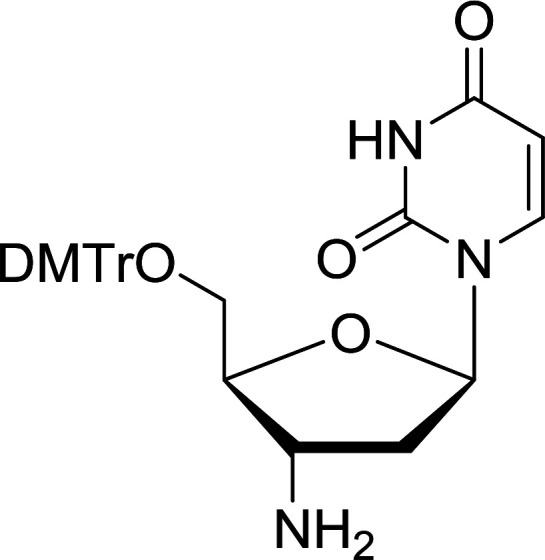	89
5	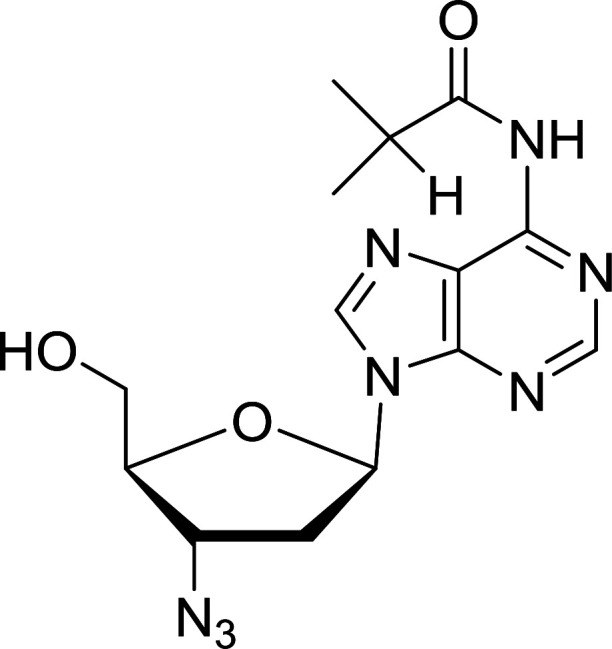	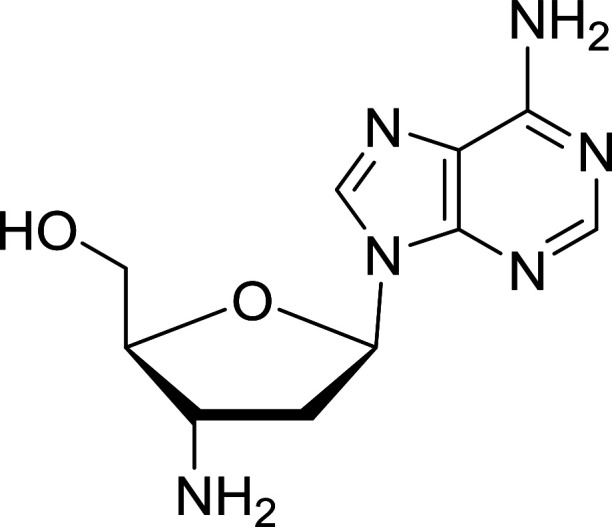	93
6	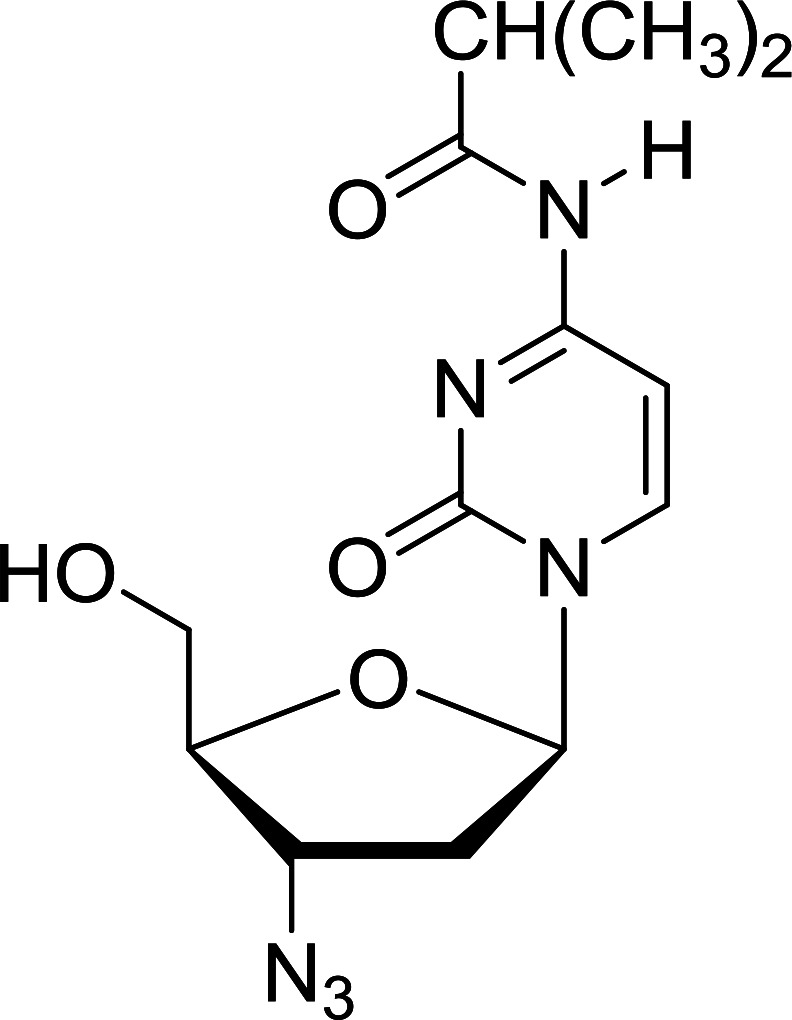	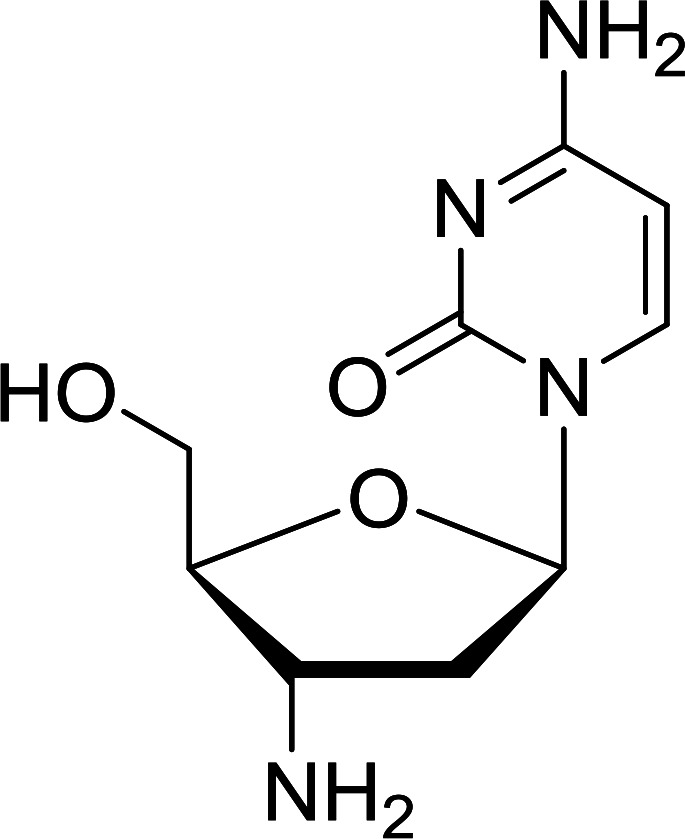	94

a Reagents and conditions: entries 1 and 2; 5 : 1 molar ratio of PS-TPP : azido nucleoside, dioxane, RT, 2 h, then H_2_O, 2 h; entry 4; molar ratio of PS-TPP : azido nucleoside, pyridine, RT, 3 h, then NH_3_ (32%), 5 h; entries 3, 5 and 6; 5 : 1 molar ratio of PS-TPP : azido nucleoside, dioxane, RT, 2 h, then NH_3_ (32%), overnight.

#### 3.4.2. Conversion of sugar azides to sugar isothiocyanates

Isothiocyanates are very useful synthetic intermediates in carbohydrate chemistry,^74^ and have been utilized in the synthesis of various functional groups as well as heterocyclic rings.^75^ Azides comprise convenient precursors as means to introduce the isothiocyanate group to sugars *via* a tandem Staudinger–aza-Wittig reaction of the azide with Ph_3_P and carbon disulfide.^75^ Fernández *et al.* demonstrated the utility of PS-TPP *in lieu* of Ph_3_P in the one-step preparation of non-anomeric sugar isothiocyanates from primary and secondary sugar azides (Table 23).^76^ While primary azidodeoxy sugars afforded the corresponding primary deoxyisothiocyanato in high yields (entries 1–3), secondary azides, especially those with the azide group located at an endocyclic carbon atom, gave much lower yields (entries 5 and 6). Mono- and disaccharides, including pyranose and furanose derivatives, protected with a variety of labile and sensitive *O*-protecting groups underwent successful functional group interconversion, illustrating the scope of the method. This strategy was not successful with glycosyl azides because of the higher reactivity of the anomeric isothiocyanate. Prior to the work of Fernández *et al.*, a similar reduction reaction of a 3′-azidonucleoside using PS-TPP was reported by Cech and Zehl as a technical improvement to the preparation of isothiocyanato derivatives.^77^ The proposed mechanism of these reactions involve initial formation of a polymer-supported iminophosphorane intermediate, followed by a cyclization reaction with carbon disulfide to give a four-membered ring, and subsequent cycloreversion of the ring to afford the isothiocyanate.

**Table 23:** Synthesis of deoxyisothiocyanato sugars from azidodeoxy sugars^a^

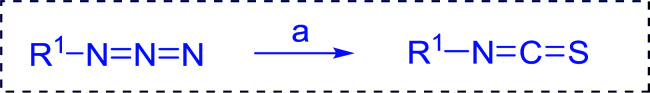			
Entry	Azide	Isothiocyanates	Yield (%)
1	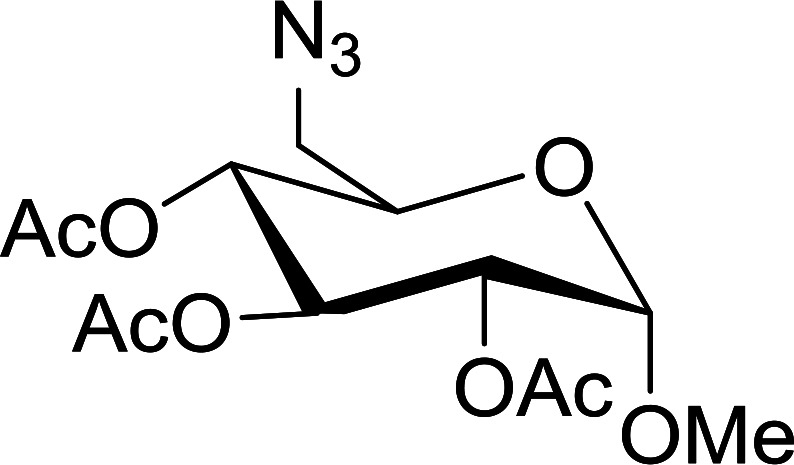	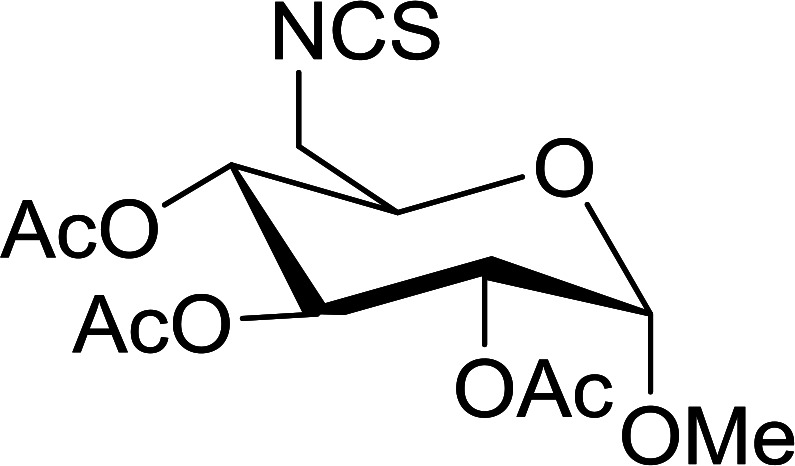	95
2	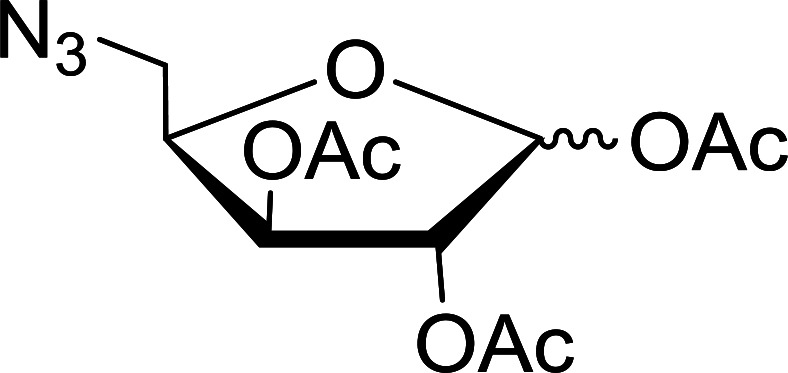	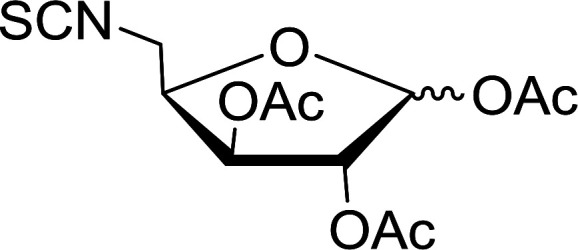	97
3	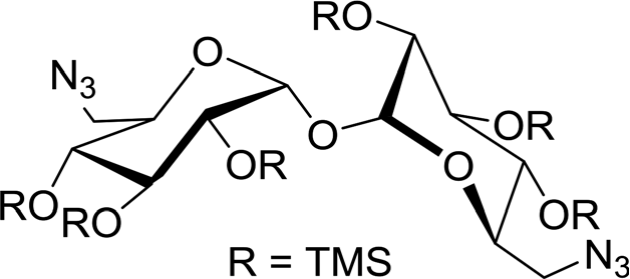	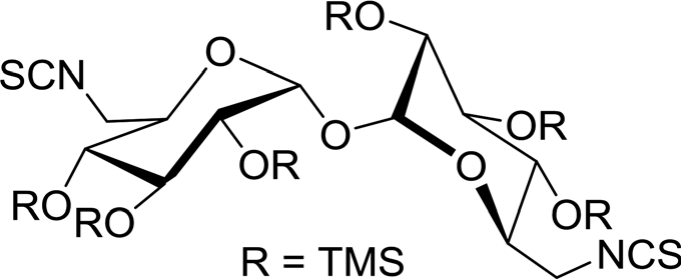	86
4	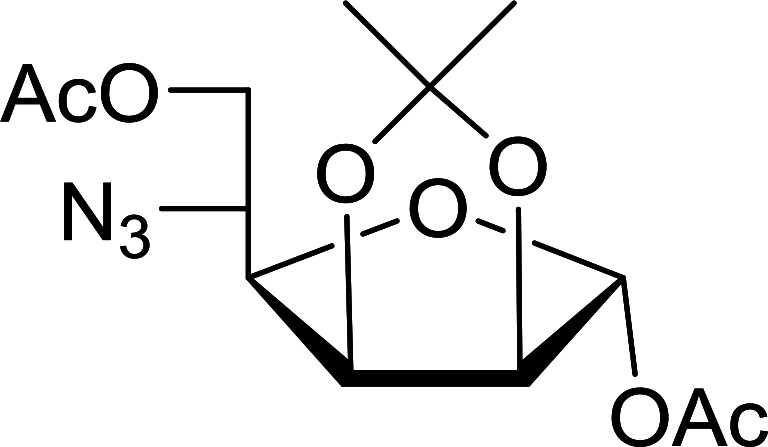	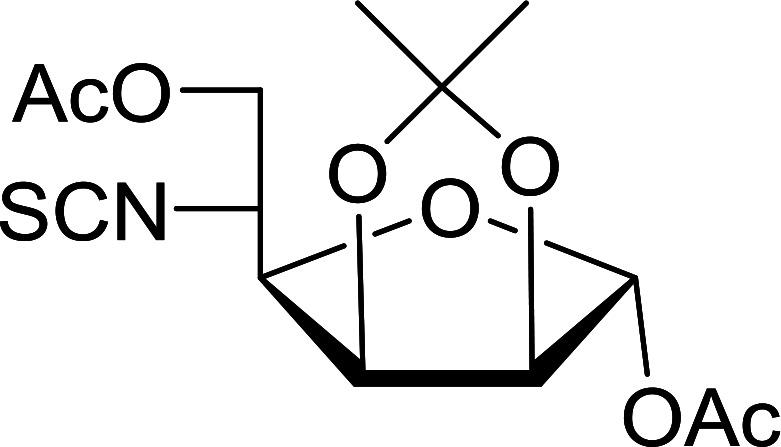	73
5	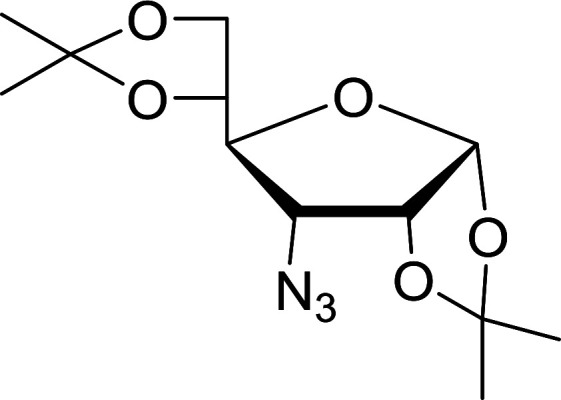	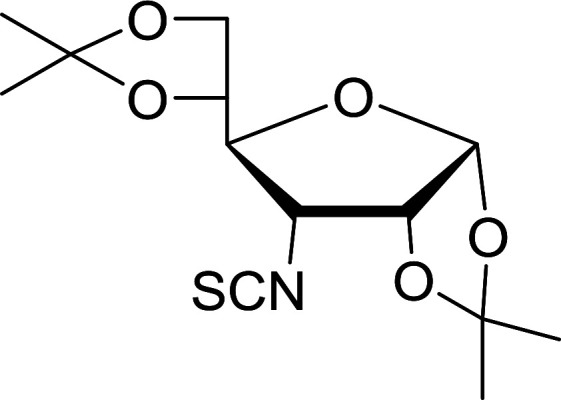	24
6	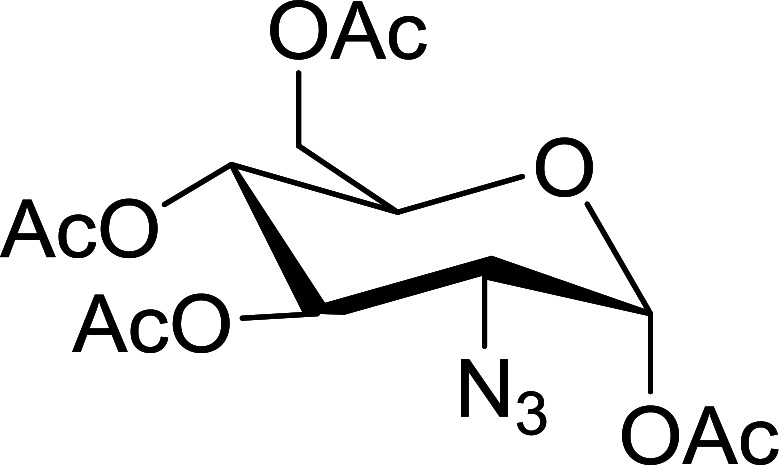	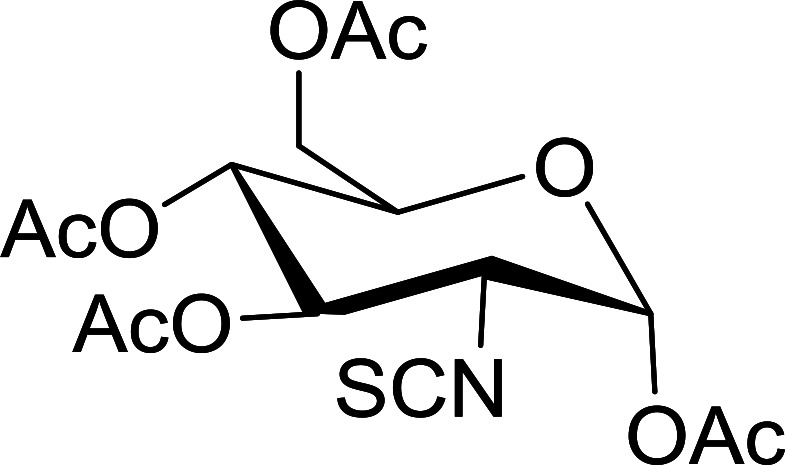	18

a Reagents and conditions: 1.2 : 1 : 10–25 molar ratios of PS-TPP : azidodeoxy sugars : CS_2_, dioxane, RT, 16–48 h.

#### 3.4.3. A one-pot aza-Wittig based polymer supported route to primary and secondary amines

Azides have been employed as the nitrogen source for the preparation of primary amines by converting the azide into an iminophosphorane (the Staudinger reaction), followed by hydrolysis.^78^ This approach, however, can not be used to access secondary amines because imine intermediates are not involved. Hemming and co-workers disclosed a study describing the conversion of azides 50 into α-unsubstituted primary amines 51 or α-branched secondary amines 52*via* the intermediacy of polymer-supported iminophosphoranes and imines (Table 24).^79^ Their strategy involved a Staudinger reaction between PS-TPP and various azides (R^1^-N_3_), affording polymer-supported iminophosphoranes. Subsequent *in situ* aza-Wittig reaction of the resulting iminophosphorane with aromatic or alkyl aldehydes (R^2^CHO) gave imine intermediates, which were either reduced *in situ* to the corresponding α-unsubstituted primary amines (path a), or functionalized *via* 1,2-addition of organometallic reagents (R^3^MgX or R^3^Li) to afford α-branched secondary amines (path b). The use of trimethylsilyl azides (R^1^ = TMS, entries 11–13) permitted synthesis of primary amines following *in situ N*-desilylation, whilst using benzyl azides allowed access to *N*-benzyl protected amines (entry 1, 6, 9 and 10). On the other hand, the wide range of suitable organometallic reagents allowed the preparation of synthetically useful homoallylic amines (entries 10 and 11). The yields ranged from 52–99% for most substrates except when the hindered pivalaldehyde was used (entry 6).

**Table 24:** Synthesis of primary and secondary amines from azides and aldehydes using PS-TPP^a^

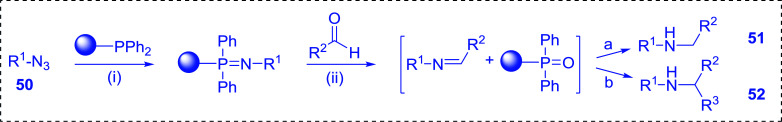				
Entry	R^1^	R^2^	R^3^	Yield (%)
1	Bn	Ph	H	99
2	Ph	*p*-F-C_6_H_4_	H	69
3	Ph	*n*-Pr	H	71
4	Ph	Me	H	52
5	*p*-MeO-C_6_H_4_	Ph	H	91
6	Bn	*t*-Bu	H	17
7	Ph	PhCH=CH	*n*-Bu	96
8	Ph	Ph	*n*-Bu	99
9	Bn	Ph	*n*-Bu	83
10	Bn	Ph	H_2_C=CHCH_2_	91
11	TMS	Ph	H_2_C=CHCH_2_	79
12	TMS	*p*-MeO-C_6_H_4_	*n*-Bu	69
13	TMS	Ph	Ph	59

a Reagents and conditions: (i) 4 : 1 molar ratio of PS-TPP : azide, THF, RT, 2–3 h. (ii) Aldehyde (1.5 molar equivalent), THF, reflux, 3 h; entries 1–6, path a, NaBH_3_CN or NaBH_4_ or BH_3_-THF (1.5 molar equivalent), MeOH, NH_4_Cl (aq), RT, 1 h; entries 7–13, path b, R^3^MgX or RLi, NH_4_Cl (aq), RT, 1 h.

#### 3.4.4. Aza Wittig synthesis of pyrido[1,2-*c*]pyrimidine heterocycles

**Scheme 13:**
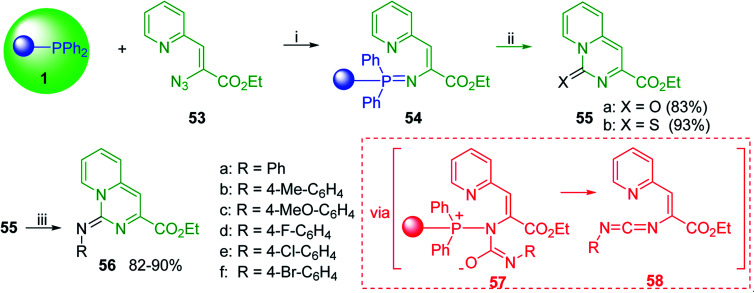
Preparation of pyrido[1,2-*c*]pyrimidines. Reagents and conditions: (i) CH_2_Cl_2_, RT; (ii) solid CO_2_ or CS_2_, toluene, sealed tube, 90 °C; (iii) Ar-NCO, CH_2_Cl_2_, RT.

Pyrido[1,2-*c*] pyrimidines comprise a promising class of heterocycles which may potentially provide novel superoxide scavengers and anti-inflammatory agents.^80^ Molina and co-workers designed a solid-phase synthesis of these fused azaheterocycles *via* an aza Wittig reaction under mild reaction conditions. Their strategy for the synthesis of pyrido[1,2-*c*] pyrimidines 55 and 56 is outlined in Scheme 13. Staudinger reaction between PS-TPP (1) and ethyl α-azido-β-(2-pyridyl)acrylate (53) in dry CH_2_Cl_2_ at room temperature gave the polymer-bound iminophosphorane 54. Subsequent reaction of 54 with carbon dioxide or carbon disulfide at 90 °C afforded pyrido[1,2-*c*]pyrimidines 55a and 55b in 83% and 93% yield, respectively. On the other hand, aza Wittig reaction of iminophosphorane 54 with aromatic isocyanates produced the carbodiimides 58 through the intermediacy of species 57. The former (58) underwent regioselective electrocyclization to give the corresponding pyrido[1,2-*c*]pyrimidines 56 in yields ranging from 82% to 90%.

#### 3.4.5. Synthesis of secondary amines from alkyl azides and alkyl halides

The most popular methods for the synthesis of secondary amines use either reductive aminations or alkylation procedures on primary amines. However, overalkylation leading to the formation of the corresponding tertiary or quaternary ammonium salts is difficult to avoid. Classon *et al.* described a one-pot, two-step procedure that delivers only secondary amines from the corresponding azides and reactive alkyl halides using polymer-bound, triphenylphosphine-supported synthesis (Table 25).^81^ The solid-phase method involves a Staudinger reaction between PS-TPP (1) and the azide to afford the corresponding phosphoazide which rapidly eliminates nitrogen to give an iminophosphorane intermediate. Subsequent alkylation with alkyl (entries 1 and 6), allyl (entry 4), or benzyl (entries, 2, 3, 5 and 7) halides produced the corresponding resin-bound disubstituted aminophosphonium salts. Finally, the secondary amines were freed from the solid support by hydrolytic cleavage using methanolic KOH. The yields obtained were good in all examples (78–87%) except when phenylazide was used (21%) (entry 5). The low yield was attributed to its reduction to the corresponding aniline.

**Table 25:** Synthesis of secondary amines^a^

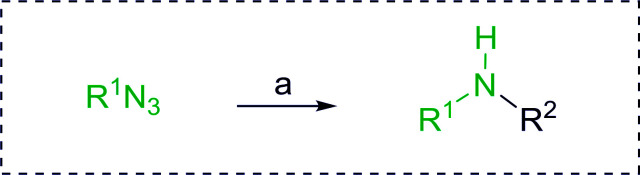				
Entry	Azide	Alkyl halide	Product	Yield (%)
1	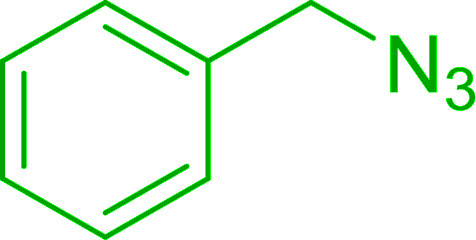	MeI	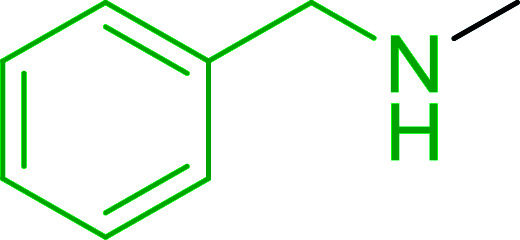	81
2	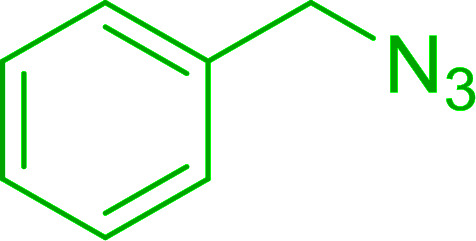	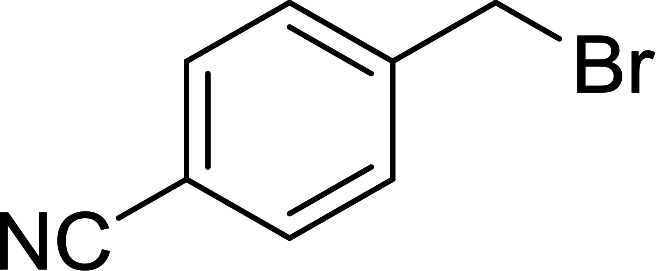	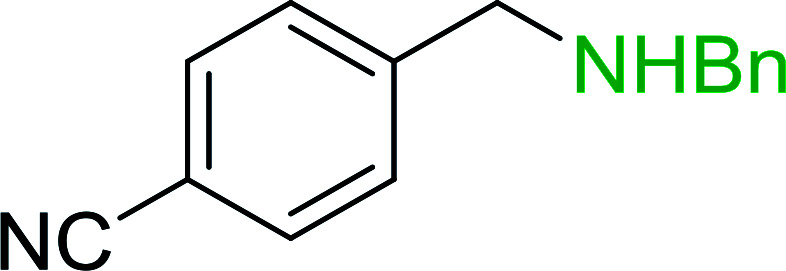	82
3	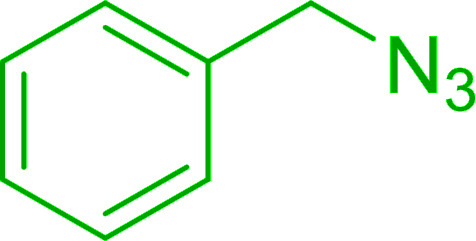	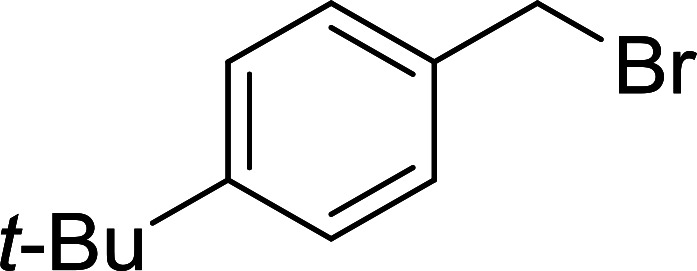	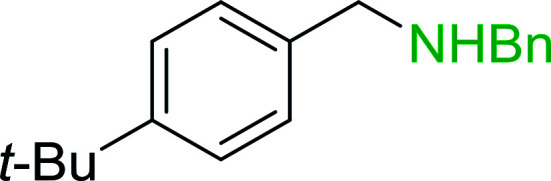	87
4	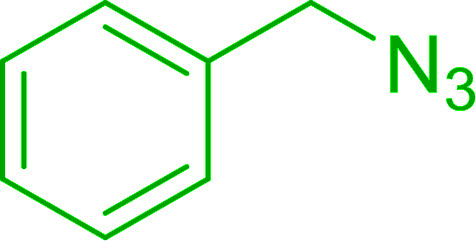	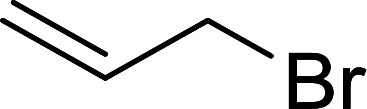	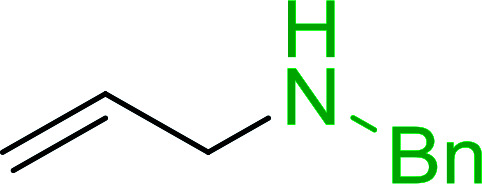	78
5	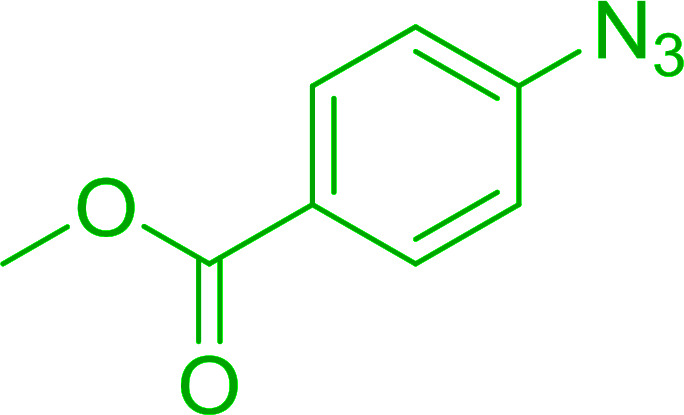	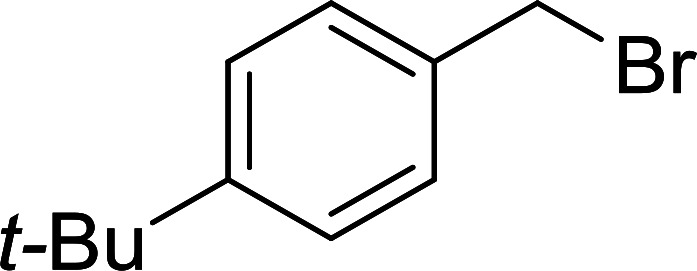	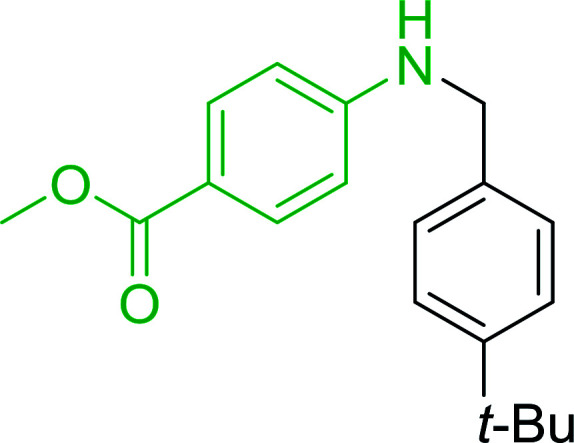	21
6	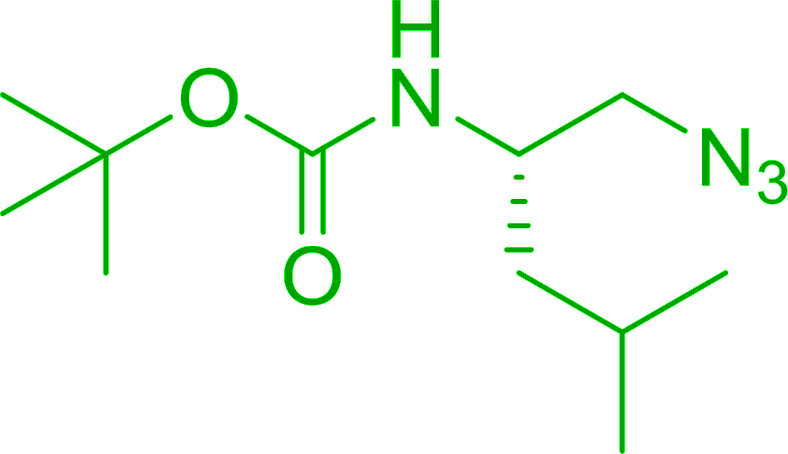	MeI	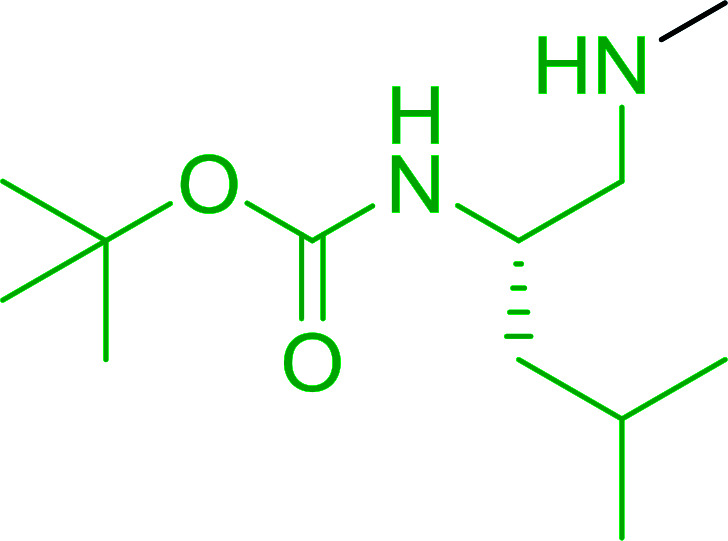	85
7	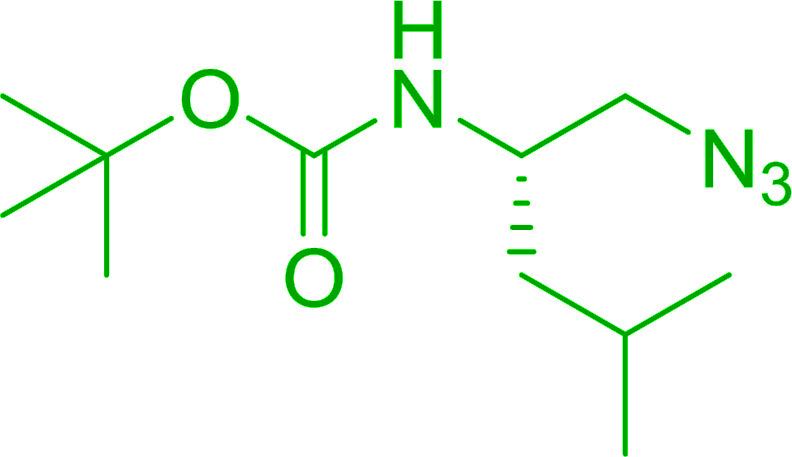	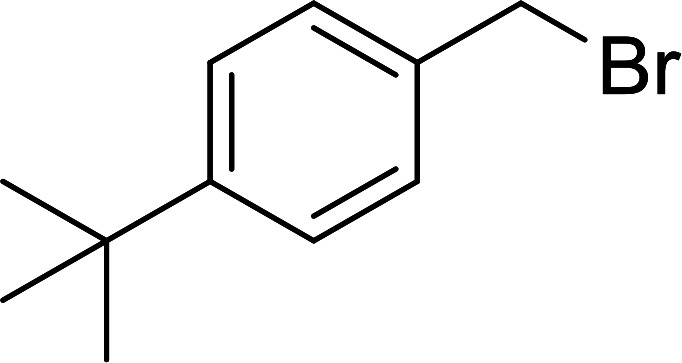	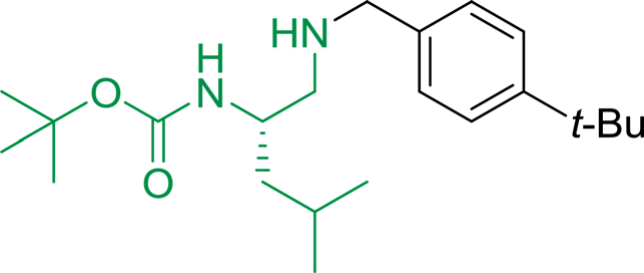	82

a Reagents and conditions: (i) PS-TPP, THF, 5 min. RT.; (ii) azide, THF, 4 h, RT.; (iii) alkyl halide, THF, 16 h, RT; (iv) KOH–MeOH, 4 h, 65 °C; 2 : 1 : 3 : 10 molar ratios of PS-TPP : azide : alkyl halide : KOH–MeOH.

#### 3.4.6. Synthesis of glucopyranosyl amides using PS-TPP

**Scheme 14:**
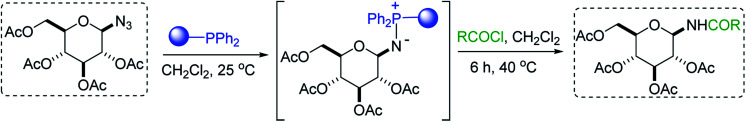
Synthetic strategy towards the synthesis of glucopyranosyl amides using PS-TPP. Reagents and conditions: 1.3 : 1 : 2 molar ratios of PS-TPP : azide : acid chloride, CH_2_Cl_2_, 40 °C, 6 h.

The presence of the glycosyl amide motif in naturally occurring biomolecules like glycoproteins and nucleic acids has inspired interest in the synthesis of compounds containing such structural feature.^82^ In particular, glycosyl amides have been synthetically targeted because they have been suggested as potential inhibitors of glycosyl hydrolases^83^ and the binding of fibroblast growth factor (FGF-2) to heparin.^84^ Norris *et al.* devised a method for the introduction of the glycosyl amide by way of a Staudinger process in which a glycosyl azide reacts with PS-TPP, followed by reaction of the resulting iminophosphorane with an acid chloride (Scheme 14).^85^

Norris *et al.* reacted 2,3,4,6-tetra-*O*-acetyl-β-d-glucopyranosyl azide with PS-TPP and various acid chlorides (Table 26, entries 1–9) using a parallel synthesizer to prepare a small library of glucopyranosyl amides. The excess acid chloride was removed at the end of the reaction by treatment with polymer-supported tris(2-aminoethyl) amine. Optimum yield was observed with *p*-nitrobenzoyl chloride, underscoring the preference of the polymer-bound iminophosphorane for highly electrophilic acid chlorides (entry 1). On the other hand, the highly hindered pivaloyl chloride inhibited the reaction between the iminophosphorane intermediate and acid chloride (entry 8).

**Table 26:** Synthesis of glucopyranosyl amides from carboxylic acids

Entry	Acid chloride	Azide	Glucopyranosyl amide yield (%)
1	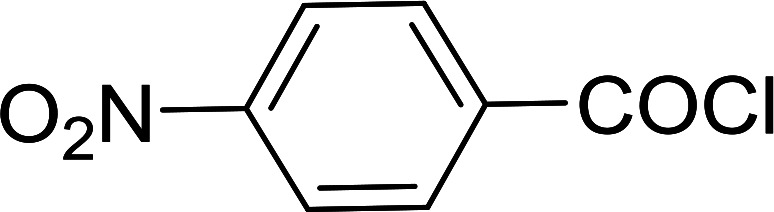	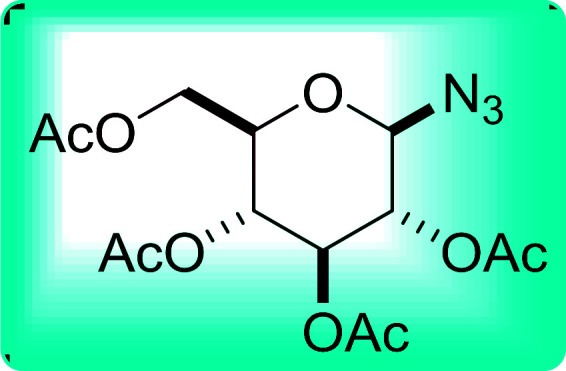	93
2	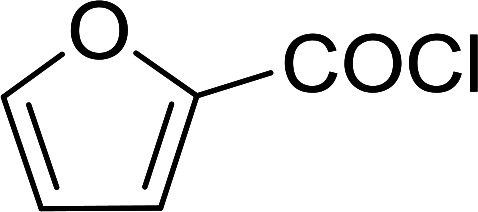		40
3	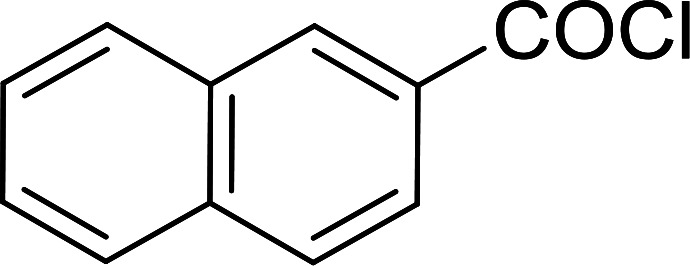		72
4	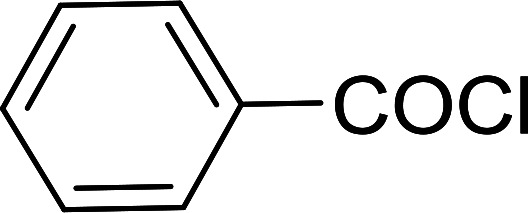		55
5	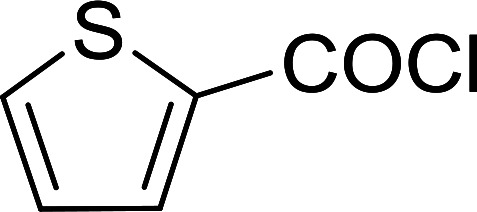		56
6	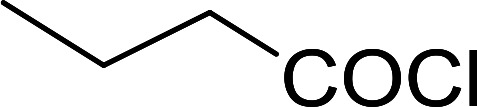		61
7	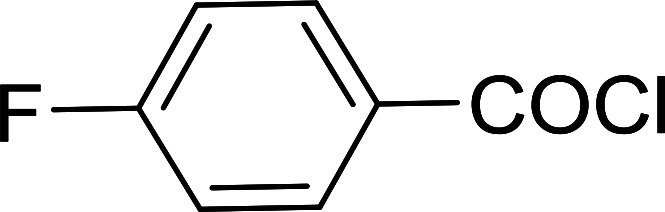		61
8	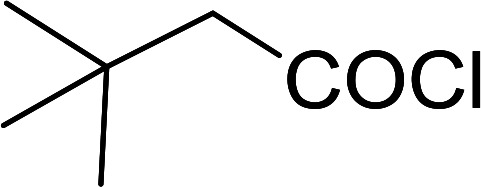		—
9	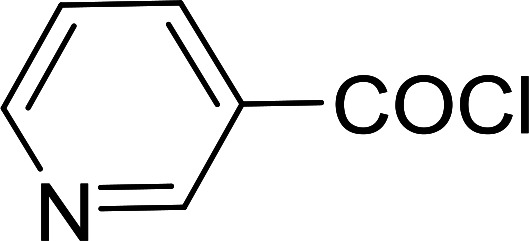		40

### 3.5. The Mitsunobu reaction

#### 3.5.1. Esterification using PS-TPP

The condensation of alcohols with carboxylic acids *via* triphenylphosphine-azodicarboxylate activation is one of the most reliable and common methods for esterification (the Mitsunobu reaction).^86^ However, byproducts from the Mitsunobu reaction and excess reagents are non-volatile and soluble in organic solvents necessitating difficult chromatographic separation of the desired product almost always. PS-TPP was first utilized in the Mitsunobu reaction by Amos *et al.* in 1983 for the preparation of esters from alcohols and carboxylic acids (Table 27).^87^ The use of PS-TPP conferred convenience in terms of purification of the ester product. The method worked well with aliphatic and aromatic acids and tolerated a variety of functional groups as shown in Table 27. The yield was unaffected in the series of benzoic acids substituted with electron-donating and electron-withdrawing groups (entries 1 and 4–7). As anticipated, secondary alcohols (entries 3, 4 and 11) gave consistently lower yields than primary alcohols (entries 1, 2 and 5–10) and tertiary alcohols were too hindered and remained unreacted. When an optically pure alcohol was used (entry 11), inversion of the chiral center was observed.

**Table 27:** Preparation of esters from alcohols and carboxylic acids using PS-TPP^a^

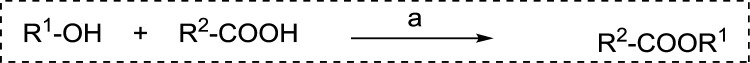				
Entry	Alcohol	Carboxylic acid	Ester product	Yield (%)
1	PhCH_2_OH	PhCO_2_H	PhCO_2_Bn	86
2	PhCH_2_OH	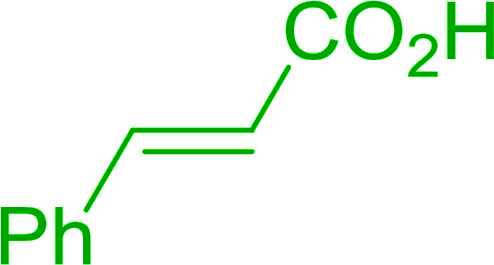	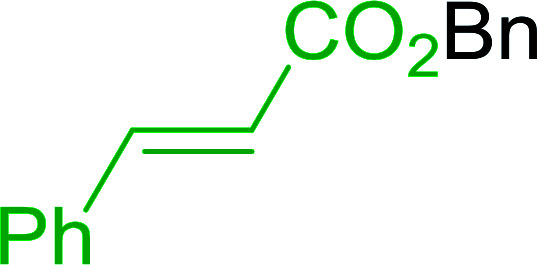	86
3	C_2_H_5_CH(CH_3_)OH	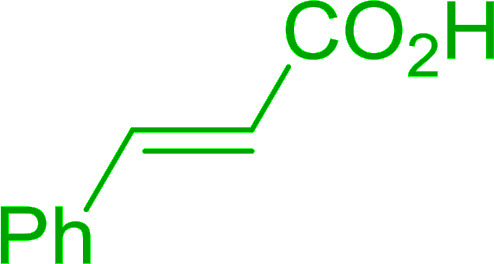	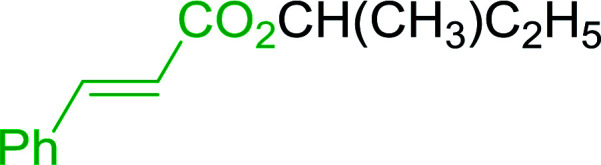	64
4	Cyclopentanol	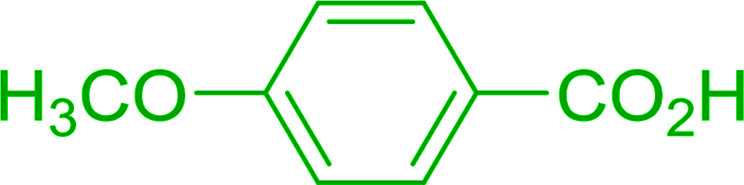	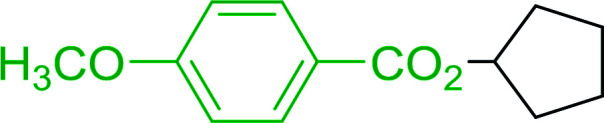	75
5	1-Heptanol	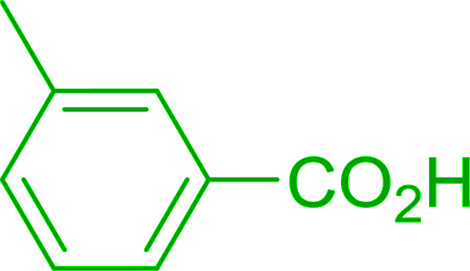	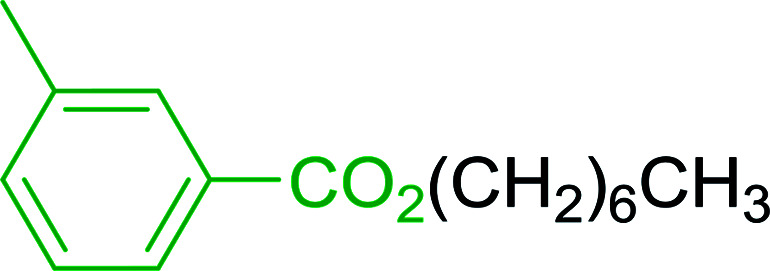	90
6	1-Heptanol	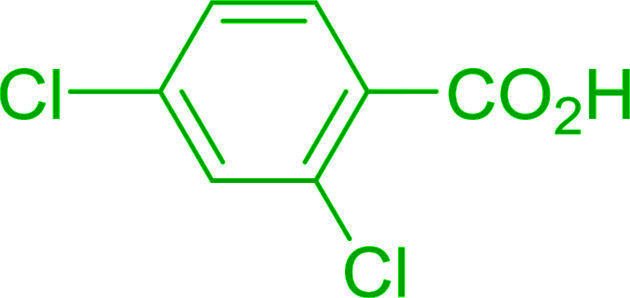	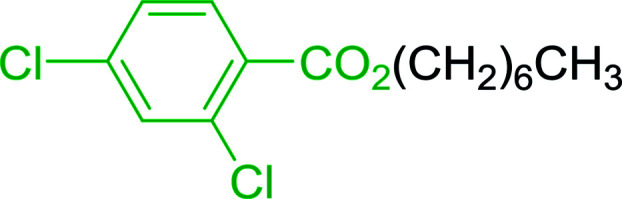	89
7	1-Heptanol	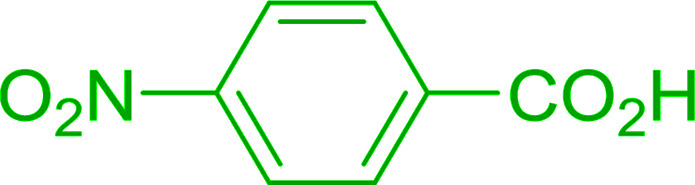	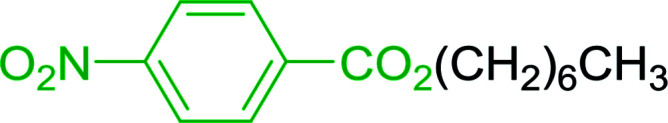	84
8	PhCH_2_OH	(CH_3_)CCO_2_H	(CH_3_)CCO_2_Bn	99
9	EtOH	HO_2_C(CH_2_)_4_CO_2_H	EtO_2_C(CH_2_)_4_CO_2_Et	79
10	1-Heptanol	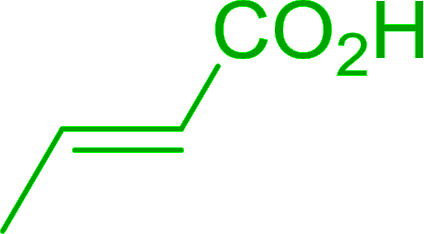	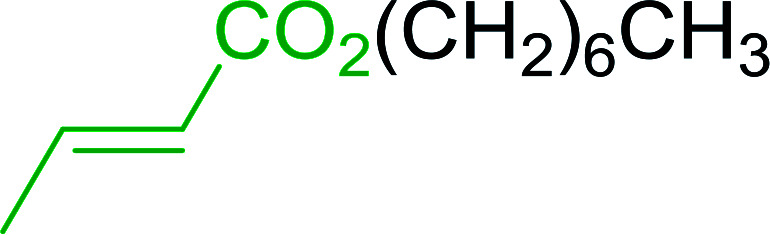	92
11	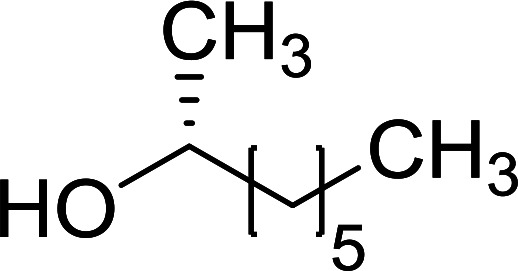	PhCO_2_H	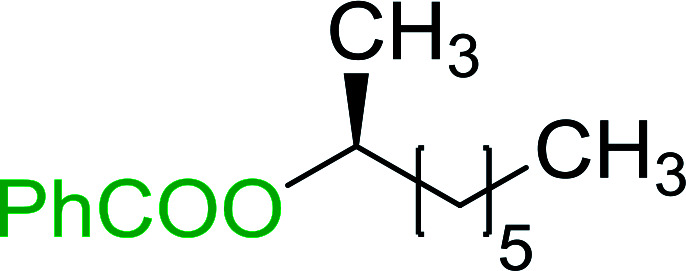	65

a Reagents and conditions: 1.8 : 1.4 : 1 : 1 molar ratios of PS-TPP : DEAD : carboxylic acid : alcohol, THF, 25 °C, 4 h.

#### 3.5.2. Synthesis of aryl ethers from phenols and alcohols

Georg and co-workers^88^ reported the first example of a Mitsunobu reaction^86^ using PS-TPP in the synthesis of aryl ethers. The group prepared a library of aryl ethers from phenols with electron releasing and electron withdrawing groups and various alcohols (Table 28).^88^ In a typical experiment, PS-TPP, diethyl azodicarboxylate (DEAD), the alcohol, and the phenol in dichloromethane were stirred at room temperature for 4–12 hours. Filtration of the resin, followed by evaporation of the solvent *in vacuo* and purification by column chromatography afforded pure aryl ethers in 59–94% yields. It is noted that presence of electron withdrawing substituents at the aromatic ring of the phenol (Cl and CN) accelerated the reaction rate, whereas electron donating substituent (OMe) generally reduced the reaction rate, resulting in a slower reaction progress.

**Table 28:** Preparation of aryl ethers from alcohols and phenols using PS-TPP^a^

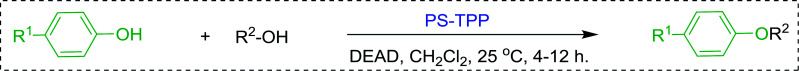					
Entry	Phenol	Alcohol	Aryl ether	Product	Yield (%)
1	R^1^ = CN	R^2^ = heptyl	R^1^ = CN	R^2^ = heptyl	68
2	R^1^ = CN	R^2^ = benzyl	R^1^ = CN	R^2^ = benzyl	94
3	R^1^ = CN	R^2^ = furfuryl	R^1^ = CN	R^2^ = furfuryl	88
4	R^1^ = Cl	R^2^ = heptyl	R^1^ = Cl	R^2^ = heptyl	65
5	R^1^ = Cl	R^2^ = benzyl	R^1^ = Cl	R^2^ = benzyl	88
6	R^1^ = Cl	R^2^ = furfuryl	R^1^ = Cl	R^2^ = furfuryl	84
7	R^1^ = OMe	R^2^ = heptyl	R^1^ = OMe	R^2^ = heptyl	63
8	R^1^ = OMe	R^2^ = benzyl	R^1^ = OMe	R^2^ = benzyl	86
9	R^1^ = OMe	R^2^ = furfuryl	R^1^ = OMe	R^2^ = furfuryl	78

a Reagents and conditions: 1.5 : 1.5 : 1.5 : 1 molar ratios of PS-TPP : DEAD : alcohol : phenol.

##### 3.5.2.1. Stereochemical inversion of secondary alcohols

Georg *et al.* found that polymer-supported triphenylphosphine can replace triphenylphosphine in the Mitsunobu reaction to form stereochemically inverted secondary alcohols.^89^ The procedure addresses the most significant drawback associated with the Mitsunobu reaction which includes removal of excess triphenylphosphine and its oxide by-product. The protocol is very comparable to the standard Mitsunobu reaction in regards to yield, stereochemical inversion, reaction time, and even inversion of sterically hindered secondary alcohols. As shown in Table 29, benzylic, alkyl, and cyclic secondary alcohols underwent successful inversion of configuration, although yields were slightly lower with the former two types of substrates (entries 3 and 5) and much lower for sterically hindered ones (entry 6). It is noted that no racemisation was observed for substrates bearing an acidic proton at the stereocenter (entries 2 and 4). The yields reported were in general comparable to those obtained when the inverted products were prepared using standard homogeneous Mitsunobu conditions, suggesting that the reactivity of PS-TPP is similar to its free form. Many of the inverted esters were hydrolyzed to the corresponding alcohols with LiOH which were isolated in 95–97% yields. Optical rotation analysis of the alcohols showed complete inversion of configuration when compared with the starting material.

**Table 29:** Stereochemical inversion of secondary alcohols^a^^,^^b^

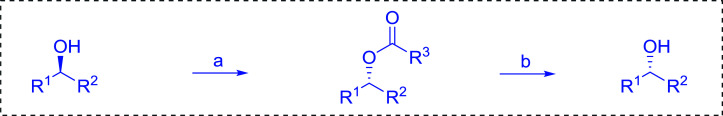			
Entry	Alcohol	Inverted ester	Yield^c^ (%)
1	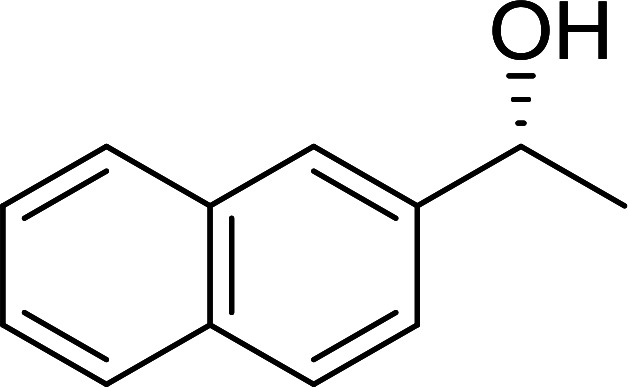	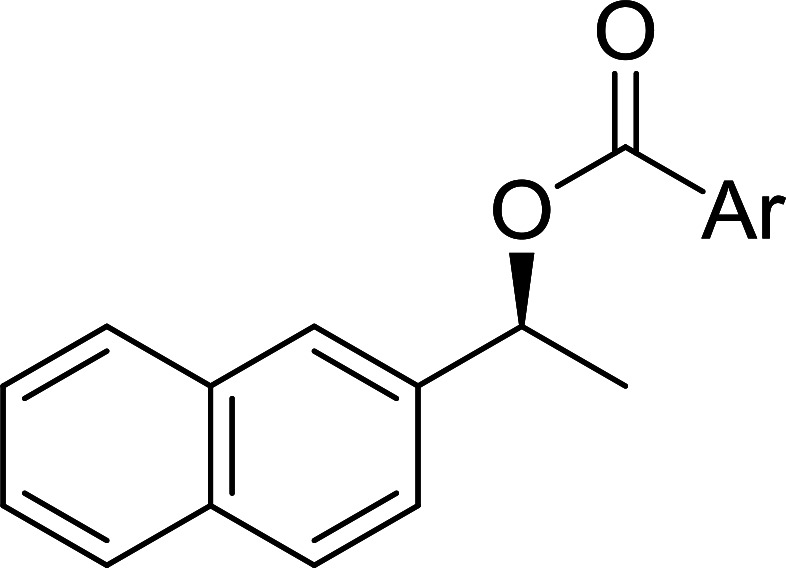	98
2	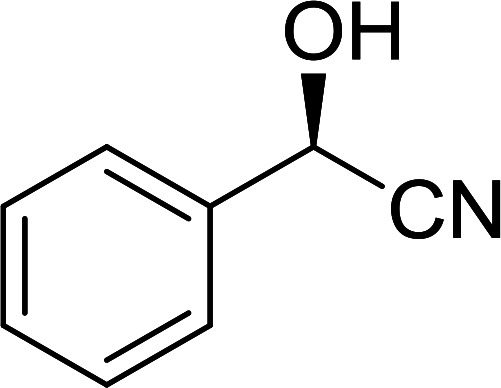	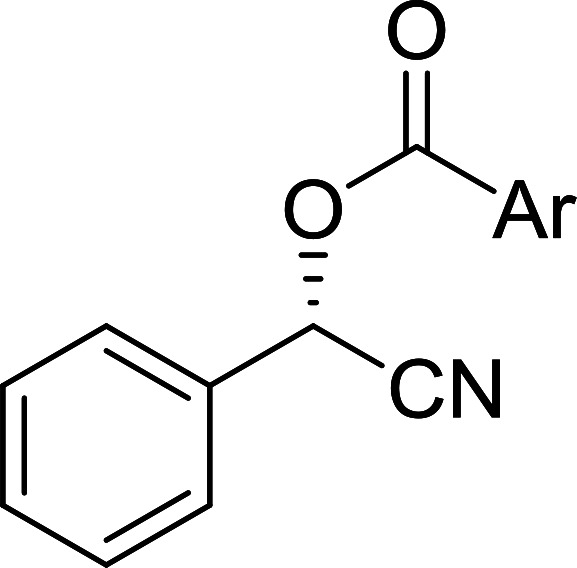	74
3	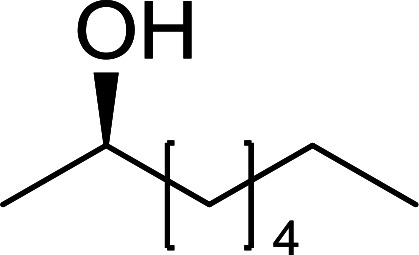	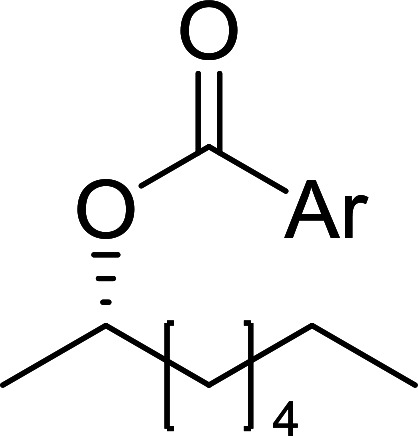	65
4	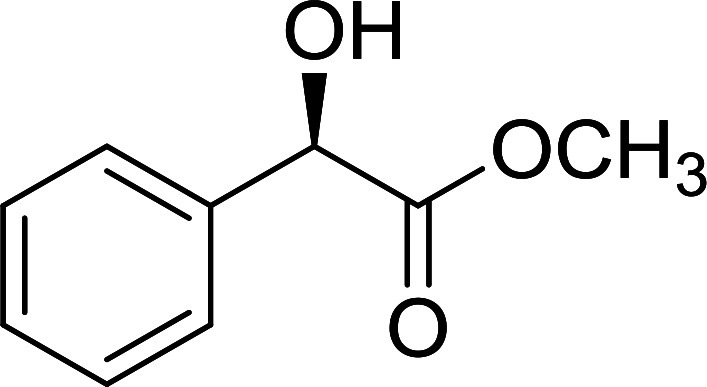	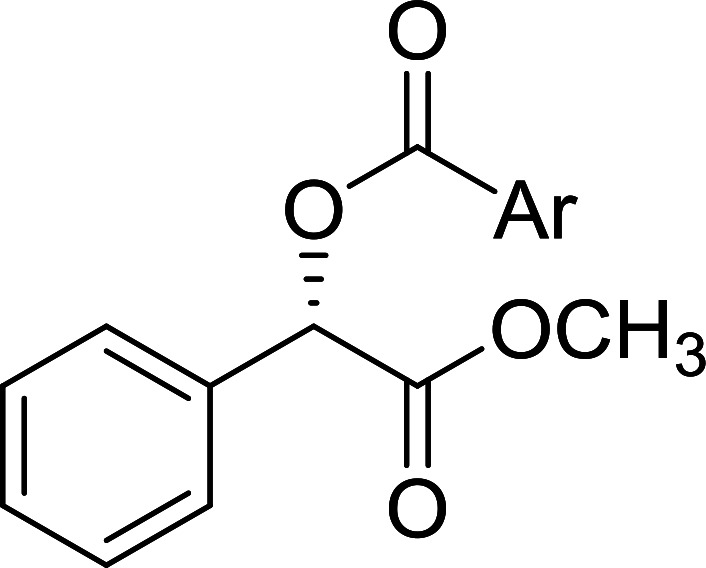	76
5	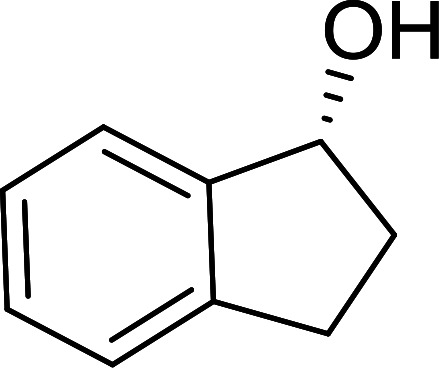	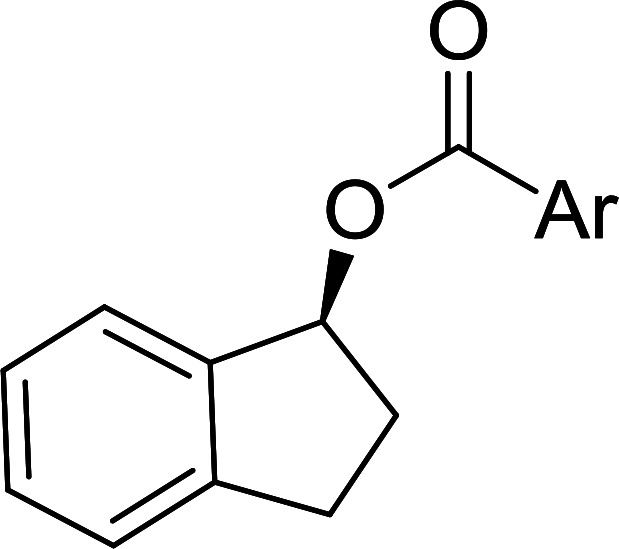	71
6	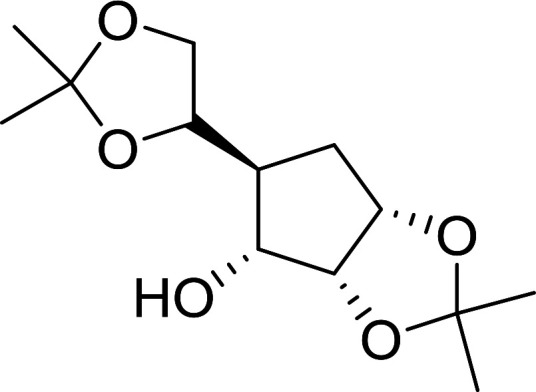	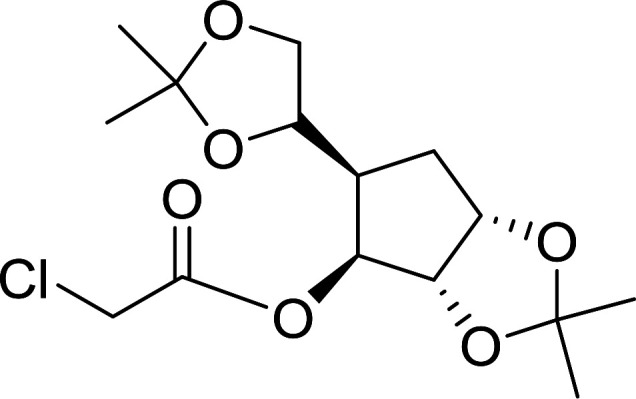	25

a Reagents and conditions: 1 : 1.2 : 2 : 2 molar ratios of alcohol : R^3^COOH (R^3^COOH = 4-nitrobenzoic acid or chloroacetic acid) : DEAD : PS-TPP, THF, RT, 3–12 h.

b Reagents and conditions: LiOH, H_2_O/acetone, RT, 95–97% yield.

c The yield shown is for step a.

#### 3.5.3. Synthesis of aryl ethers from aminoalcohols

Building on the method of Georg *et al.* who described earlier the preparation of aryl–alkyl ethers from phenols and alcohols using a solution-based Mitsunobu coupling strategy with PS-TPP,^88^ Shuttleworth *et al.* discovered an improvement to the etherification reaction and reported optimum conditions.^90^ Thus, when Shuttleworth and co-workers treated a collection of phenols with *N*-protected aminoalcohols under Georg's conditions, poor conversion to the ether products was observed (16–49% yields). It was shown that the progress of this reaction could be significantly enhanced when a tertiary amine base is used and the order of reagent addition is modified. The improved procedure calls for the treatment of a slight excess of PS-TPP with DEAD at 0 °C for 5 min, followed by the addition a premixed solution of the aminoalcohol, phenol, and Et_3_N. It was suggested that the base enhances the overall rate of reaction as it plays a role in the formation of the phenolate required for the S_N_2 nucleophilic displacement of the alkoxyphosphonium salt derived from the aminoalcohols. The modified approach was successfully applied to the synthesis of a range of aryl ethers from phenols and *N*-protected aminoalcohols (Table 30).

**Table 30:** Preparation of aryl ethers from aminoalcohols

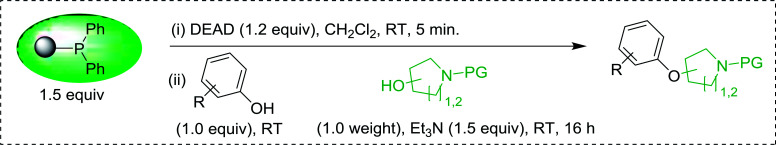					
Entry	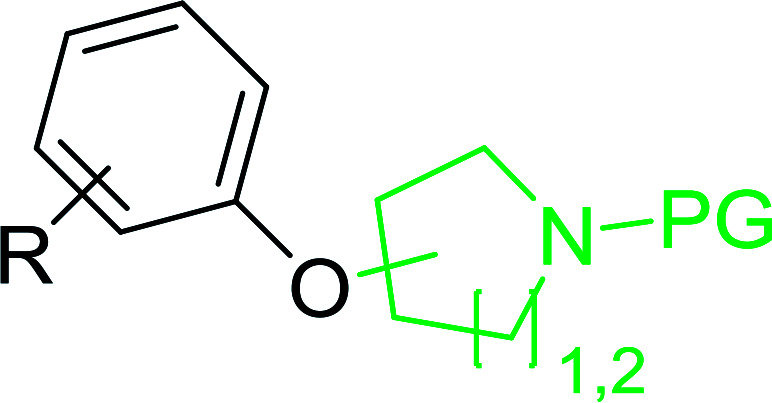	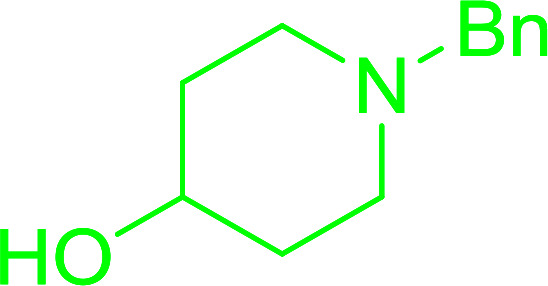	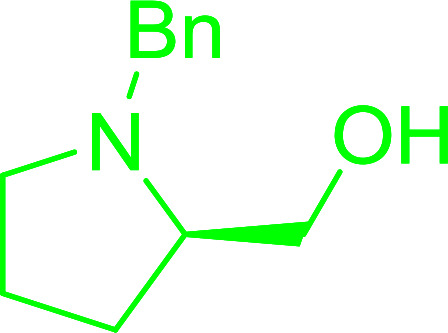	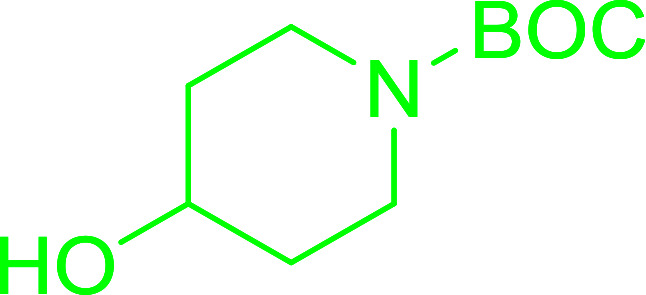	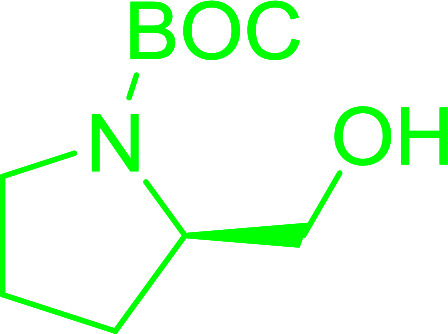
1	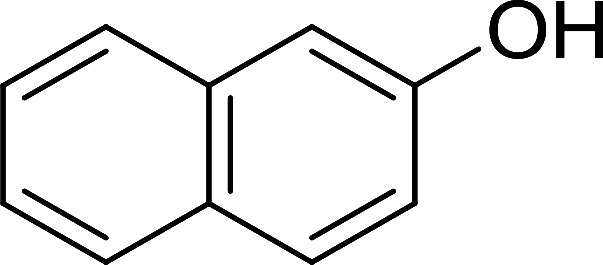	72% conv.	68% conv.	61% conv.	75% conv.
2	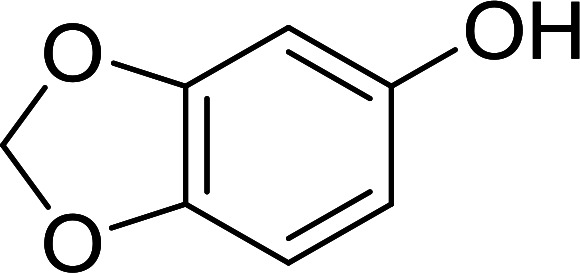	64% conv.	98% conv.	75% conv.	99% conv.

#### 3.5.4. Scavenging the hydrazinedicarboxylate ester byproduct in the Mitsunobu reaction with PS-TPP (solid phase scavenger)

**Scheme 15:**
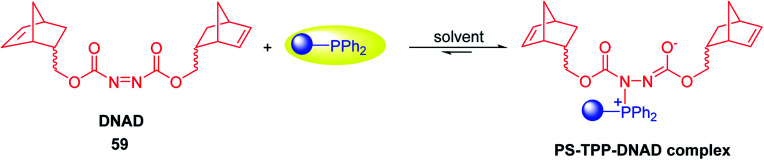
Removal of excess DNAD from the Mitsunobu reaction with PS-TPP.

The Mitsunobu reaction is a four component process that utilizes an alcohol, nucleophile, phosphine, and azodicarboxylate ester. Furthermore, upon reaction completion, two side products, the hydrazinedicarboxylate ester and phosphine oxide, are generated. In their quest for impurity annihilation and to add further improvement to the Mitsunobu process, Barrett *et al.* employed PS-TPP (1) and an olefinic azodicarboxylate (bis(5-norbornenyl-2-methyl) azodicarboxylate, DNAD, 59) to obtain Mitsunobu products in 43–100% yields (86–96% purity) from alcohols and carboxylic acids or their nucleophilic equivalents (phthalimides, or *N*-hydroxyphthalimides).^91^ Ring opening metathesis-polymerization (ROMP) of the hydrazine by-product (DNADH_2_) using Grubbs catalyst (Cl_2_(Cy_3_P)_2_Ru=CHPh) converted it to a poly(DNADH_2_) solid which was conveniently removed by filtration alongside PS-TPPO. In addition to its role in the Mitsunobu reaction, PS-TPP was added during reaction work-up to form a complex with excess DNAD so it may be filtered out (Scheme 15).

#### 3.5.5. The Mitsunobu reaction with PS-TPP and di-*t*-butylazodicarboxylate

**Scheme 16:**
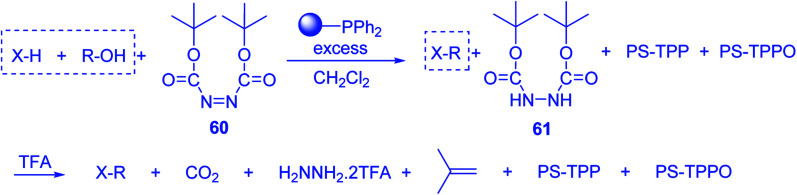
Mitsunobu reaction with PS-TPP and removal of byproducts.

Others groups have also described alternative methods to improved the Mitsunobu process by using PS-TPP with azodicarboxylates that can be destroyed *in situ*. Pelletier *et al.* reported a protocol in which PS-TPP and commercially available di-*t*-butylazodicarboxylate (DBAD, 60) were used in the Mitsunobu reaction whereby the latter and its expected hydrazide byproduct 61 were destroyed *in situ* upon treatment with trifluoroacetic acid (TFA) at the completion of the reaction (Scheme 16).^92^ Both of these compounds provide volatile gaseous byproducts (2-methylpropene) and water-soluble hydrazine ditrifluoroacetate. Filtration to remove the phosphine oxide resin and excess PS-TPP followed by standard aqueous work-up afforded products in high purity (>95%) in certain cases. This approach was employed to prepare a 3 × 5 parallel library which was free of phosphine and hydrazine impurities without recourse to chromatography. The methodology was successfully applied to the alkylation of a wide range of nucleophiles containing many representative and moderately acidic functional groups (sulphonamide, phenol, imide, hydantoin, and carboxylic acid) with a variety of alcohols (methanol, isopropanol, benzyl alcohol). A similar protocol using TFA for the destruction and removal of DBAD was used by Aberle *et al.* during PS-TPP-mediated esterification and inversion of carbinol stereochemistry of a tropane alkaloid.^93^

### 3.6. Conversion of carboxylic acids to amides

Buchstaller *et al.* reported a two-step procedure for the conversion of carboxylic acids into amides at ambient temperature under neutral conditions.^94^ Initially, the acid was converted into the acid chloride using PS-TPP and trichloroacetonitrile. The reaction was carried out in dichloromethane at room temperature for 3 h. Subsequent treatment with various types of amines (aromatic, benzylic, primary, and secondary) and polymer-bound morpholine as a base afforded the desired amides in 42–99% yields (Table 31).

**Table 31:** Synthesis of various amides from carboxylic acids^a^

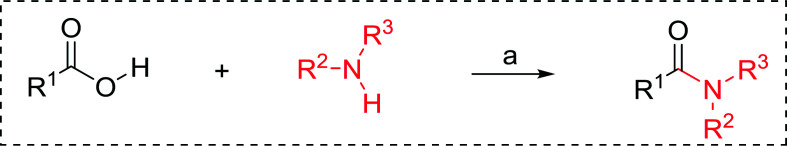						
Entry	Product					
	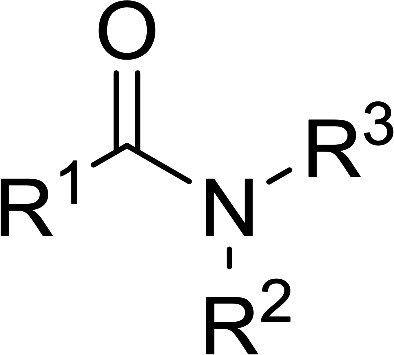	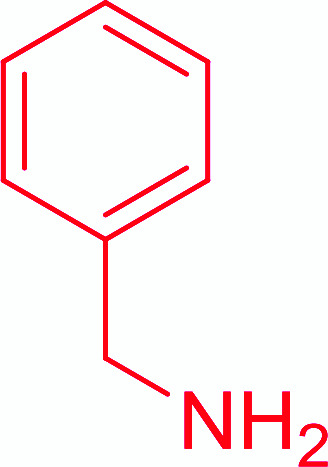	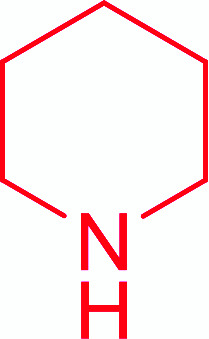	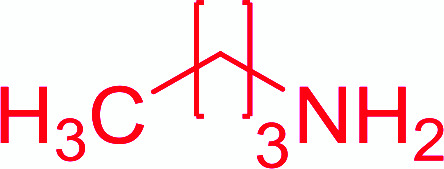	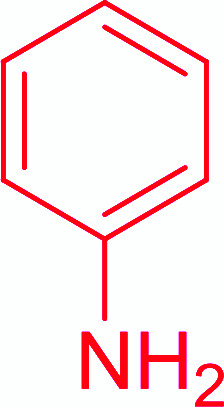	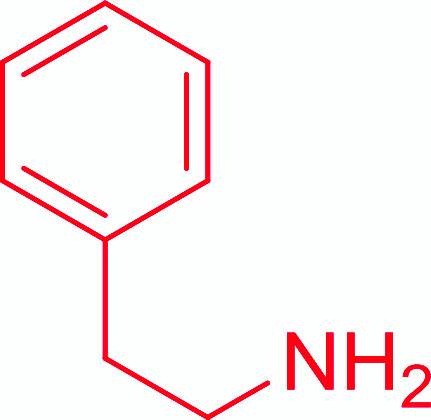
1	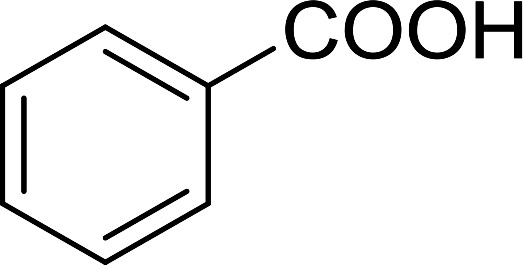	73	93	89	75	82
2	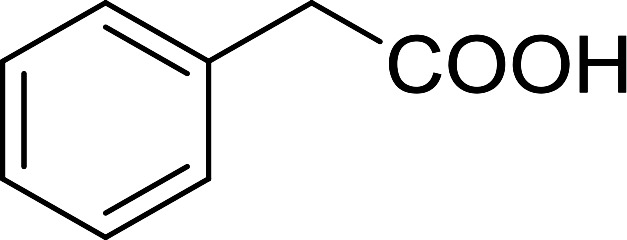	77	96	95	86	73
3	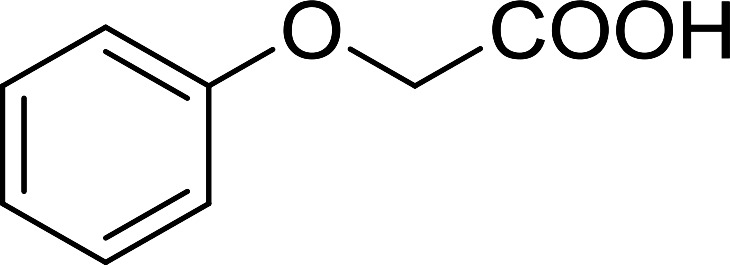	86	99	97	86	86
4	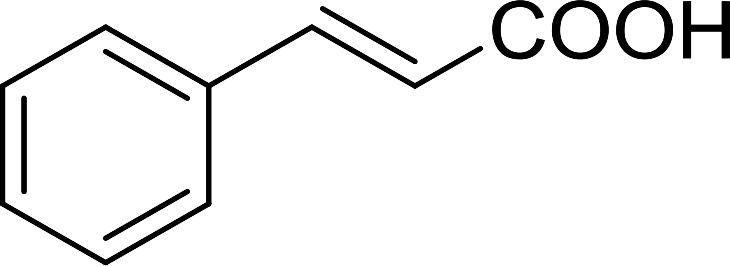	89	99	98	83	86
5	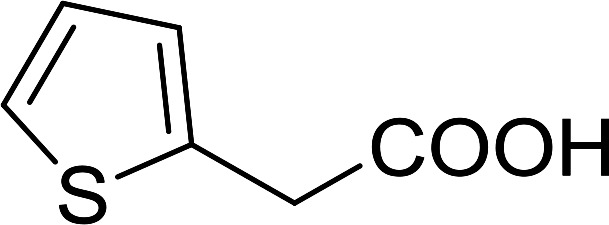	71	87	99	73	83
6	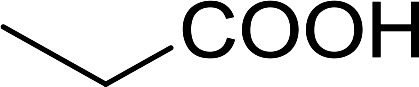	70	42	71	66	76

a Reagents and conditions: (i) PS-PPh_3_, CCl_3_CN, carboxylic acid, CH_2_Cl_2_, 3 h, RT.; (ii) amine, polymer-bound morpholine, CH_2_Cl_2_, 4 h, RT; 3 : 5 : 1 : 1 : 3 molar ratios of PS-TPP : CCl_3_CN : carboxylic acid : amine : polymer-bound morpholine.

### 3.7. Ultrasound-mediated esterification of carboxylic acids catalyzed by polymer-supported triphenylphosphine

Very recently, Pattarawarapan *et al.* described a sonochemical method for the methyl esterification of carboxylic acids catalyzed by polymer-supported triphenylphosphine.^95^ Thus, using 1 : 2 : 0.1 molar ratio of 2,4,6-trichloro-1,3,5-triazine (TCT)/Na_2_CO_3_/PS-Ph_3_P, various carboxylic acids containing reactive hydroxyl groups as well as acid- and base-sensitive moieties were converted to methyl esters in one step without the need to pre-activate the acid (Table 32). The products were obtained in 70–99% yield within 10–30 minutes and in most cases did not require purification by column chromatography. It is noted that in the absence of PS-Ph_3_P catalyst, the reaction gave complex mixture of products containing only 27–35% of the corresponding ester. Therefore, a significant increase in both the reaction rate and product yield was observed by using catalytic quantities of PS-Ph_3_P under ultrasonic irradiation conditions. The proposed mechanism of esterification involves formation of triazinephosphonium chloride, subsequent displacement with a carboxylate anion to give an acyloxytriazine, and an ensuing attack by methanol to afford the ester product with concomitant elimination of the hydroxyl derivative of TCT.

**Table 32:** Methyl esterification of carboxylic acids^a^

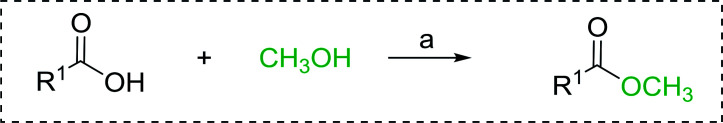					
Entry	R^1^CO_2_H	Yield (%)	Entry	R^1^CO_2_H	Yield (%)
1	PhCOOH	90	8	4-HOC_6_H_4_COOH	70
2	3,4-(MeO)_2_C_6_H_3_COOH	99	9	PhCH=CHCOOH	82
3	3-Me_2_NC_6_H_4_COOH	85	10	Ph(CH_2_)_4_COOH	97
4	4-ClC_6_H_4_COOH	97	11	4-HOPhCH_2_COOH	77
5	2-IC_6_H_4_COOH	92	12	Benzoylglycine	87
6	4-O_2_NC_6_H_4_COOH	96	13	Boc-Gly-OH	80
7	Nicotinic acid	80	14	Fmoc-Gly-OH	85

a Reagents and conditions: R^1^CO_2_H/TCT/Na_2_CO_3_/PS-Ph_3_P (1 : 1 : 2 : 0.1 molar ratio), MeOH, sonication, 50 °C, 10–30 min.

### 3.8. Reductive amination of aldehydes and ketones

Aldehydes and ketones have been shown to undergo indirect reductive amination using polymer-supported triphenylphosphine-palladium acetate complex PS-TPP–Pd(OAc)_2_ (10 mol% loading) as a heterogeneous and recyclable catalyst and sodium formate as a reducing agent (Table 33).^96^ The catalyst was easily prepared and isolated quantitatively as a yellowish solid by heating a mixture of PS-TPP and Pd(OAc)_2_ (P/Pd ratio of 4 : 1) in DMF to 45–50 °C for 4 h. The protocol involved two stages where the imine or iminium ion is first formed from the aldehyde or ketone, respectively, followed by PS-TPP–Pd(OAc)_2_-mediated catalytic reduction with sodium formate. Aromatic aldehydes and ketones gave higher yields than the corresponding aliphatic substrates and the catalyst was reusable over four consecutive cycles without any profound loss of catalytic activity.

**Table 33:** Reductive amination of aldehydes and ketones^a^

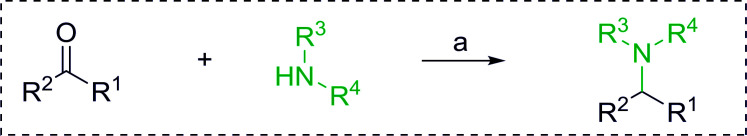				
Entry	Aldehyde/ketone	Amine	Product	Yield (%)
1	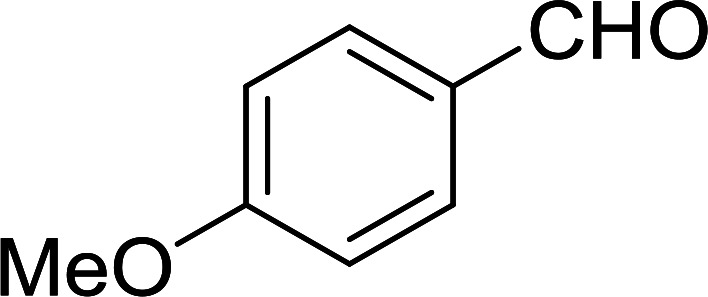	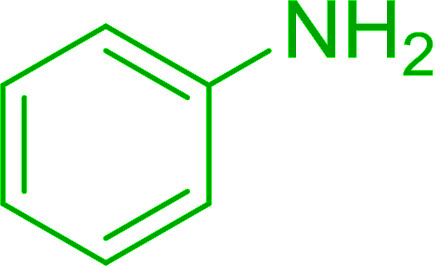	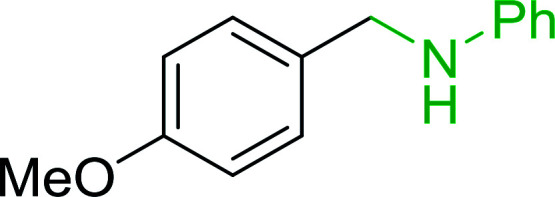	74
2	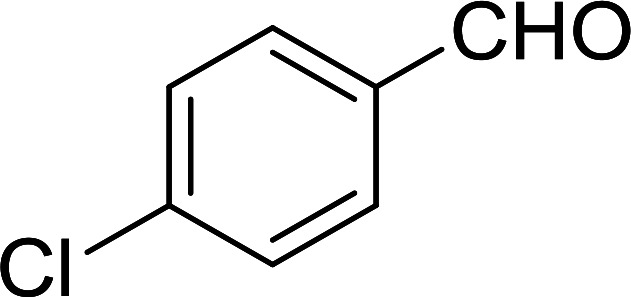	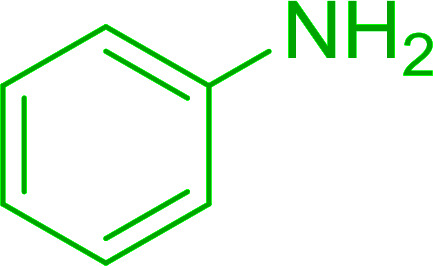	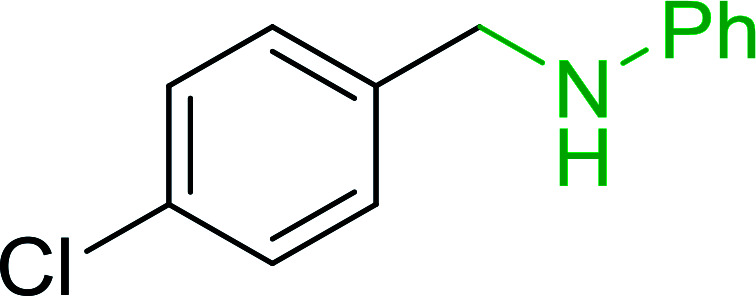	84
3	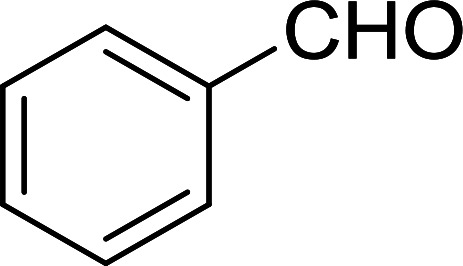	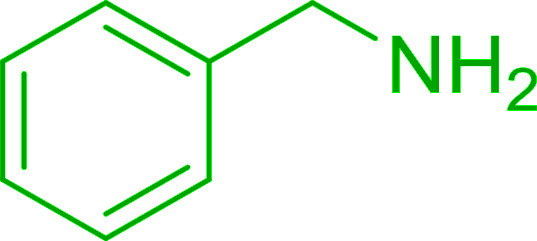	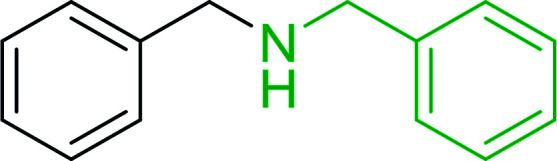	85
4	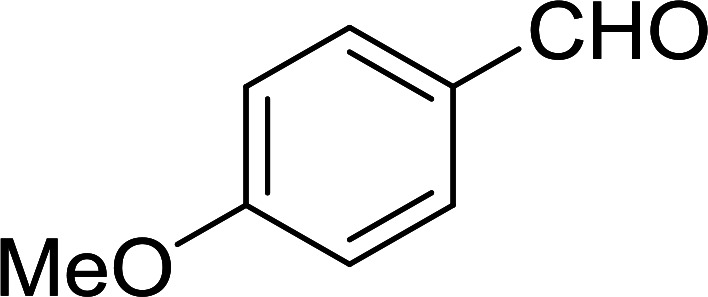	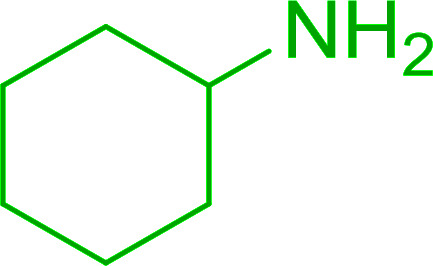	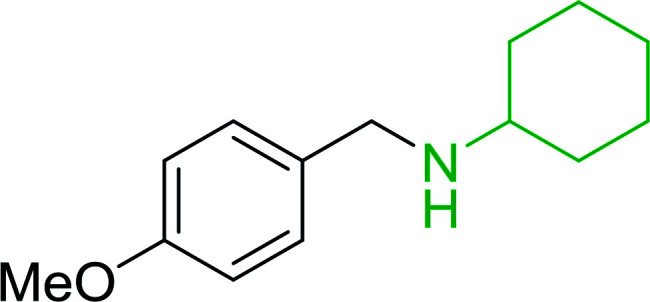	82
5	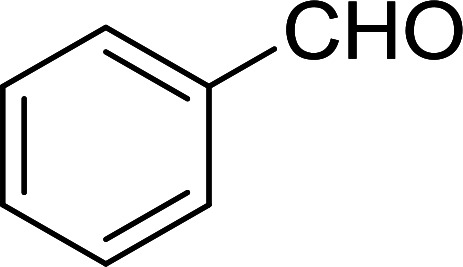	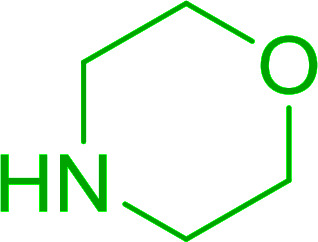	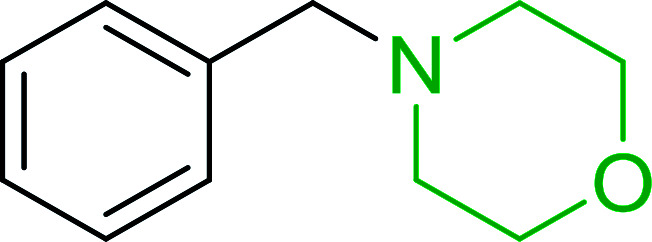	68
6	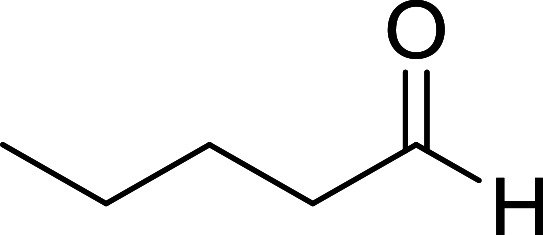	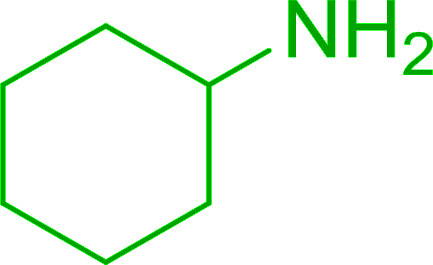	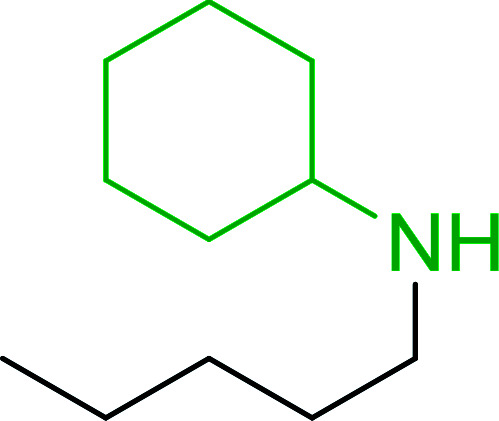	58
7	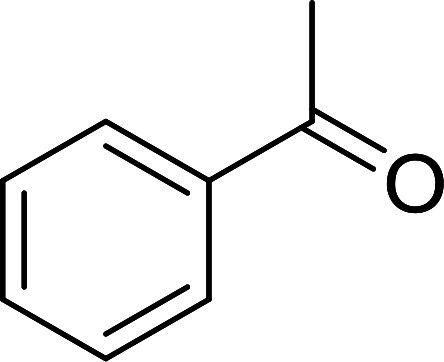	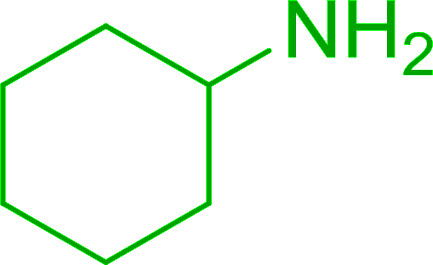	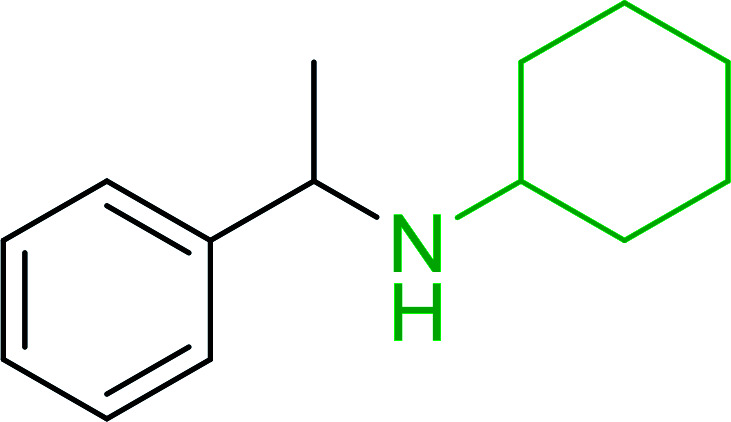	70
8	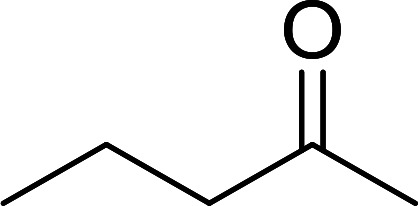	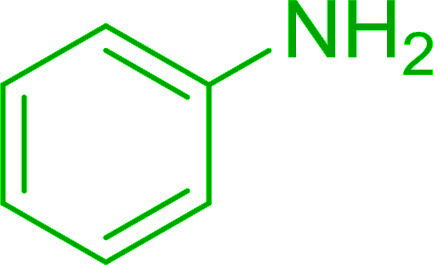	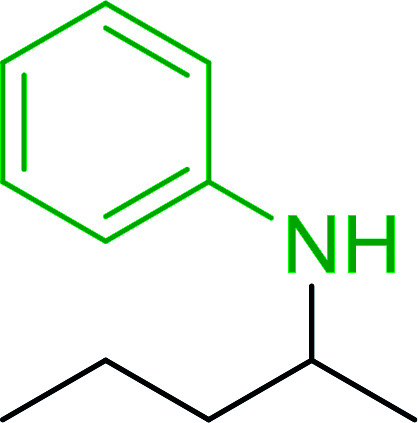	61

a Reagents and conditions: aldehyde or ketone (1 mmol), amine (1 mmol), molecular sieves 4Å (1 g), PS-TPP–Pd(OAc)_2_ (10 mol%), sodium formate (3 mmol), heating for 6 h at 85 °C, DMF (5 mL).

### 3.9. Synthesis of (*E*)-nitroalkenes by isomerisation of (*E*/*Z*)-nitro olefin mixtures

Treatment of (*E*/*Z*) mixture of nitroolefins with catalytic amounts of PS-TPP (0.1 equiv.) has been reported to produce pure (*E*)-nitroalkenes in a number of cases (Table 34).^97^ The mechanism of isomerisation involves addition of nucleophilic PS-TPP to the activated double bond, interconversion to the appropriate intermediate, and subsequent elimination reaction to afford the nitroalkene. The stereoselectivity of the *E*/*Z*-isomerisation was retained in all cases (entries 1–4) except when a phenyl group was introduced in α-position (entry 5) or a phenylthio group in the β-position (entry 6). In these examples, an *E*/*Z*-ratio of 90 : 10 and 65 : 35 was observed, respectively.

**Table 34:** Isomerization of (*E*/*Z*)-nitroalkenes into pure (*E*)-nitroalkenes with PS-TPP^a^

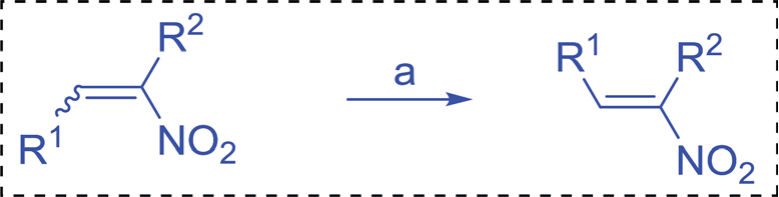					
Entry	R^1^	R^2^	(*E*/*Z*)-Nitroolefin mixture	Time (h)	Product (*E*/*Z*)
1	Me	Pr	55 : 45	20	100 : 0
2	Me	(CH_2_)_2_COOEt	90 : 10	20	100 : 0
3	Et	Pr	60 : 40	40	100 : 0
4	Me	Ph	80 : 20	20	100 : 0
5	Ph	Pr	80 : 20	20	90 : 10
6	Me	SPh	0 : 100	48	65 : 35

a Reagents and conditions: 1 : 0.1 molar ratio of nitroalkene : PS-TPP, RT, CH_2_Cl_2_, 100% yield.

### 3.10. Debromination of α-bromo ketones

An effective method for the debromination of various α-bromo ketones using PS-TPP was described by Salunkhe *et al.*^98^ The debromination reaction proceeded in high yields (85–97%) in anhydrous benzene using equimolar amounts of the resin-bound polymer. Pure ketones were isolated (Table 35) following filtration of the polymeric phosphine oxide by-product and solvent removal *in vacuo*.

**Table 35:** Debromination of various α-bromo ketones a

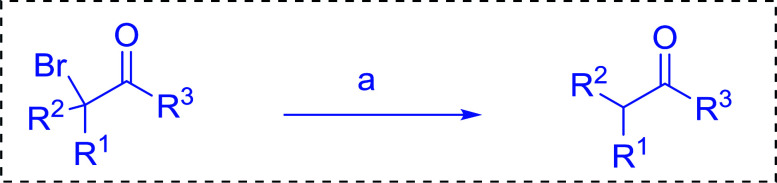			
Entry	α-Bromo ketone	Ketone product	Yield (%)
1	3-Bromocamphor	Camphor	97
2	2-Bromocyclohexanone	Cyclohexanone	89
3	*p*-Nitrophenacyl bromide	*p*-Nitroacetophenone	85
4	Phenacyl bromide	Acetophenone	96

a Reagents and conditions: α-bromo ketone, PS-TPP, benzene, RT, 30–50 min.

### 3.11. Henry reaction of aldehydes with nitroalkanes

Polymer-supported triphenylphosphine was found to react with ethyl acrylate in a stoichiometric ratio to generate an ethyl acrylate conjugated polystyryldiphenylphosphine (PDPP–EA) complex *in situ*.^68^ The complex was used to catalyze the synthesis of 2-nitroalcohols from the reaction of various nitroalkanes and aldehydes (Henry reaction) under solvent-free conditions (Table 36).^99^ The catalyst was easily prepared in under 10 min by stirring an equimolar mixture of PS-TPP and ethyl acrylate. Although the ensuing Henry reaction proceeded well in a number of solvents, the highest yields were obtained without solvent and optimum efficiency of the method required 10 mol% of the catalyst. Resin-bound triphenylphosphine could be recovered from the reaction and reused up to five times without loss of activity. The reaction protocol was applied for the reaction of primary and secondary niroalkanes with aliphatic aldehydes and aromatic aldehydes bearing various electron-donating and electron-withdrawing substituents (Table 36). Good yields were observed with all types of substrates, including those with some degree of steric hindrance (entry 5).

**Table 36:** Henry reaction of various aldehydes with nitroalkanes using 1 and ethyl acrylate^a^

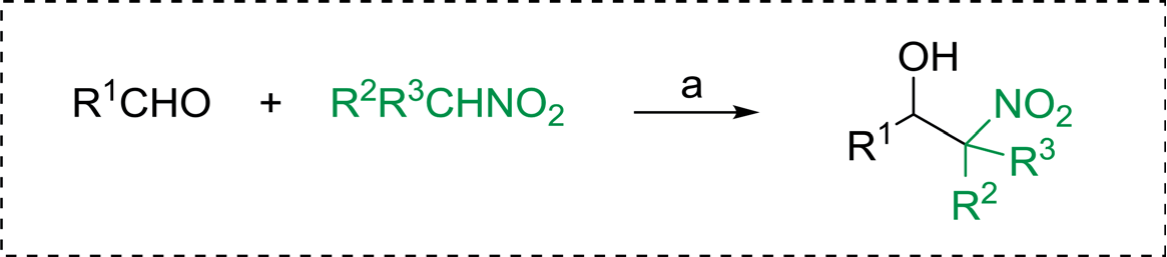				
Entry	Aldehyde	Nitroalkane	Product	Yield (%)
1	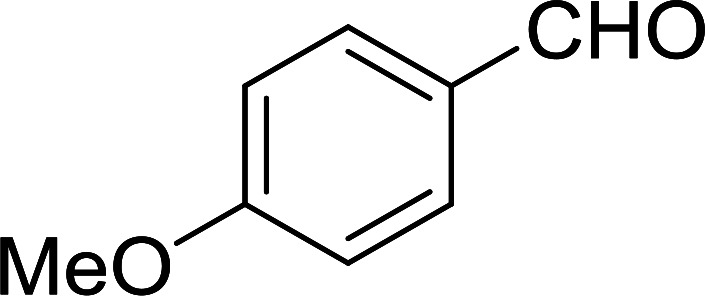	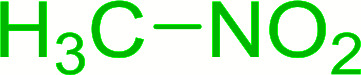	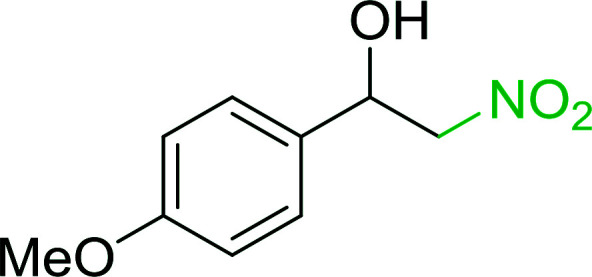	88
2	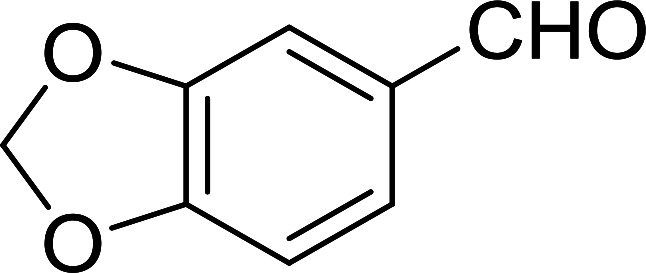	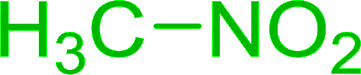	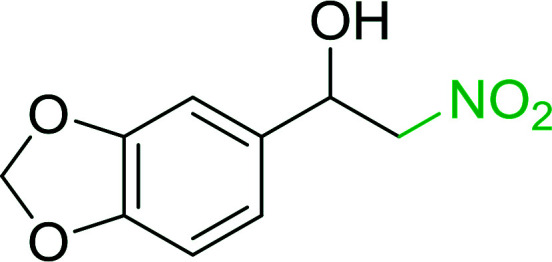	88
3	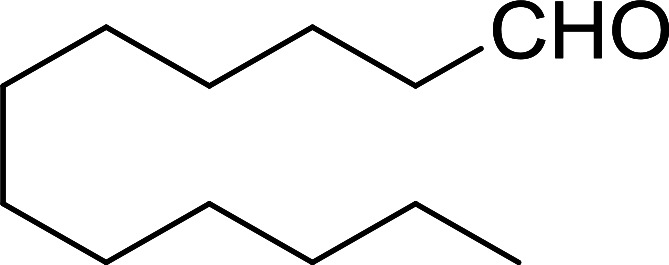	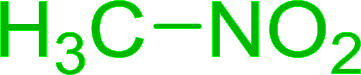	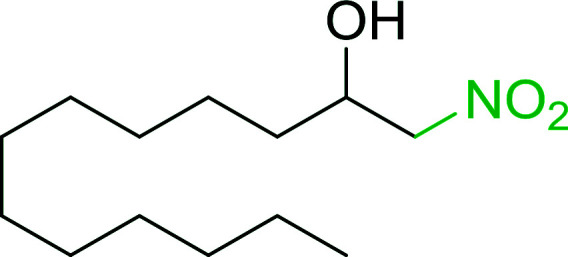	86
4	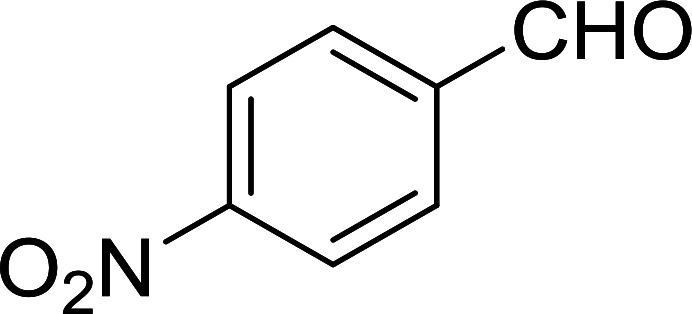	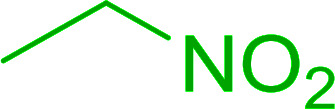	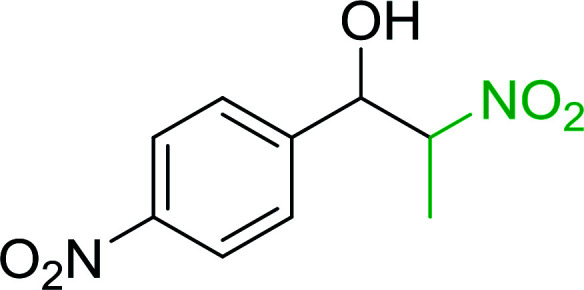	90
5	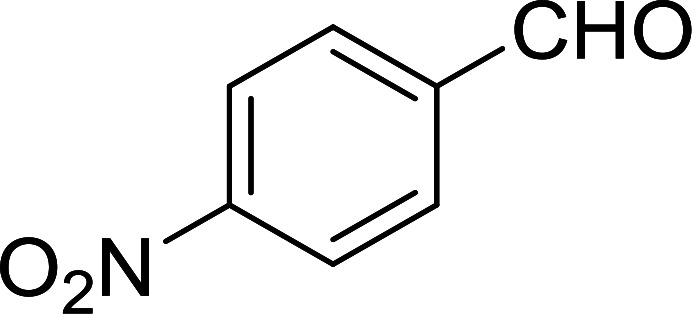	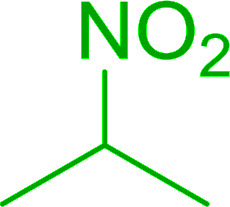	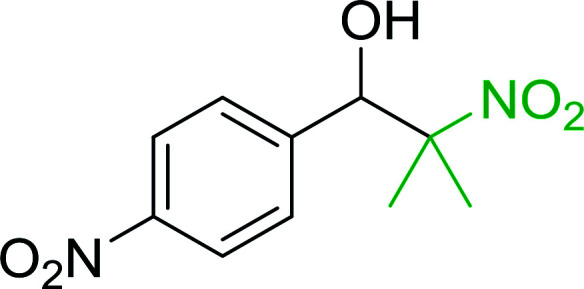	91
6	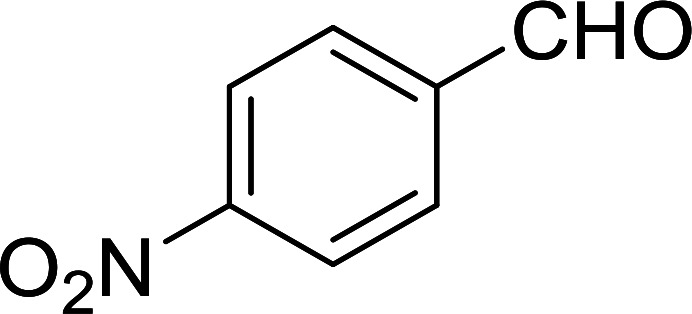	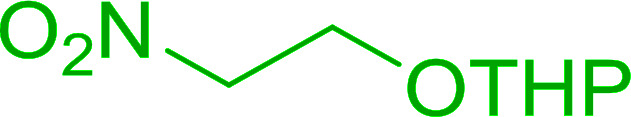	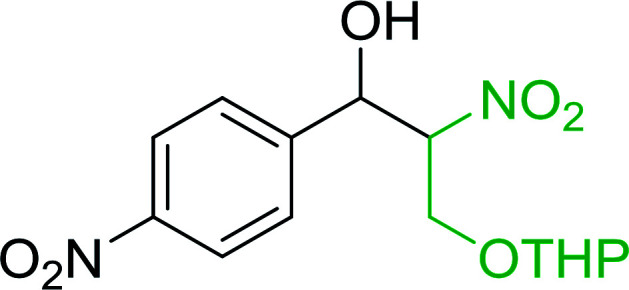	84
7	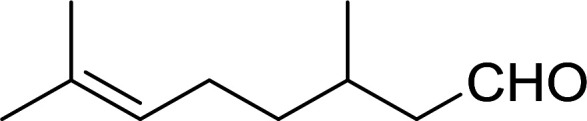	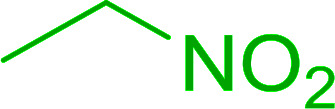	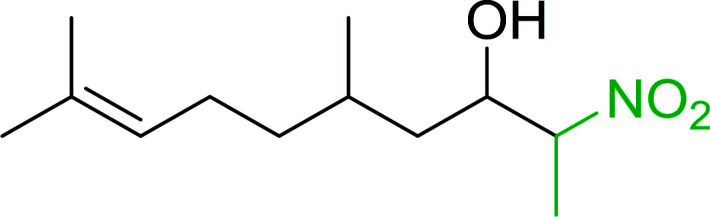	88

a Reagents and conditions: 1 : 2 : 0.1 : 0.1 molar ratios of aldehyde : nitroalkane : PS-TPP : ethyl acrylate, solvent-free conditions, RT, 2–4 h.

### 3.12. Hydroperoxide reduction

**Scheme 17:**
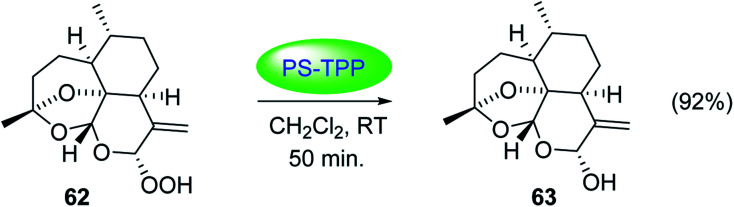
Hydroperoxide reduction to alcohol with PS-TPP.

Reduction of hydroperoxides into alcohols with PS-TPP represents a very mild approach to such a transformation especially in complex synthetic settings. Galal *et al.* described the reduction of hydroperoxide 62 using PS-TPP (Scheme 17).^100^ The reaction was carried out rapidly (50 min) at room temperature and afforded the alcohol product 63 in 92% yield.

### 3.13. Ozonide reduction

One of the most popular approaches for the preparation of carbonyl compounds from alkenes involves ozonolysis of a double bond, followed by reductive cleavage of the resulting ozonide with a reducing agent.^101^ Santaniello *et al.* used PS-TPP to reduce ozonides to the corresponding carbonyl products (Table 37).^102^ Various aldehydes and ketones were obtained in high yields (80–92%) and were virtually pure.

**Table 37:** Synthesis of carbonyl compounds from alkenes^a^

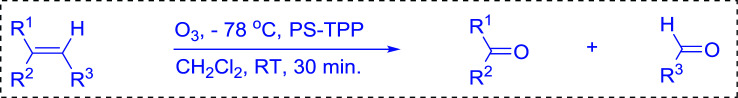				
Entry	R^1^	R^2^	R^3^	Yield (%)
1	Ph	H	H	80
2	Ph	H	H	86
3	C_9_H_19_	CH_3_	H	92
4	C_5_H_11_	CH_3_	H	88
5	C_7_H_15_	H	C_7_H_15_	91
6	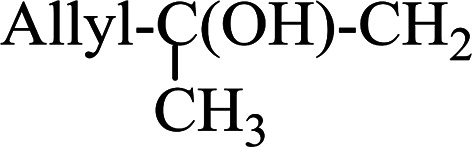	H	H	90

a Reagents and conditions: 1 : 1 molar ratio of PS-TPP : alkene.

### 3.14. Stereoselective isomerization of α,β-ynones to (*E*,*E*)-α,β-γ,δ-dienones using PS-TPP

PS-TPP has been successfully used as an organocatalyst for the stereoselective isomerization of α,β-ynones to (*E*,*E*)-α,β-γ,δ-dienones.^103^ The amount of PS-TPP (20 mol%) and the reaction temperature (80 °C) were critical to the isomerization process. Thus, under these optimized conditions, the conversion of several ynones to the corresponding dienones was accomplished in moderate to good yields (Table 38). Ynones with aromatic and heteroaromatic substituents could be isomerized in good yields (entries 1–3) whilst aliphatic ynones showed lower yields (entries 4–6). The recovered PS-TPP was reused several times as it retained its catalytic activity, although the catalytic capacity was reduced significantly after repeated use as reflected by a steady decrease in product yield.

**Table 38:** Stereoselective conversion of α,β-ynones to (*E*,*E*)-α,β-γ,δ-dienones using PS-TPP^a^

Entry	Ynones	Dienones	Yield (%)
1	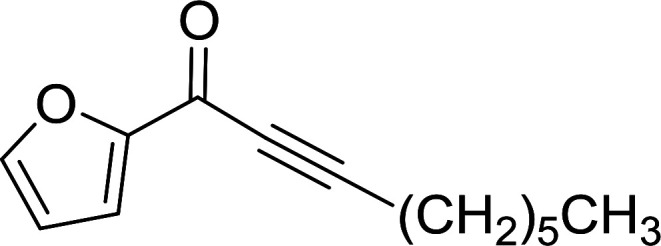	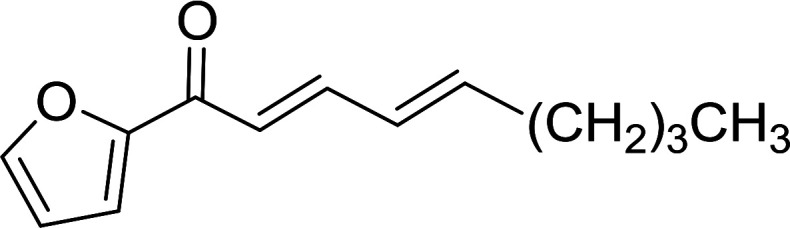	79
2	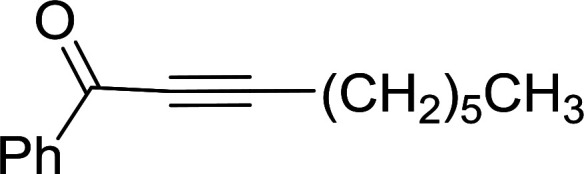	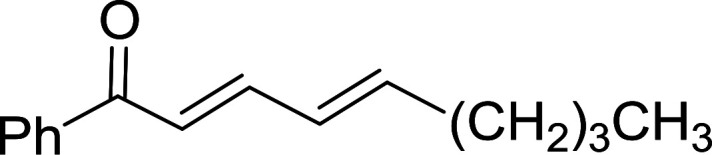	81
3	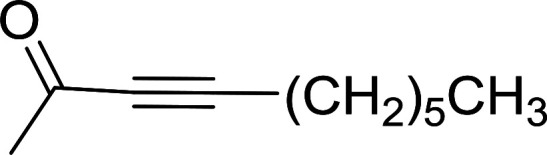	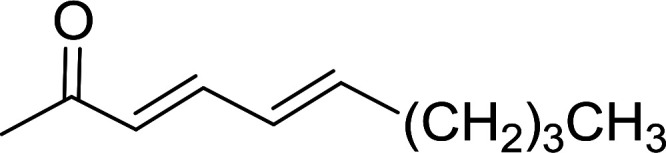	80
4	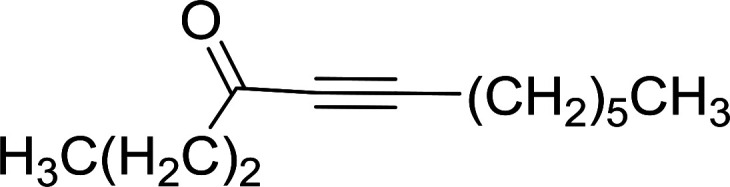	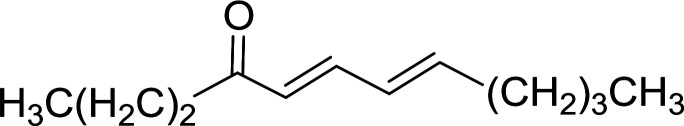	64
5	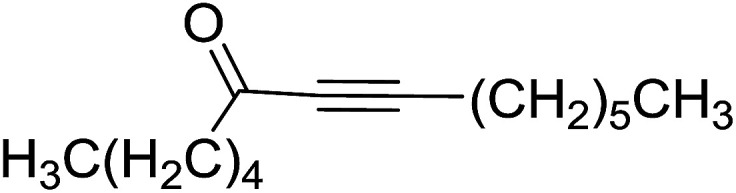	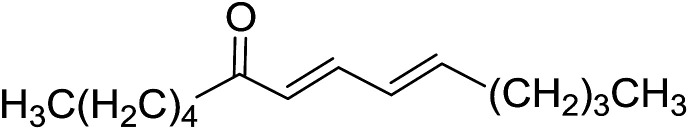	51
6	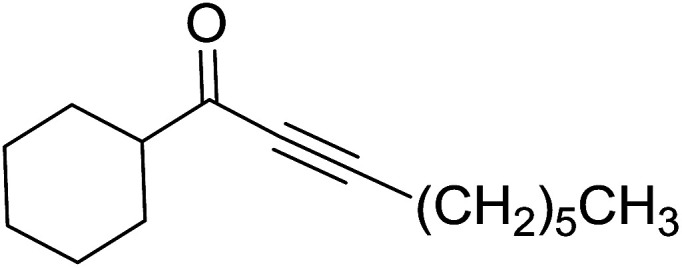	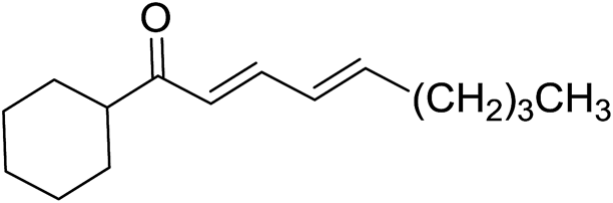	47

a Reagents and conditions: PS-TPP (20 mol%), ynones (1 mmol), toluene, 80 °C, 18 h.

### 3.15. γ-Addition of pronucleophiles to alkynoate

Some years ago, Trost *et al.* discovered the ability of triphenylphosphine to redirect the conjugate addition of various carbon nucleophiles from the normal β-position to the γ-position of 2-alkynoates^104^ and applied the new method to the construction of tetrahydrofuran and tetrahydropyran rings^105^ which show widespread occurrence in many classes of natural products. Nitrogen pronucleophiles also underwent successful γ-addition reactions in the same manner to afford nitrogen-containing products.^106^ In these transformations, the nucleophilic triphenylphosphine, which was used in catalytic amounts, first added to the triple bond of an α,β-unsaturated system and finally was eliminated from the reaction product after a series of transformations. As an improvement to the γ-addition reactions which require tertiary phosphine homogeneous catalysts, regarded as the major limitation of the methodology, Li *et al.* efficiently carried out Trost's γ-addition of various carbon pronucleophiles to methyl 2-butynoate catalyzed by PS-TPP.^107^ The reactions were run in aqueous media using water : toluene (5 : 1) under microwave irradiation conditions (Table 39) and conventional heating as well. The polymer catalyst was recovered and reused in subsequent reactions without loss of activity. In general, high yields were obtained with all pronucleophiles for which p*K*_a_ < 16, although increased steric hindrance or lowered kinetic acidity of the pronucleophile compromised the yield (entries 5 and 6).

**Table 39:** PS-TPP-catalyzed γ-addition of pronucleophiles to methyl 2-butynoate^a^

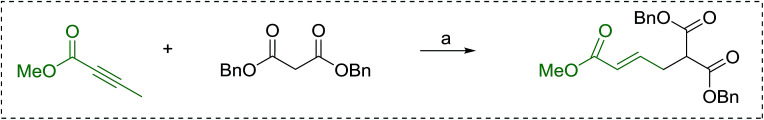			
Entry	Pronucleophile	Product	Yield (%)
1	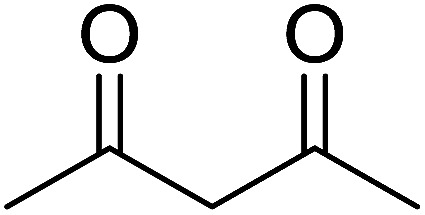	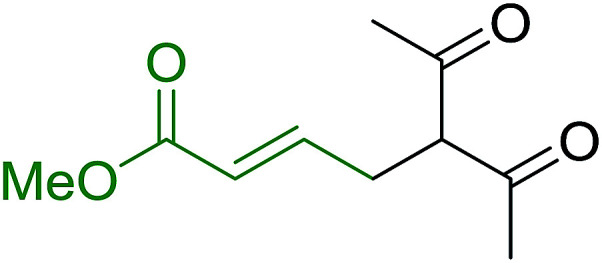	97
2	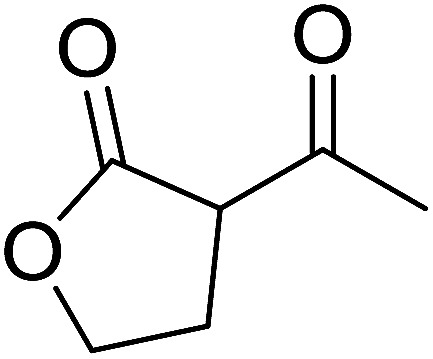	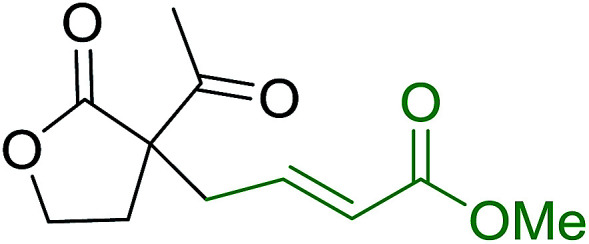	96
3	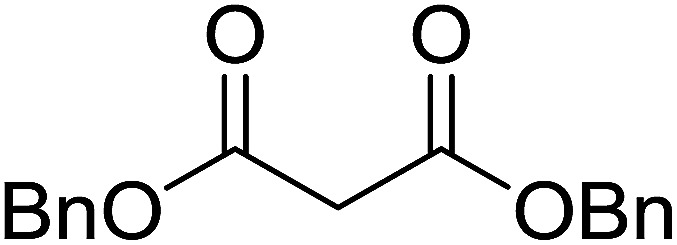	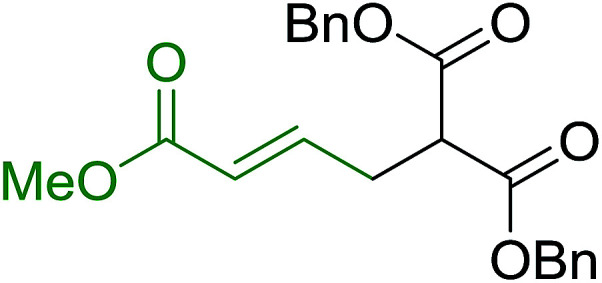	82
4	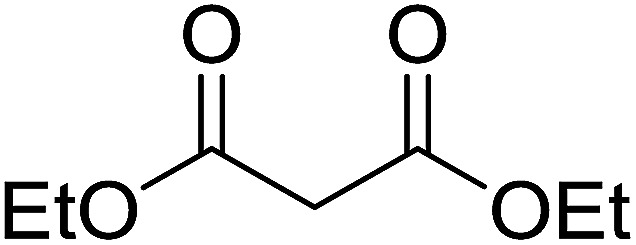	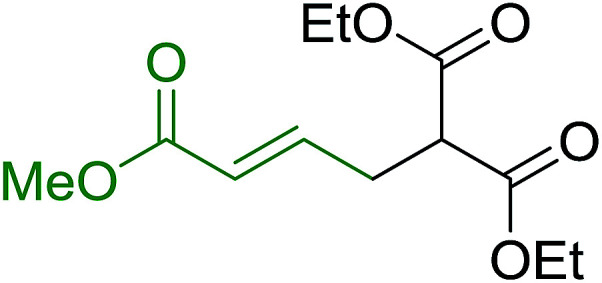	83
5	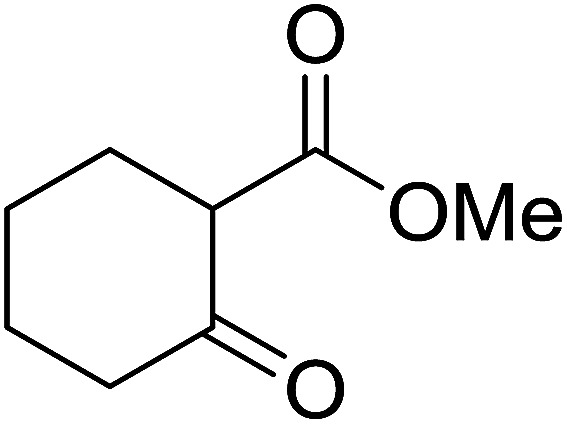	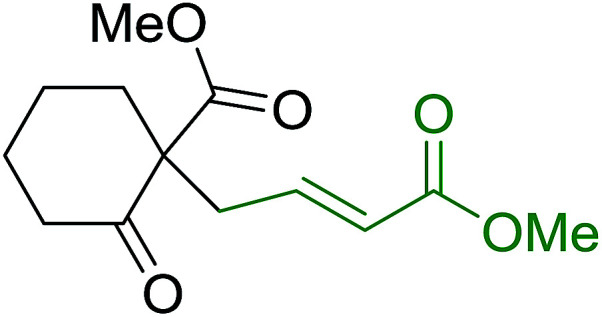	61
6	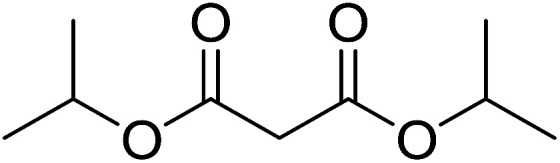	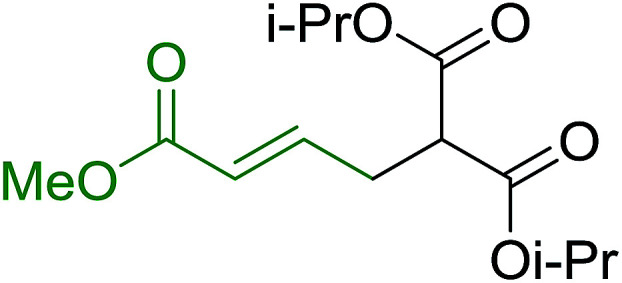	67

a Reagents and conditions: 1 : 1 : 0.35 molar ratios of methyl 2-butynoate : pronucleophile : PS-TPP, H_2_O/toluene (5 : 1), MW, 150 °C, 45–90 min.

## 4. Application of PS-TPP in heterocycle synthesis

### 4.1. Synthesis of 1,3,4-thiadiazole-2,5-dicarbonitrile and thiazole-2,4,5-tricarbonitrile

**Scheme 18:**
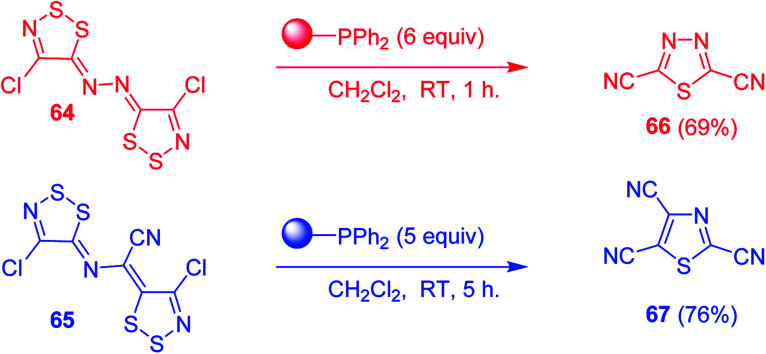
Preparation of 1,3,4-thiadiazole-2,5-dicarbonitrile and thiazole-2,4,5-tricarbonitrile.

1,3,4-Thiadiazole-2,5-dicarbonitrile (66) shows interesting biological activity and is an excellent fungicide for *Aspergillus* (Scheme 18).^108^ While 66 may be prepared in few steps from commercially available starting material, reactivity on silica gel and hydration of one of the nitrile groups (converting to the amide) during chromatography presents purification and isolation challenges.^109^ Koutentis *et al.* showcased the utility of PS-TPP in the preparation of 66 and the novel analog, thiazole-2,4,5-tricarbonitrile (67), from the corresponding precursors 1,2-bis(4-chloro-5*H*-1,2,3-dithiazol-5-ylidene)hydrazine (64) and 2-(4-chloro-5*H*-1,2,3-dithiazol-5-ylidene)-2-(4-chloro-5*H*-1,2,3-dithiazol-5-ylideneamino)acetonitrile (65), respectively. Thus, treatment of dithiazole 64 with 6 molar equivalents of PS-TPP in dry CH_2_Cl_2_ and stirring for 1 h afforded 66 in 69% yield. Similarly, 67 was prepared in 76% yield under the same conditions, albeit lower amount of PS-TPP (5 equiv.) was used.

### 4.2. Synthesis of 3-aminoindole-2-carbonitriles

As a part of ongoing research into expanding the use of 1,2,3-dithiazoles in heterocyclic ring transformations, Koutentis and Michaelidou employed PS-TPP in the conversion of 2-(4-chloro-5*H*-1,2,3-dithiazolylideneamino)benzonitriles into 3-aminoindole-2-carbonitriles (Table 40).^110^ Indoles exhibit a wide spectrum of biological activities and are important heteroarenes.^111^ Moreover, substituted 3-aminoindole-2-carbonitriles are scant and strategies for their synthesis is necessary. In light of this, Koutentis undertook a detailed study where various substituted 2-(4-chloro-5*H*-1,2,3-dithiazolylideneamino)benzonitriles (68) reacted with PS-TPP (5 equiv.) in the presence of water (2 equiv.) to afford the desired 3-aminoindole-2-carbonitriles (69) together with anthranilonitriles as a side product.

**Table 40:** Preparation of 3-aminoindole-2-carbonitriles from 1,2,3-dithiazoles

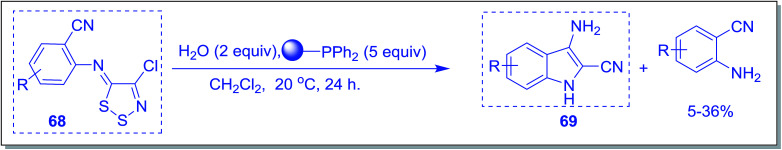		
Entry	R	Yield (%) of 69
1	H	26
2	5-NO_2_	55
3	3-Br-5-NO_2_	27
4	4-Cl	39
5	5-Cl	13
6	6-Me	17
7	4-MeO	29

### 4.3. Synthesis of substituted 2-phenylbenzothiazoles

Substituted 2-phenylbenzothiazoles are of tremendous importance to medicinal chemistry and are considered as privileged pharamacophore.^112^ Several 2-phenylbenzothiazole derivatives have been shown to exhibit selective and potent biological activities, culminating in their clinical evaluation against certain types of tumours. For instance, 2-(4-amino-3-methylphenyl)-5-fluorobenzothiazole emerged as a lead compound against breast and ovarian cancer and had undergone phase 1 clinical trial in the U.K. in the form of its water soluble l-lysyl amide prodrug (Phortress).^113,114^ 2-(3,4-Dimethoxyphenyl)-5-fluorobenzothiazole (PMX 610) is another clinical candidate with selective cytotoxicity profile reminiscent of the related 2-(4-aminophenyl)benzothiazole series.^115^ In addition, the radioactive ^11^C-labelled 6-hydroxy-4-(methylaminophenyl)benzothiazole known as Pittsburgh compound B has been developed for use in positron emission tomography (PET) scans to image beta-amyloid plaques in neuronal tissue as a non-invasive method of investigating Alzheimer's disease and other neurodegenerative conditions. 2-Phenylbenzothiazoles can be synthetically accessed *via* condensation of 2-aminophenol with benzaldehydes or benzoic acid derivatives, followed by oxidation of the resulting dihydrobenzothiazole. However, the synthesis of substituted 2-phenylbenzothiazoles is challenging because the requisite substituted 2-aminothiophenols are prone to oxidation. Westwell *et al.* developed an approach to substituted 2-phenylbenzothiazoles where PS-TPP and *p*-TSOH were used to promote a reaction between stable 2-aminothiophenol disulfides and benzaldehydes (Table 41).^116^ The procedure features a variety of substituents on both the benzothiazole and phenyl rings with yields ranging between 80–97%.

**Table 41:** Synthesis of substituted 2-phenylbenzothiazoles^a^

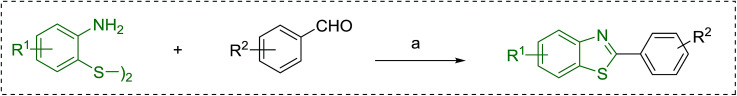			
Entry	R^1^	R^2^	Yield (%)
1	H	4-OMe	97
2	H	4-OH	97
3	H	3-NO_2_	94
4	H	4-Br	86
5	6-F	4-OMe	88
6	6-F	4-OH	87
7	6-F	4-NO_2_	93
8	6-F	4-Br	92
9	6-F	4-CN	89
10	6-OEt	4-OMe	87
11	6-OEt	4-OH	94
12	6-OEt	4-NO_2_	87
13	6-OEt	4-Br	80

a Reagents and conditions: 1 : 1 : 1 : 0.2 molar ratios of aldehyde : PS-TPP : substituted bis(2-aminophenyl)disulfide : *p*-TSOH, toluene/DMF (1 : 1), reflux, 2 h.

### 4.4. Synthesis of 1,2,4-oxadiazoles

1,2,4-Oxadiazole are biologically interesting heterocycles that act as bioisosteres for amides or esters in medicinal chemistry.^117^ These compounds comprise an important structural motif and have been incorporated into muscarinic^118^ and benzodiazepine^119^ receptor agonists, serotoninergic (5-HT3) antagonists,^120^ as well as antirhinovirals.^117^ They have also found applications as peptide mimetics.^121^ Because of their biological properties, Wang *et al.* developed a polymer-assisted solution phase reaction protocol for the synthesis of 1,2,4-oxadiazole in an expeditious manner.^122^ They found that under microwave heating conditions at 100 °C for 5 min, various carboxylic acids are converted into the corresponding acid chlorides in nearly quantitative yields by treatment with trichloroacetonitrile and PS-PPh_3_. Thus, in the first step, the preparation of 1,2,4-oxadiazoles involved generating the acid chlorides *in situ* from the corresponding carboxylic acids using PS-PPh_3_/CCl_3_CN. Subsequent addition of the amidoxime and DIEA in THF and heating in the microwave at 150 °C for 15 min afforded the desired 1,2,4-oxadiazole in excellent yield (Table 42). It is noteworthy that the PS-PPh_3_ resin from the first step did not interfere with the reaction in the second step, thereby allowing the transformation to be performed in on pot.

**Table 42:** Synthesis of 1,2,4-oxadiazoles from carboxylic acids and amidoximes^a^

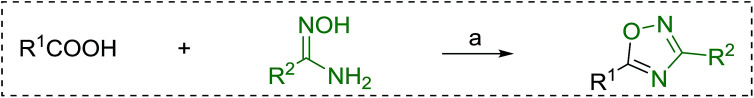				
Entry	Carboxylic acid	Amidoxime	Product	Yield (%)
1	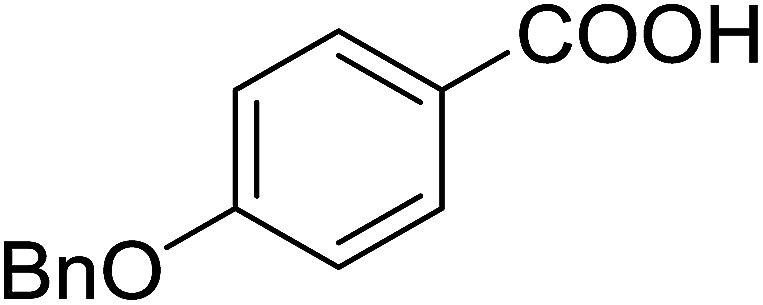	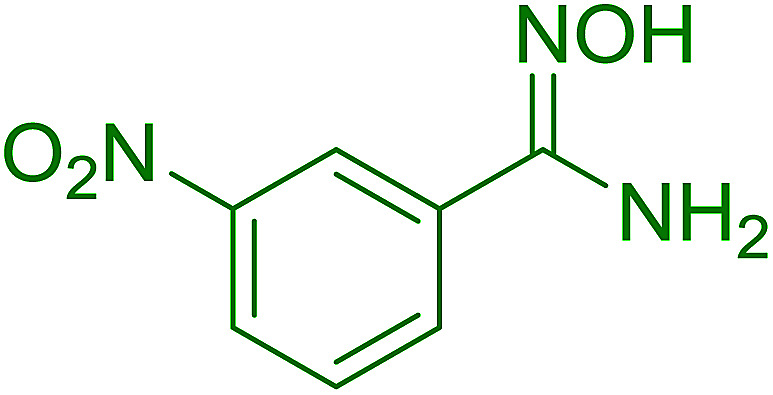	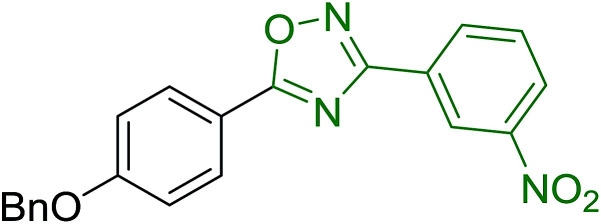	90
2	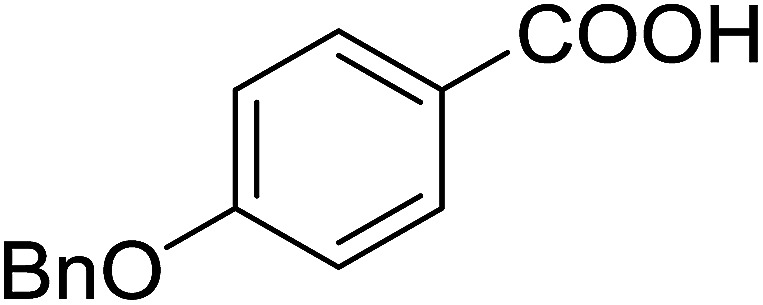	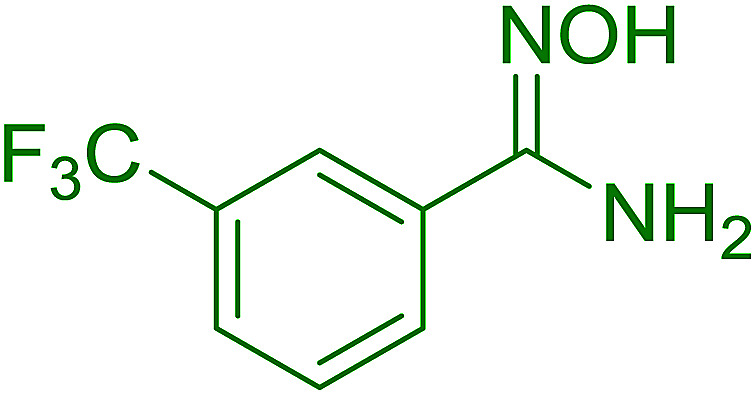	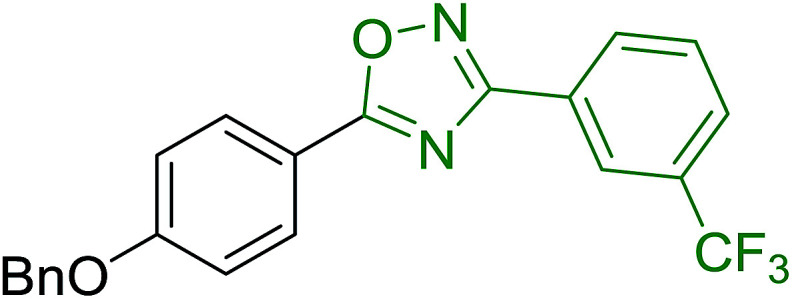	80
3	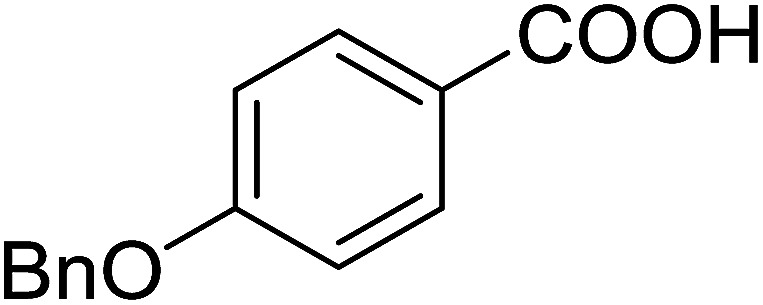	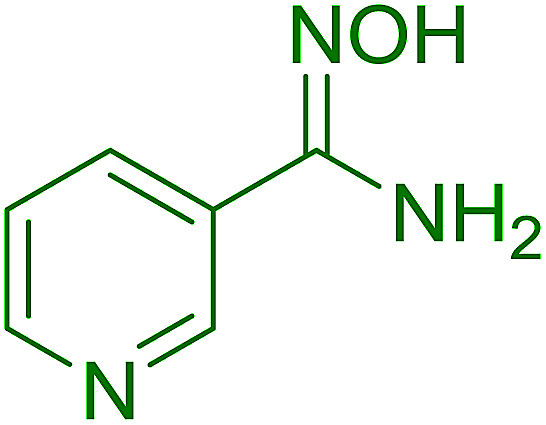	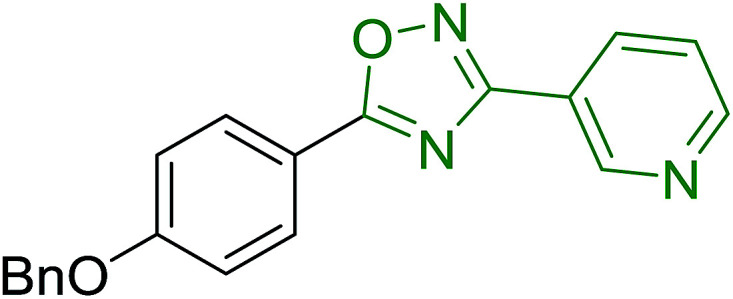	94
4	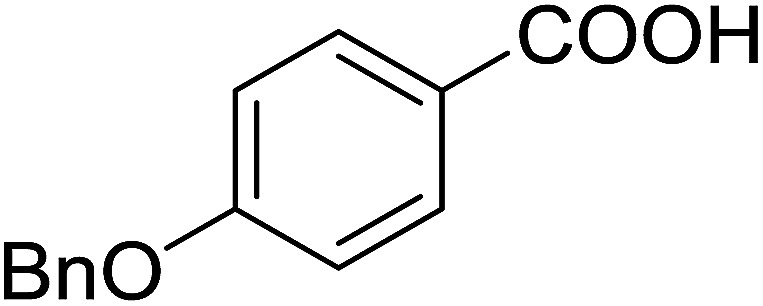	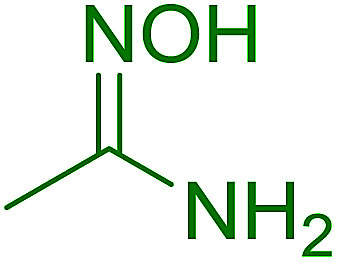	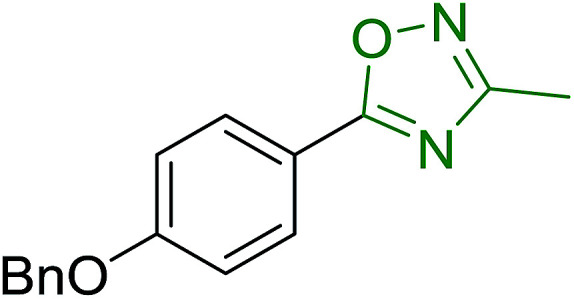	80
5	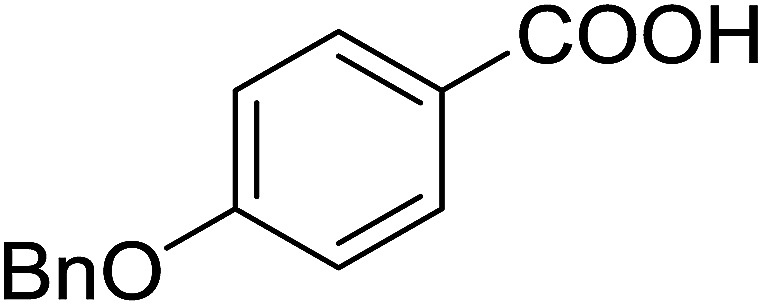	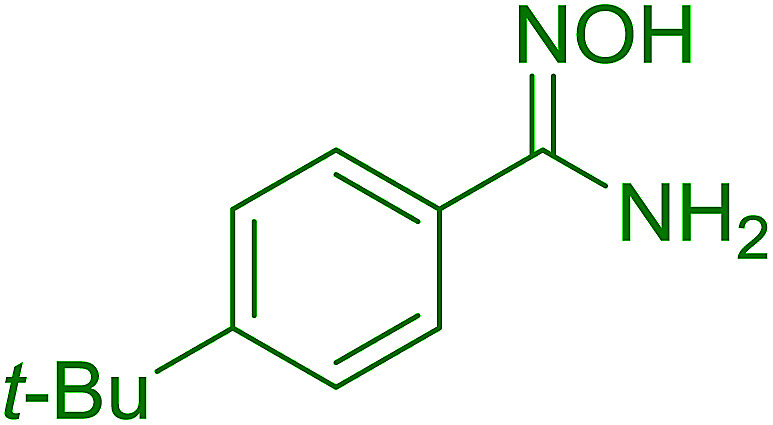	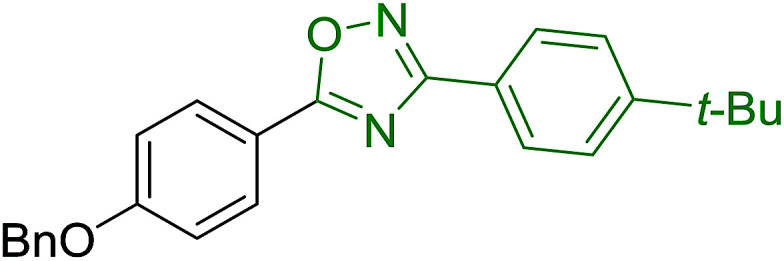	93
6	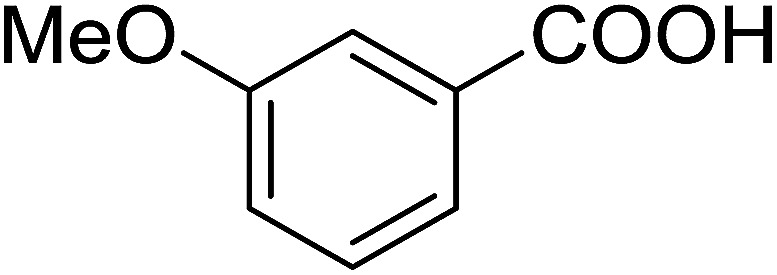	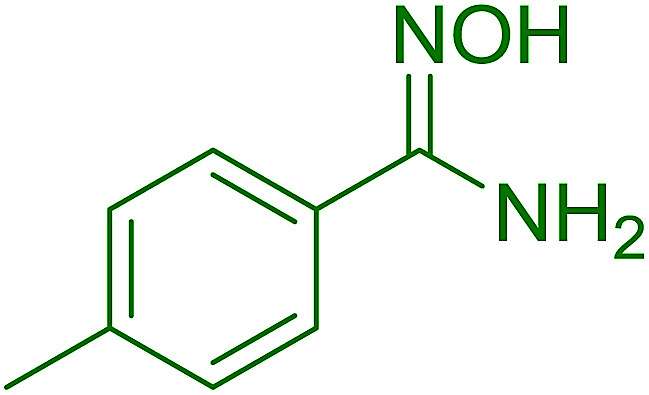	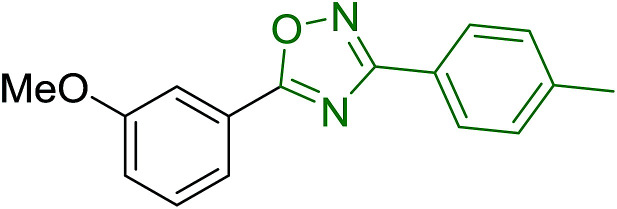	98
7	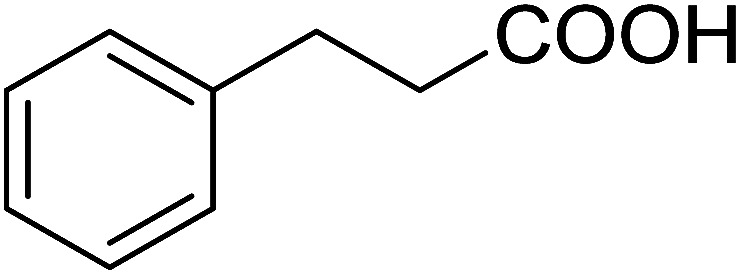	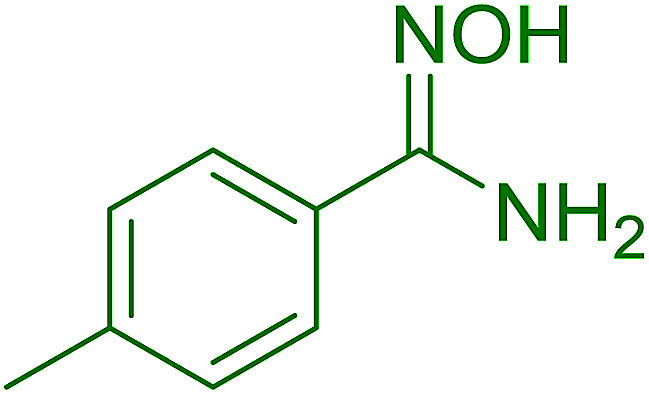	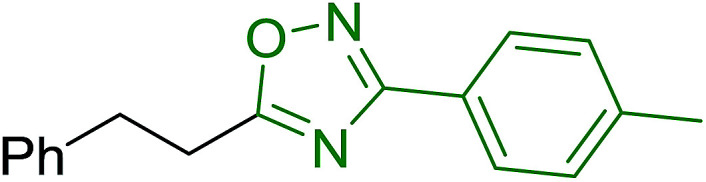	93
8	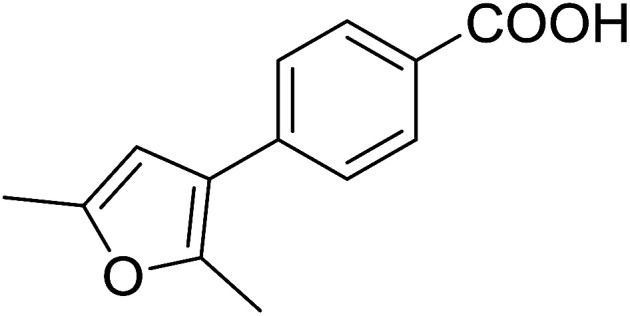	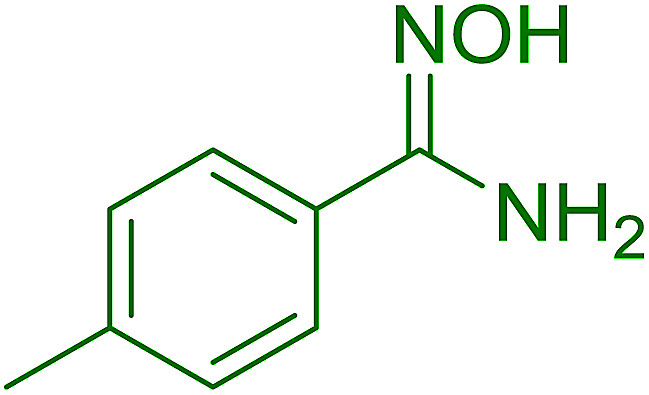	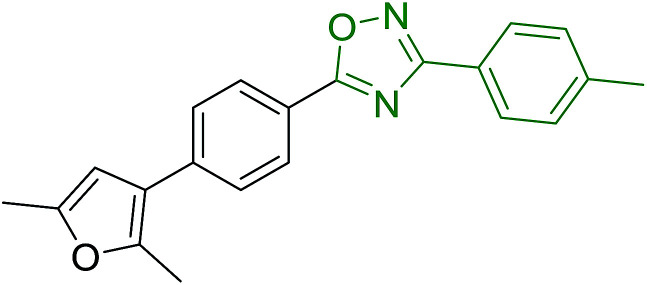	90
9	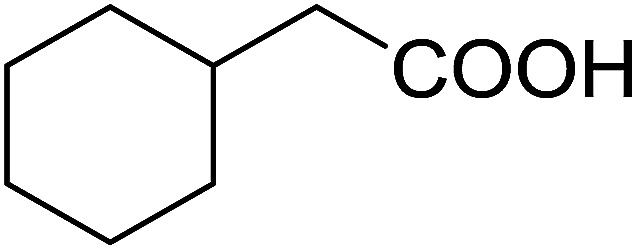	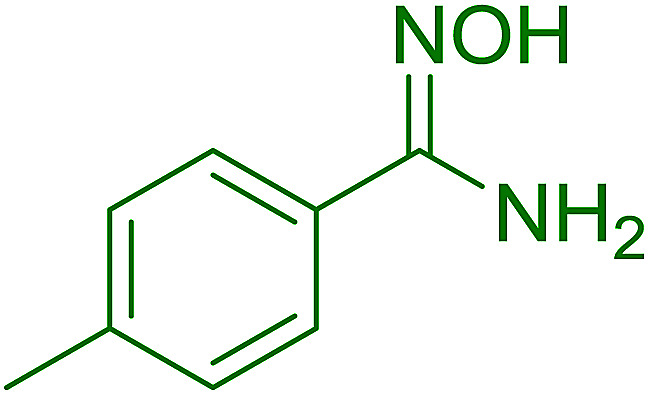	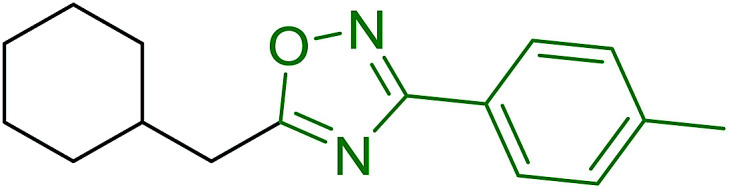	97
10	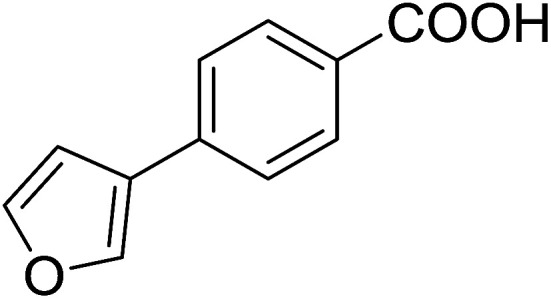	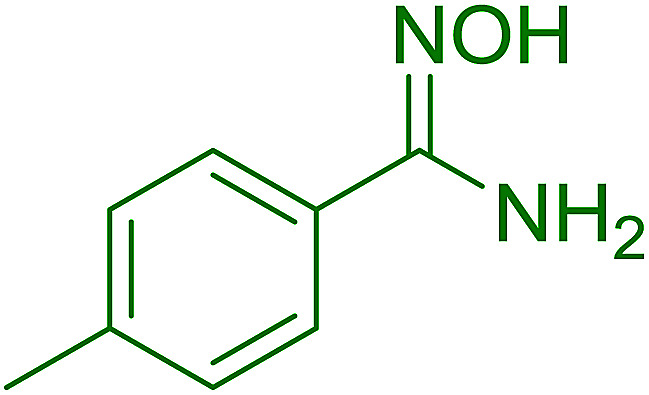	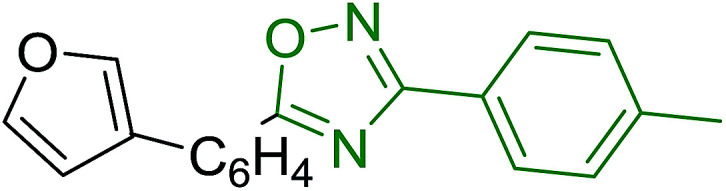	96
11	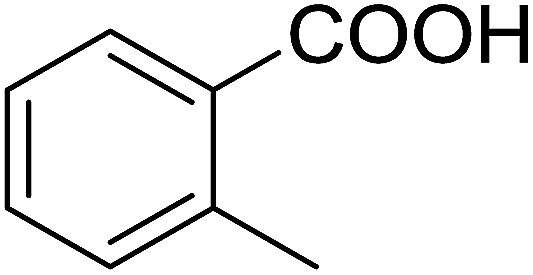	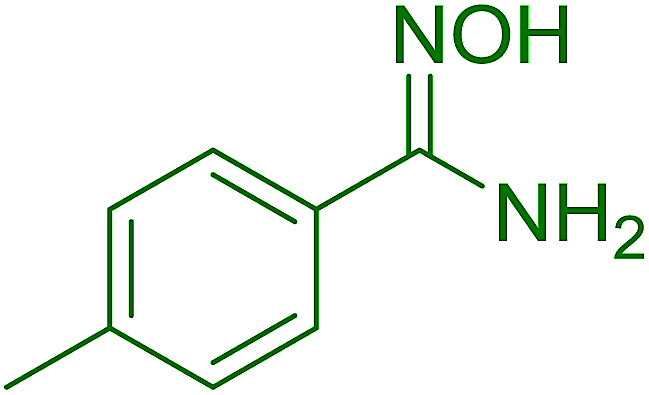	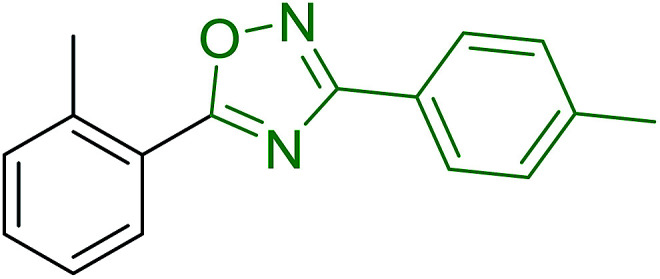	98
12	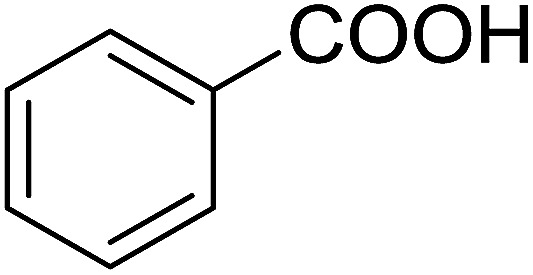	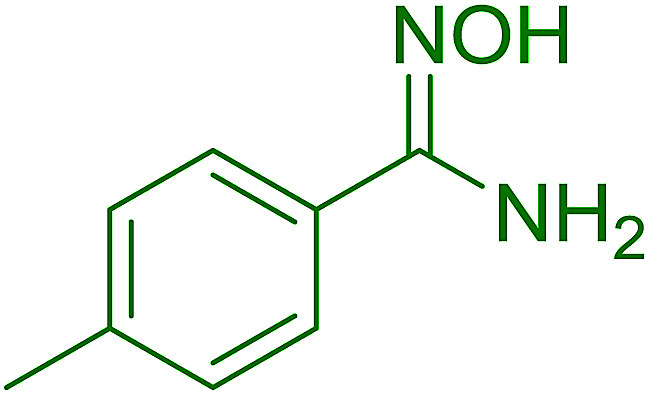	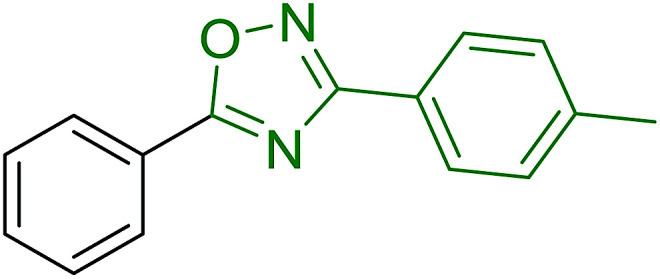	95
13	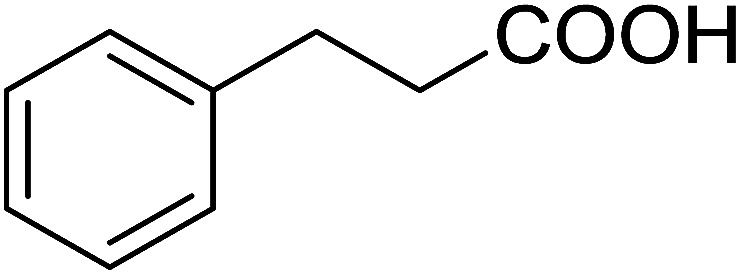	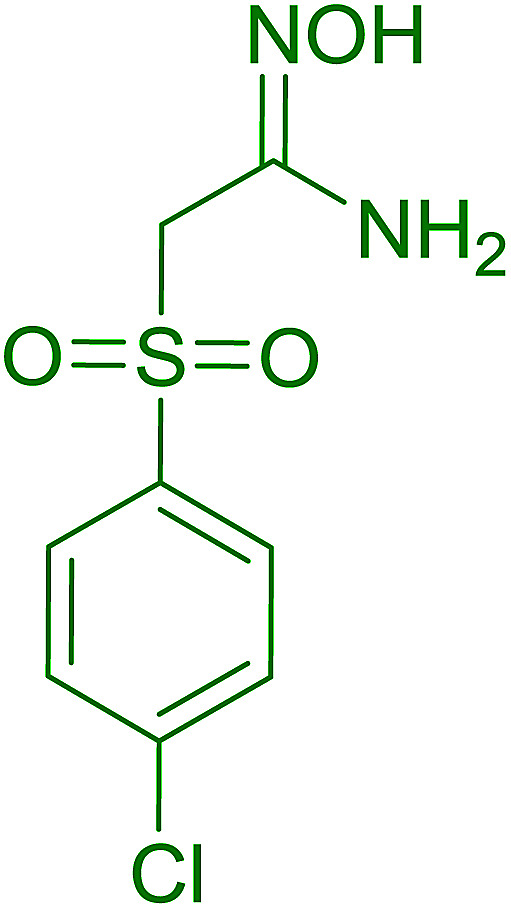	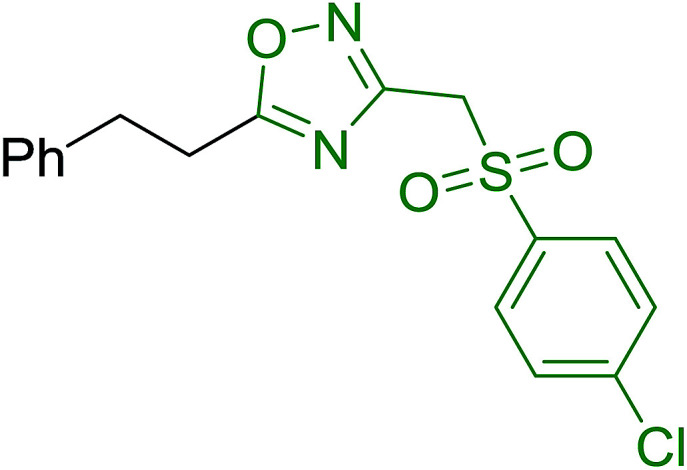	83
14	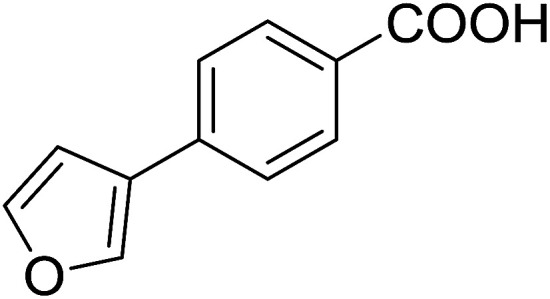	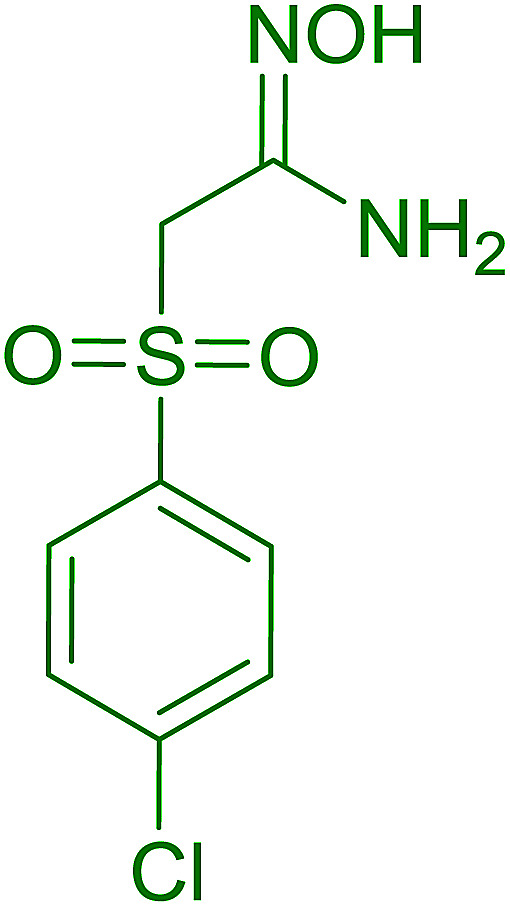	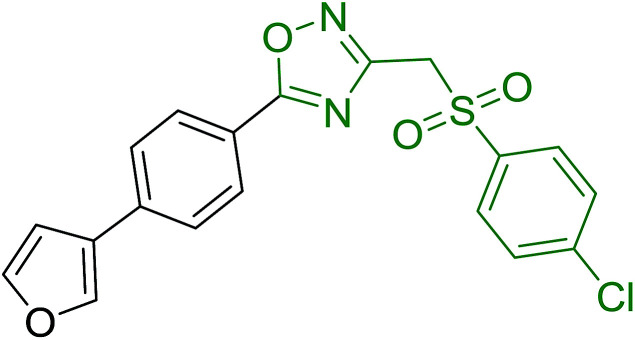	77
15	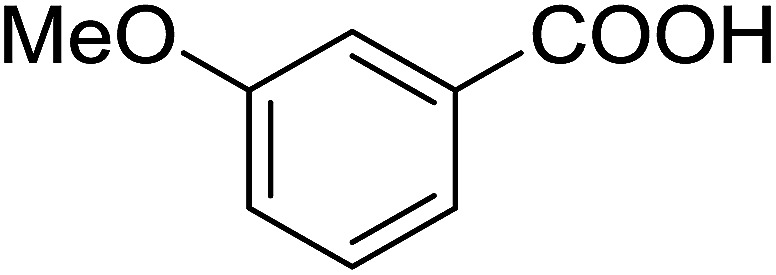	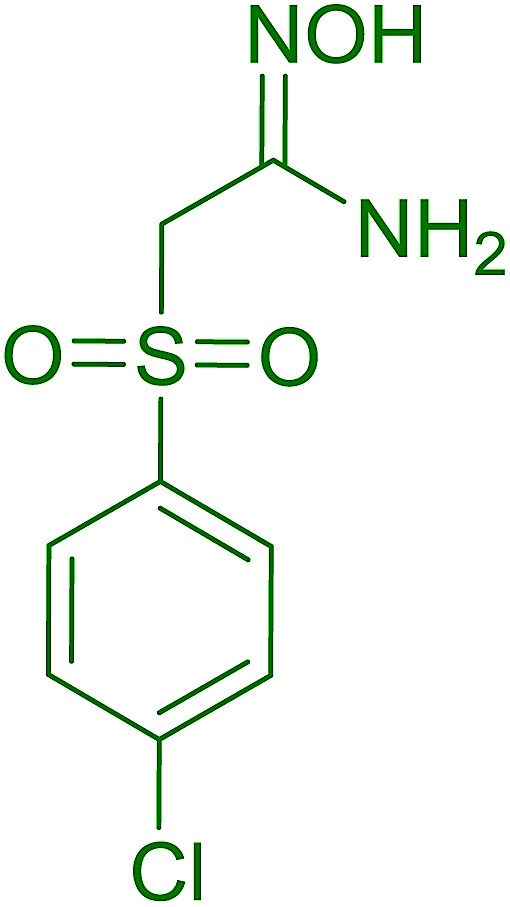	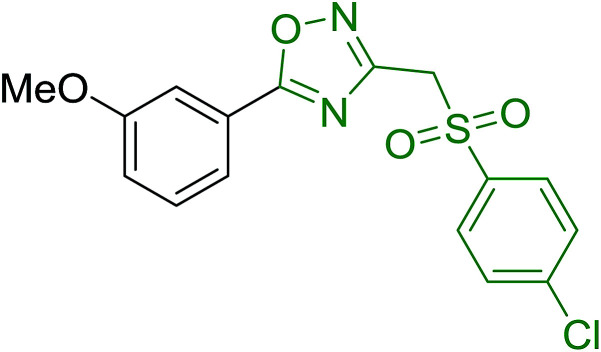	89

a Reagents and conditions: (i) PS-TPP, CCl_3_CN, carboxylic acid, THF, MW, 5 min, 100 °C; (ii) amidoxime, DIEA, THF, MW, 15 min, 150 °C; molar ratios of PS-TPP : CCl_3_CN : carboxylic acid : amidoxime : DIEA 3 : 1.5 : 1 : 1 : 2.

### 4.5. Synthesis of vinylthio-, vinylsulfinyl-, vinylsulfonyl- and vinylketo-benzofuroxans and benzofurazans

**Scheme 19:**
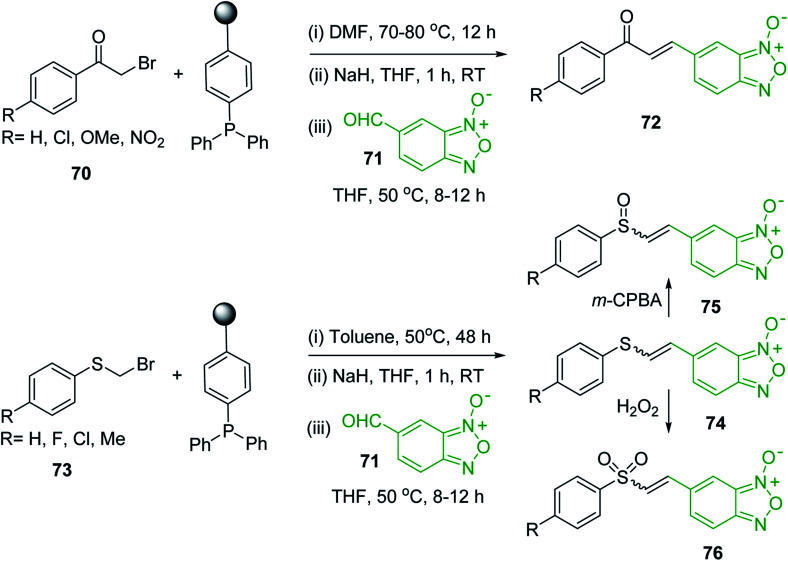
Solid-phase synthesis of vinylketo-benzofuroxanyl compounds and their vinylthio, vinylsulfinyl, and vinylfulfonyl derivatives.

Vinylthio-, vinylsulfinyl and vinylsulfonyl-benzofuroxan heterocycles possess remarkable *in vitro* activities against different *Trypanosoma cruzi* strains,^123^ which is a protozoan parasite responsible for causing Chagas' disease, a potentially life-threatening disease of the heart and gastrointestinal tract. The parasite infects a population of nearly 30 million yearly and is transmitted to animals and people by insect vectors. Unfortunately, the emergence of *T. cruzi* parasite strains resistant to drugs used clinically and experimentally as anti-*T. cruzi* increased the urgency to design novel anti-trypanosomatid agents. On the basis of previous results^123^ obtained by Castro *et al.* in regards to the trypanosomicidal properties of benzofuroxan derivatives, they developed a synthetic route to access these derivatives using a Wittig reaction performed under mild conditions with PS-TPP.^124^ Attempted preparation of such compounds though classical aldolic conditions led to total decomposition of the starting formylbenzofuroxan or reduction the *N*-oxide moiety. As shown in Scheme 19, treatment of 2-bromoacetophenones (70) with PS-TPP yielded the corresponding phosphonium salts, which upon deprotonation with NaH and subsequent condensation with formylbenzofuroxan (71) and the concomitant elimination of polymer-supported triphenylphosphine oxide through Wittig reaction produced exclusively *E*-vinylketo-benzofuroxans (72). Similarly, (bromomethyl)(phenyl)sulfane (73) were transformed in a similar manner to mixtures of separable *E*/*Z* vinylthio-benzofuroxan geometric isomers (74) (Scheme 19), which were further derivatized to the vinylsulfinyl- 75, and vinylsulfonyl-76 benzofuroxans by oxidation with *m*-CPBA and H_2_O_2_, respectively.

**Scheme 20:**
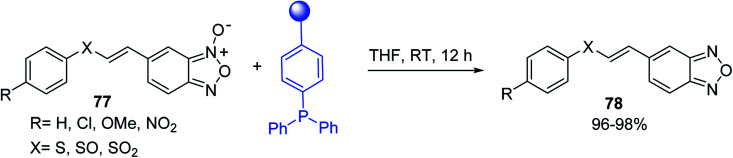
Solid-phase synthesis of benzofurozan derivatives.

In addition, PS-TPP was used as a mild reducing agent to render benzofurozan derivatives 78 in excellent yields by deoxygenating a number of the benzofuroxan products 77 for the purpose of verifying the role played by the N-oxide group in the trypanosomicidal activity (Scheme 20).

### 4.6. Synthesis of β-lactams using PS-TPP

**Scheme 21:**
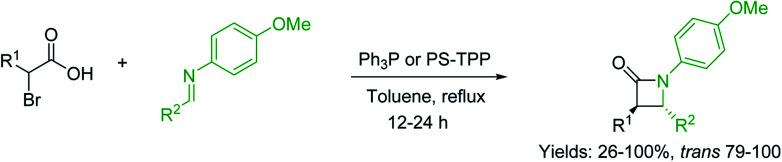
Preparation of β-lactams. Reagents and conditions: 1 : 1.2 : 2.1 molar ratios of imine : acid : phosphine, toluene, reflux, 12–24 h.

β-Lactams are the most prescribed antibacterial agents for the treatment of bacterial infections.^125,126^ However, bacteria have developed mechanisms to resist the action of β-lactam drugs. Thus, the synthesis of β-lactams with new structural features is always useful. A simple and useful method for the generation of β-lactams by reacting α-bromo carboxylic acids and imines was described by Kikuchi and Hashimoto^127^ (Scheme 21). This coupling reaction was mediated by Ph_3_P or PS-TPP to afford the β-lactams with high *trans*-selectivity and high yields in most cases.

## 5. Peptide synthesis

### 5.1. Peptide synthesis with polymeric triphenylphosphine/2,2′-dipyridyl disulfide

**Scheme 22:**
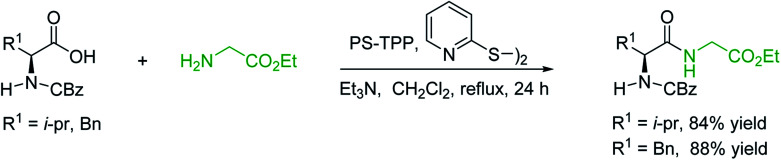
Preparation of peptides with polymeric triphenylphosphine/2,2′-dipyridyl disulfide. Reagents and conditions: 2 : 1.5 : 1 : 1 : 1 molar ratios of PS-TPP : PySSPy : amino acid : ethyl glycinate : Et_3_N, CH_2_Cl_2_, reflux, 1 d.

Horiki described the synthesis of dipeptides by the Mukaiyama procedure^128^ using PS-TPP and 2,2′-dipyridyl disulfide (Scheme 22).^129^ The procedure involved refluxing a mixture of PS-TPP, PySSPy, *N*-protected valine, ethyl glycinate, and Et_3_N in CH_2_Cl_2_ for 1 d. Best results were obtained when 2 equiv. of PS-TPP and 1.5 equiv. PySSPy to each amino acid were employed.

### 5.2. Synthesis of peptidyl ketones and peptidyl diketones from peptidyl phosphorane 4

**Scheme 23:**
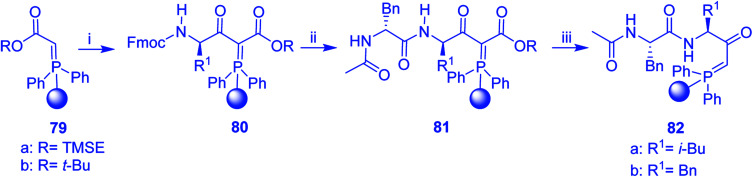
Synthesis of peptidyl phosphoranes. Reagents and conditions: (i) Fmoc-AA-OH, MSNT/2,6-lutidine, 12 h, RT for 79a; BTFFH, DIPEA, 12 h, RT for 79b; (ii) 20% piperidine/DMF, 6 min; Fmoc-AA-OH, DIC, HOBT, DMF, 3 h; (iii) TAS-F, DMF, 1 h, RT for 81a or 95% TFA, CH_2_Cl_2_, 15 h, RT for 81b.

El-Dahshan *et al.* demonstrated the utility of PS-TPP in the solid-phase synthesis of peptidyl ketones and peptidyl diketones by C-alkylations and C-acylations of peptidyl phosphorus ylide resins in which the substrate was eventually freed oxidative or by hydrolytic cleavage.^130^ The starting point for the synthetic sequence was the preparation of polymer-supported triphenylphosphoranylidene acetate (79) from PS-TPP and 2-(trimethylsilyl)ethyl 2-bromoacetate or *t*-butyl 2-bromoacetate. Subsequent acylation of 79 with Fmoc-protected amino acids (79 → 80) (Scheme 23), followed by Fmoc cleavage of 80, peptide elongation, and acylation of the *N*-terminus of the resulting peptidyl-phosphoranylidene acetate ester furnished resin 81. Deprotection of the ester group of 81 led to immediate decarboxylation to produce peptidyl phosphorane 82.

Next, methylation (entry 1) or benzylation (entry 2) of 82a (Table 43), followed by oxidative cleavage afforded the peptidal diketones (entries 1 and 2) in good yields. For the preparation of peptidyl ketones, 82b was directly hydrolyzed under basic conditions with NaHCO_3_ in THF/water (entries 3 and 4) or under acidic conditions in the case of its methylated phosphorane 82a (entry 5).

**Table 43:** Synthesis of peptidyl ketones and peptidyl diketones from phosphorane 82^a^

Entry	Peptidyl phosphorane 82	Alkylated phosphorane	Peptidyl ketones/diketones	Yield (%)
1	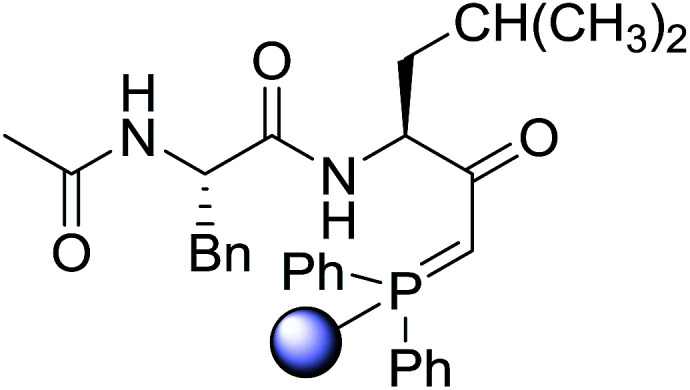	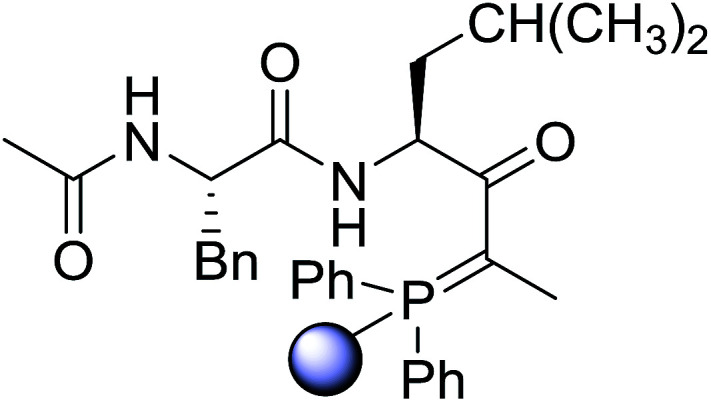	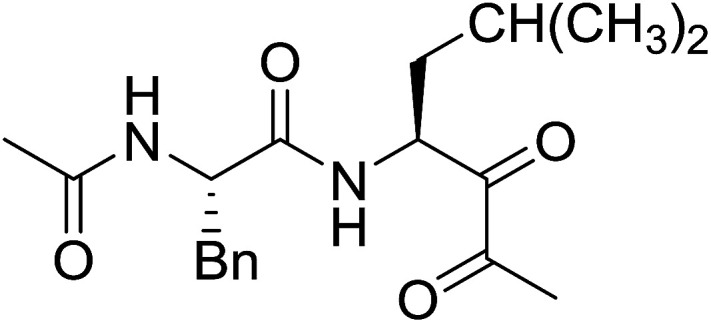	75
2	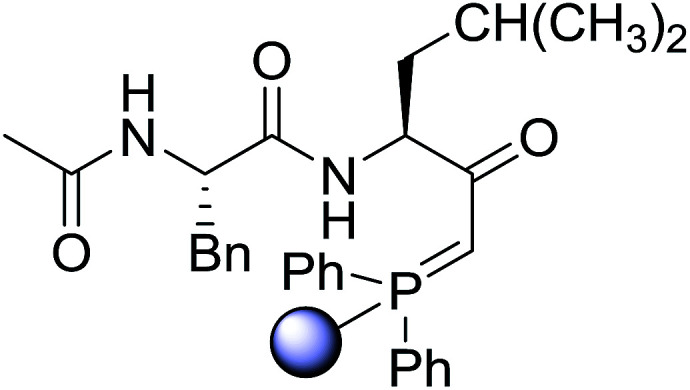	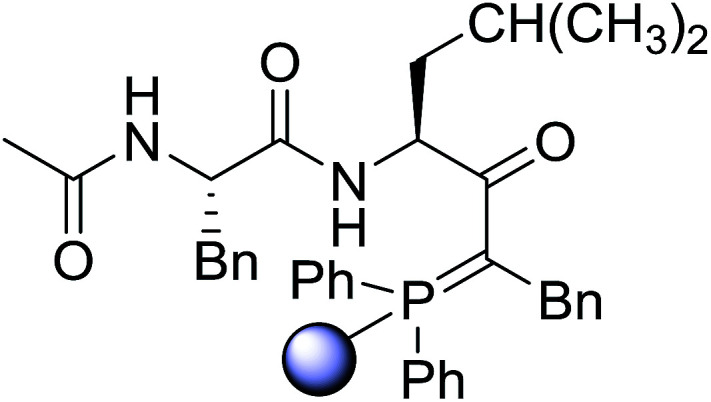	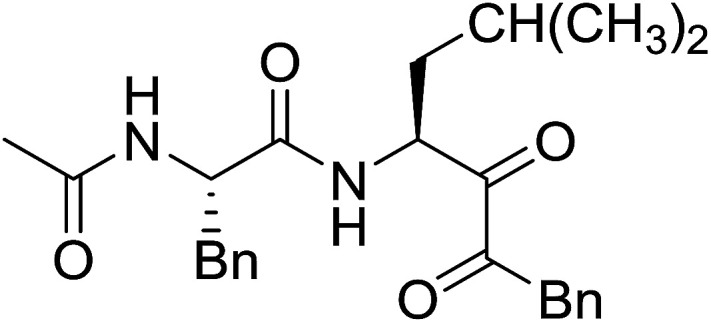	63
3	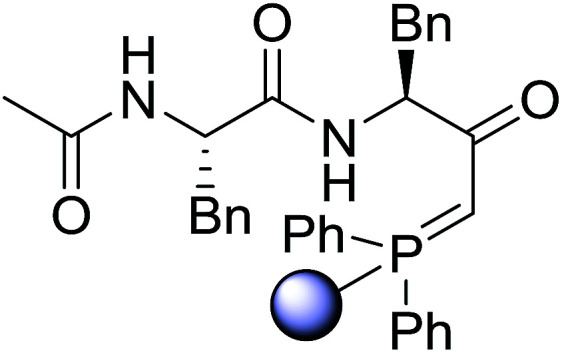	—	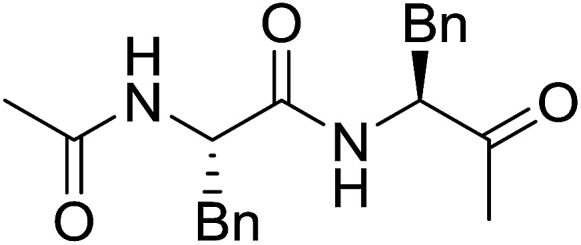	45
4	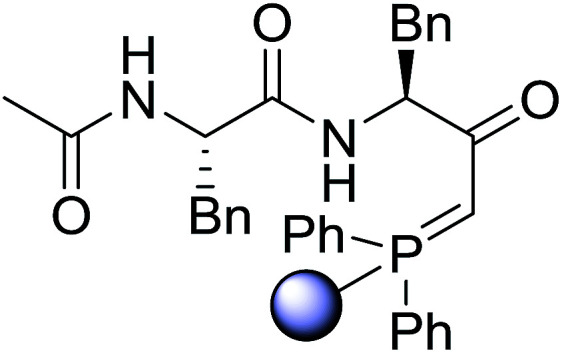	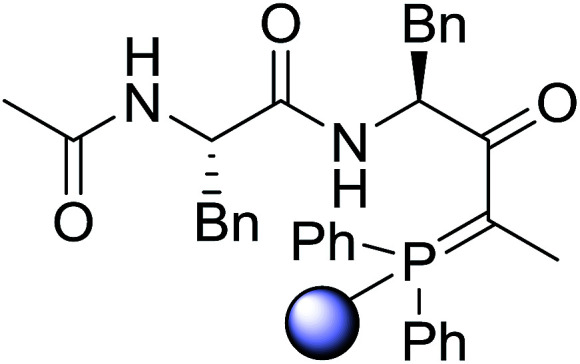	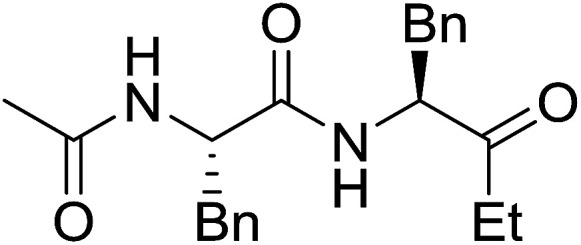	38
5	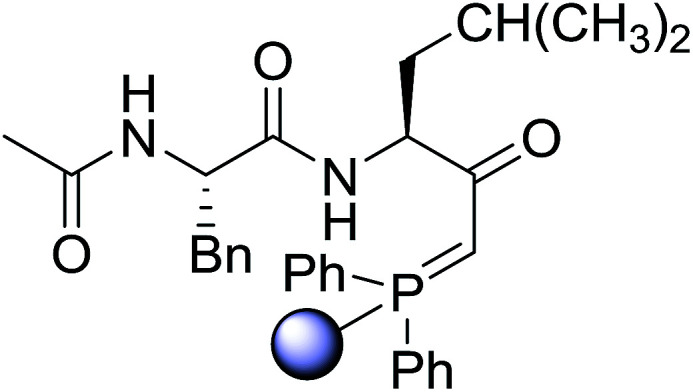	—	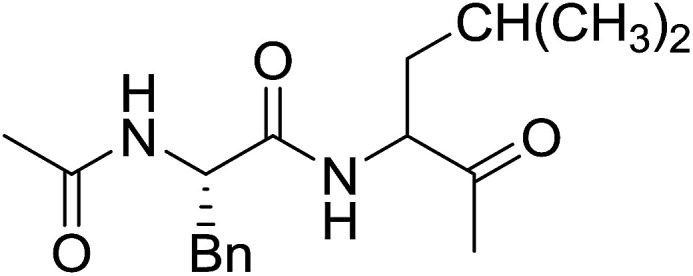	70

a Reagents and conditions: methylation of 82a and 82b: alkyl halide (3 equiv.), THF, 12 h, RT for 82a or 70 °C for 82b; oxidative cleavage conditions: 3–4 equiv. DMSO in acetone, CH_2_Cl_2_, 1 h, 0 °C; basic hydrolysis conditions: 5% NaHCO_3_, THF/H_2_O (4 : 1); acidic hydrolysis conditions: glacial acetic acid/THF/H_2_O (1 : 4 : 1), 8 h, 50 °C.

## 6. Application in total synthesis

### 6.1. Synthesis of a small library of palmarumycin CP_1_ analogs

Wipf and co-workers^132^ utilized PS-TPP for the synthesis of a small library of palmarumycin CP_1_ (83) analogs after developing an efficient synthetic approach for the preparation of palmarumycin CP_1_, a natural product and an antifungal agent.^131^ The library was obtained by a Mitsunobu coupling reaction of palmarumycin CP_1_ with a total of 13 alcohols using PS-TPP (Table 44).^132^ The reaction was performed on a very small scale (2–7 mg) to afford the ether products 84 in high purity and reasonable yields.

**Table 44:** Preparation of palmarumycin analogs^a^

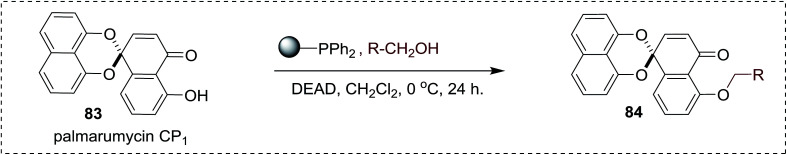			
Entry	Phenol	R	Yield (%)
1	Palmarumycin CP_1_	(*E*)-HC=CHPh	52
2		(*E*)-HC=CHMe	88
3		*n*-C_5_H_11_	50
4		(*E*)-CH_2_HC=CHEt	43
5		*m*-MeOPh	67
6		Bn	33
7		2-Furyl	29
8		(*E*,*E*)-HC=C(Me)CH_2_CH_2_CH=CMe_2_	83
9		3-Furyl	50
10		2-Pyridyl	43
11		3-Pyridyl	29
12		4-Pyridyl	17
13		HC=CH_2_	71

a Reagents and conditions: 1 : 5 : 5 : 5 molar ratios of palmarumycin CP_1_ : PS-TPP : alcohol : DEAD, CH_2_Cl_2_, 0 °C, 24 h.

### 6.2. Total synthesis of epothilone A

**Scheme 24:**
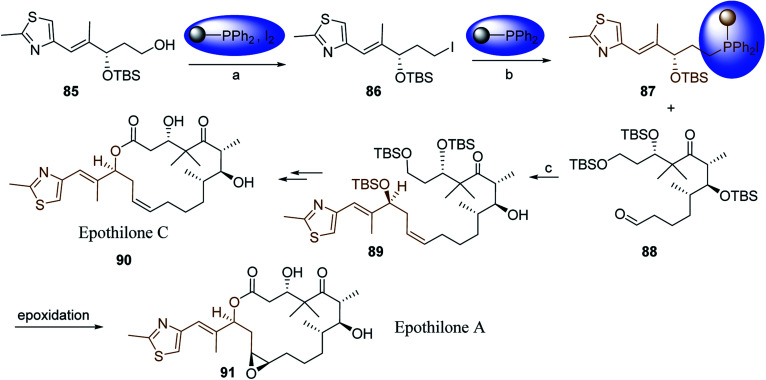
Total synthesis of epothilones A and C. Reagents and conditions: (a) iodine (4.0 equiv.), PS-TPP (5.0 equiv., 3.3 mmol g^−1^), imidazole (6.0 equiv.), MeCN/Et_2_O (3 : 1), then diethylaminomethylpolystyrene (8.0 equiv., 3.2 mmol g^−1^), Amberlite IRC-50 (13.0 equiv., ∼5 mmol g^−1^), toluene, RT, 105 min, 73%; (b) PS-TPP (1.0 equiv., 3.3 mmol g^−1^), toluene, 90 °C, 18 h; (c) phosphonium salt 87 (2.5 equiv.), NaHMDS (10.0 equiv.), THF, RT, 15 min, then THF wash, then aldehyde 88 (1.0 equiv.), 78 °C → 40 °C, 15 min, 93%.

The epothilones^133,134^ belong to a class of natural products that exhibit extraordinary cytotoxicity and are potential cancer drugs. As such, they prevent cancer cells from dividing by promoting GTP-independent tubulin polymerization, thus inducing mitotic arrest and inhibiting tumor growth.^134^ Mechanistic investigation into the mode of action showed that the epothilones bind competitively to the same sites in β-tubulin as taxol, although the former appear to have better efficacy, and milder adverse effects than taxol.^135^ Moreover, certain epothilones exhibit cytotoxic biological activity against multiple-drug-resistant cells, and are relatively more soluble in water than the analogous taxanes, making them interesting synthetic drug targets. Ley *et al.* devised a synthetic approach employing immobilized reagents, including PS-TPP, in a multistep sequence leading to epothilones A (91) and C (90) (Scheme 24).^136,137^ Their approach to the 16-membered macrocycles (90 and 91) involved a convergent stereoselective coupling of three fragments, one of which involved a Wittig reaction between the thiazole fragment 87 and the aldehyde fragment 88. Preparation of the key polymer-bound thiazole 87 involved conversion of primary alcohol 85 to iodide 86 with PS-TPP, iodine, and polymer-supported triethylamine. The resulting iodide 86 was converted to the supported phosphonium salt 87 by heating with PS-TPP in toluene. Subsequent treatment of salt 87 with NaHMDS, followed by washing with dry THF gave the salt-free ylide which was coupled *via* a Wittig reaction with aldehyde 88 to afford the required C12–C13 *cis*-alkene 89. Having this key compound in hand, the proposed synthetic strategy was successfully completed by desilylating the allylic and primary alcohols, oxidizing the latter to the carboxylic acid, followed by a Yamaguchi macrolactonisation to afford epothilones C (90). The analogue 91 was accessed through the epoxidation of 90.

## 7. PS-TPP–metal complexes and their application in synthesis

### 7.1. Cobalt immobilization on PS-TPP

**Scheme 25:**
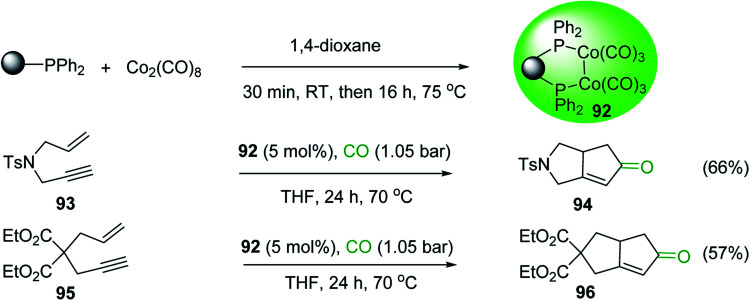
Catalytic Pauson–Khand reaction.

The Pauson–Khand (P–K) reaction was first reported in 1971^138^ and inspired many investigations in the ensuing years.^139^ The reaction is used for the synthesis of cyclopentenones *via* a cobalt carbonyl-mediated cyclization of an alkene, an alkyne, and carbon monoxide. Challenges associated with handling the very labile Co_2_(CO)_8_ spurred the development of a PS-TPP cobalt carbonyl complex as a catalyst for the Pauson–Khand reaction.^140^ The catalyst (92) was prepared by reacting equimolar amounts of PS-TPP and Co_2_(CO)_8_ in 1,4-dioxane at 75 °C and proved more stable than the latter. Thus, at 70 °C, 50 mbar overpressure of carbon monoxide, and 5 mol% of 92, the enynes 93 and 95 shown in Scheme 25 converted to the corresponding cyclopentanones 94 and 96 in 66 and 57% yield, respectively.

### 7.2. Immobilization of functionalized alkynes on PS-TPP

**Scheme 26:**
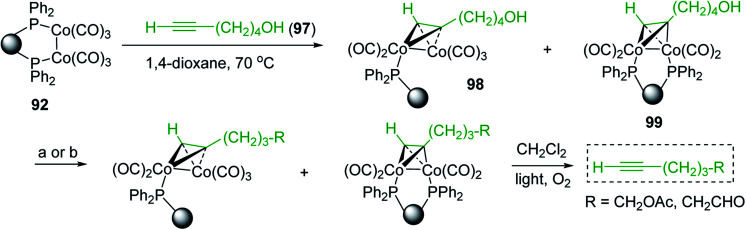
Reactions and decomplexation of immobilised functionalized alkynes. Reagents and conditions: (a) acetic anhydride, Et_3_N; (b) SO_3_-py, DMSO, Et_3_N.

The PS-TPP cobalt carbonyl complex 92 developed by Comely *et al.* was also used as a traceless alkyne linker.^141^ The temporary and reversible immobilisation of an alkyne functional group (Nicholas reaction) onto a polymeric support which leaves minimal remnant of the solid support upon decomplexation of the product is a very desirable property for solid-phase synthesis. Thus, treatment of resin 92 with hex-5-yn-ol (97) generated the polymer-bound alkyne complexes 98 and 99 (ratio; 3 : 7, respectively), which could be acetylated (70% yield) or converted to the aldehyde by the Parikh–Doering oxidation (17% yield) (Scheme 26). Decomplexation of the alkyne was possible with aerial oxidation in dichloromethane under white light for 72 h.

### 7.3. PS-TPP-supported rhodium catalyst for use in the synthesis of ketones from benzyl alcohols and 1-alkene

Jun *et al.* reported the reaction benzyl alcohol bearing various substituents with 1-alkene to generate the corresponding ketones by PS-TPP-supported rhodium catalyst formed *in situ*.^142^ In a typical experiment, the alcohol reacted with an equimolar amount of 1-alkene in the presence of PS-TPP (15 mol%), Ph_3_P (5 mol%), RhCl_3_·*x*H_2_O (5 mol%), and 2-amino-4-picoline (100 mol%) as co-catalyst (Table 45) in toluene at 130 °C for 72 h. The catalytic activity of the recovered catalyst did not decrease after reusing it for four cycles and electron-withdrawing substituents (entries 2 and 4) afforded ketones in higher yields than electron-donating groups (entries 3 and 5). The yield was also negatively impacted with sterically hindered olefinic substrates (entries 6 and 7). This polymer-supported Rh(i) catalyst could also be generated *in situ* from other Rh(i) complexes *via* a ligand exchange reaction.

**Table 45:** Preparation of ketones from benzyl alcohols and 1-alkene

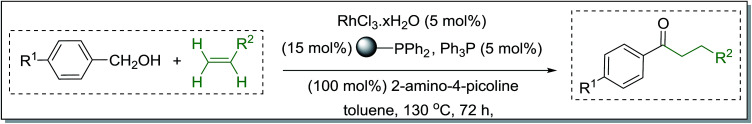			
Entry	R^1^	R^2^	Yield (%)
1	H	*n*-C_4_H_9_	69
2	F	*n*-C_4_H_9_	75
3	CH_3_	*n*-C_4_H_9_	67
4	CF_3_	*n*-C_4_H_9_	70
5	CH_3_O	*n*-C_4_H_9_	61
6	H	*t*-C_4_H_9_	50
7	F	*t*-C_4_H_9_	53

### 7.4. PS-TPP-supported ruthenium catalyst for use in transfer hydrogenation and hydrocarbon oxidation

Chloro-ruthenium complexes such as RuCl_2_(PPh_3_)_3_ (100) have been used frequently in metal-mediated transformations which include transfer hydrogenation and hydrocarbon oxidation.^143^ Soluble phosphines have been widely used as ligands for catalysis employing heavy metals. Unfortunately, removal of soluble derivatives of heavy metals from organic reactions is challenging. Besides providing ease of separation from the product mixture at the end of the reaction, attaching a metal complex to a polymer lowers toxicity and air sensitivity of such species and precludes contamination of the product with heavy metals. Leadbeater reported an insoluble version of RuCl_2_(PPh_3_)_3_ (101) and described its capabilities in transfer hydrogenation and hydrocarbon oxidation. Immobilization of the ruthenium complex was carried out by stirring an equimolar mixture of PS-TPP and RuCl_2_(PPh_3_)_3_ in toluene at room temperature overnight.^144^ The resulting polymer-bound ruthenium complex, which was isolated by filtration, was stable in air and amenable to prolonged storage under an atmosphere of nitrogen. Using catalytic amount of the immobilized complex (0.15%), selective hydrogen transfer from 2-propanol to various ketones (Table 46, entries 1–4) occurred rapidly, delivering the product alcohols in yields (40–83%) comparable to those obtained with the unsupported RuCl_2_(PPh_3_)_3_ complex. Similarly, the oxidation of a range of alcohols (entries 5 and 6) and hydrocarbons (entries 7–10) using a catalytic amount of the polymer-supported ruthenium complex and *t*-BuOOH gave the desired ketones in good yields (47–100%). It was shown that the catalyst could be recycled and reused a number of times without losing activity.

**Table 46:** Transfer hydrogenation and hydrocarbon oxidation with PS-TPP–RuCl_2_(PPh_3_)_2_ complex 101^a^

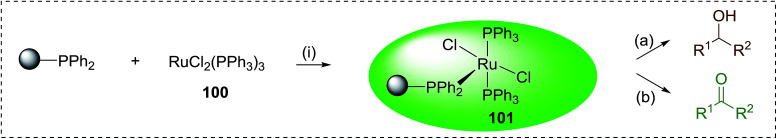							
Entry	Substrate	Product	Yield (%)	Entry	Substrate	Product	Yield (%)
1	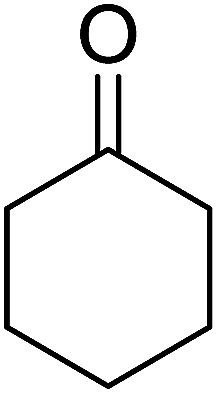	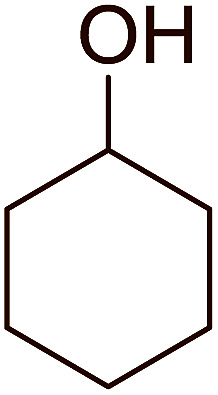	83	6	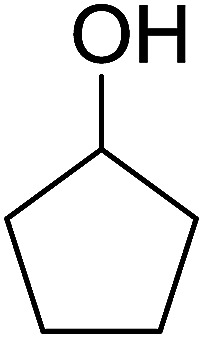	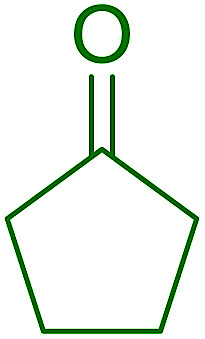	64
2	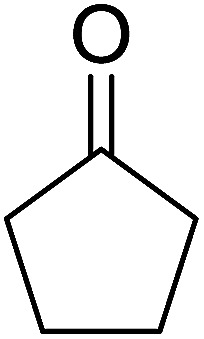	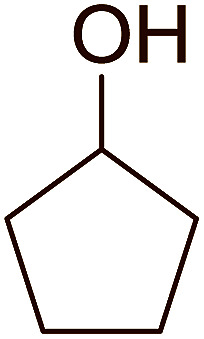	62	7	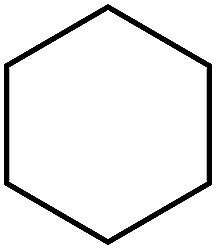	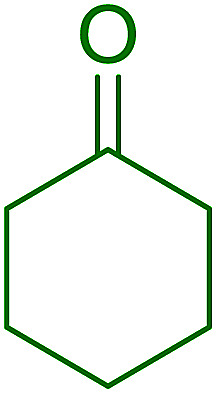	45
3	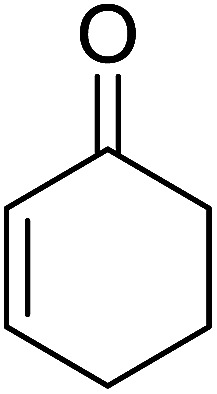	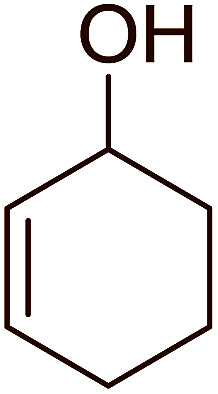	61	8	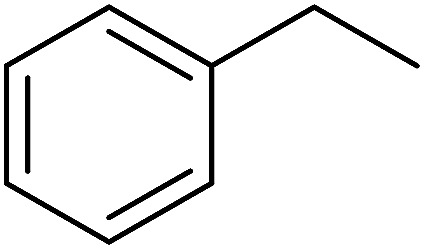	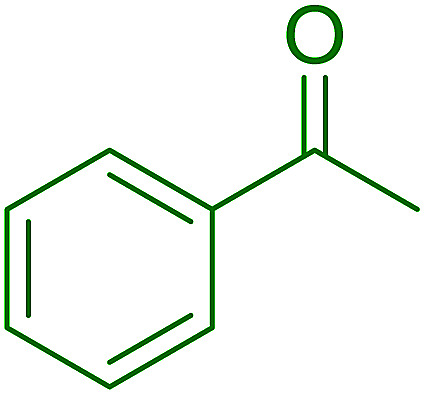	80
4	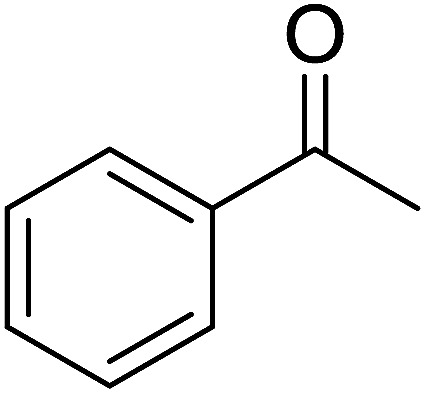	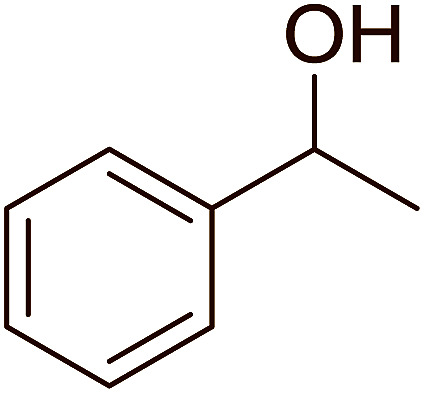	40	9	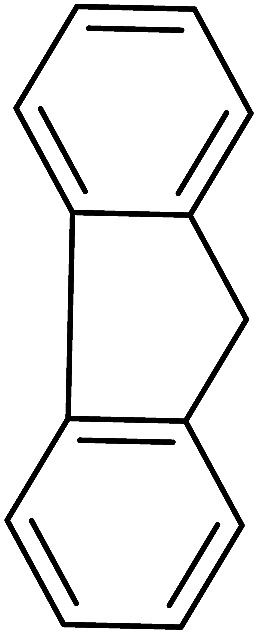	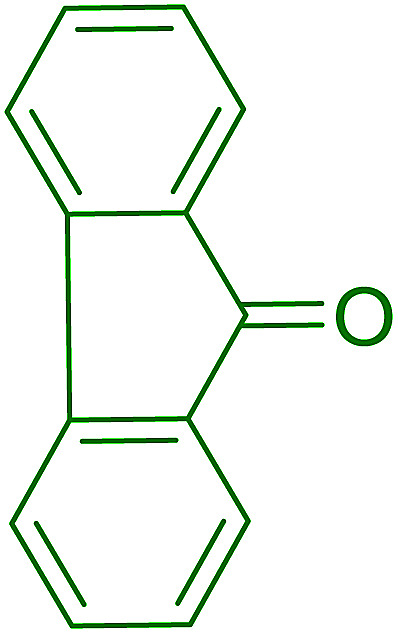	70
5	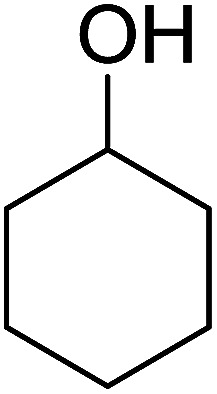	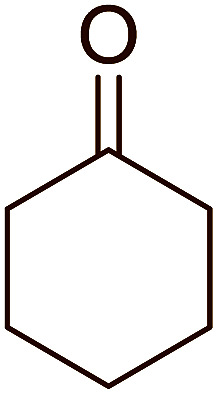	85	10	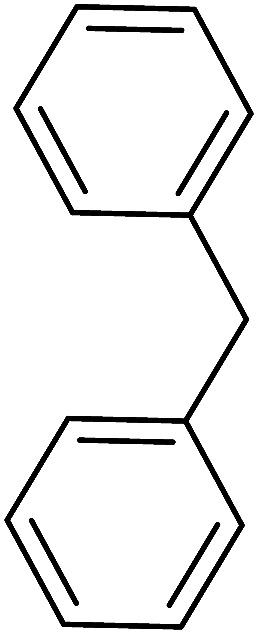	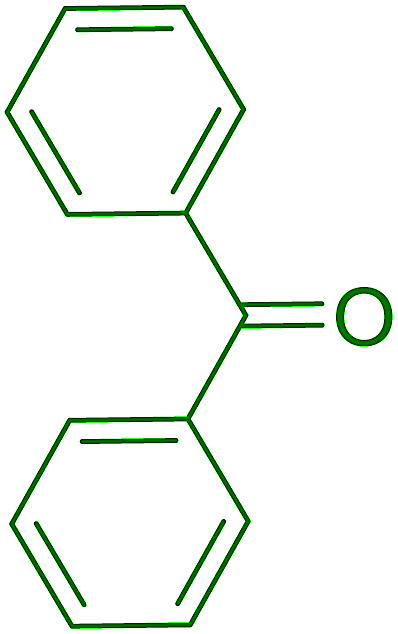	89

a Reagents and conditions: (i) 1 : 1 molar ratio of PS-TPP : RuCl_2_(PPh_3_)_3_, toluene, RT, overnight; entries 1–4, path a, 50 mg : 10 mL : 10 mmol reagent ratios for PS-TPP–RuCl_2_(PPh_3_)_2_ complex : 2-propanol : ketone, 1,2-dichloroethane, reflux, 15 min, then NaOH (10 mg), 2-propanol, reflux, 1.5 h; entries 5–10, path b, 50 mg : 2 mmol : 6 mmol reagent ratios for PS-TPP–RuCl_2_(PPh_3_)_2_ complex : hydrocarbon : 30% peracetic acid, 1,2-dichloroethane/EtOAc (7 : 1), reflux, 2 h.

### 7.5. PS-TPP–arene-ruthenium complex for use in enol formate synthesis and the cyclopropanation of olefins

**Scheme 27:**
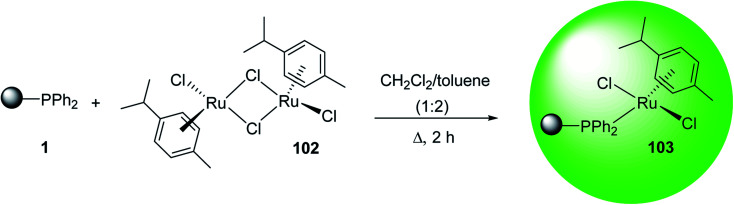
Preparation of PS-TPP–arene-ruthenium complex 103.

Arene-ruthenium complex 103 is another analog of a homogeneous metal complex that has been immobilized on PS-TPP by Leadbeater.^145^ The free complex [Ru-(*p*-cumene)Cl_2_(Ph_3_P)] is among many other arene ruthenium catalysts that have been frequently used in a plethora of metal-mediated reactions such as enol formate synthesis^146^ and cyclopropanation of alkenes.^147^ The air-stable polymer-bound complex 103 was prepared *via* a thermolysis reaction of the dimer 102 with two molar equivalents of PS-TPP (1) in CH_2_Cl_2_ (Scheme 27).

The ruthenium polymer-supported complex 103 was used catalytically (1 mol%) for the regioselective addition of formic acid to terminal alkynes and diynes to generate the corresponding enol formates in 76–95% yields (Table 47, entries 1–5). Reactions were conducted in toluene since it produced optimum yields even though resin swelling is not as marked as in other solvents. The catalyst was similarly active in alkene cyclopropanation reactions (entries 6 and 7).

**Table 47:** Enol formate synthesis and alkene cyclopropanation with [Ru-(*p*-cumene)Cl_2_(PS-TPP)]^a^

Entry	Substrate 1	Substrate 2	Product	Yield (%)
1	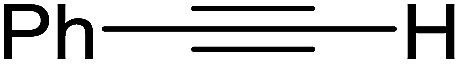	HCO_2_H	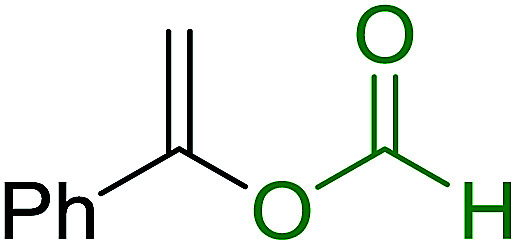	76
2	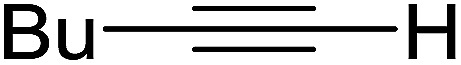	HCO_2_H	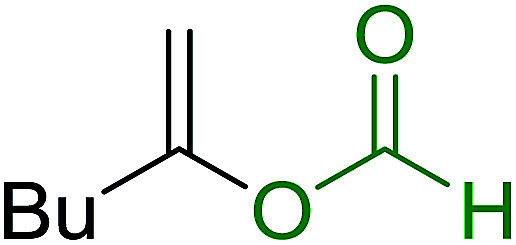	95
3	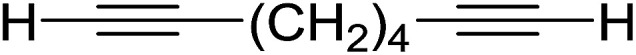	HCO_2_H	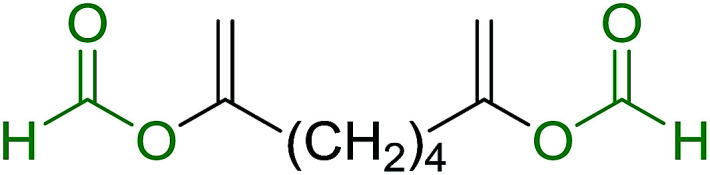	81
4	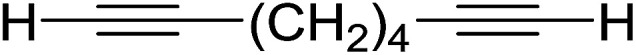	MeCO_2_H	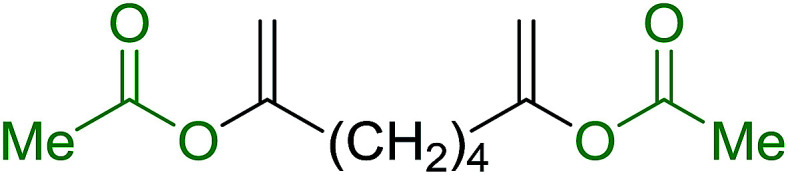	82
5	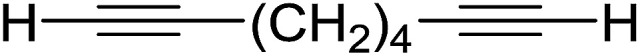	PhCO_2_H	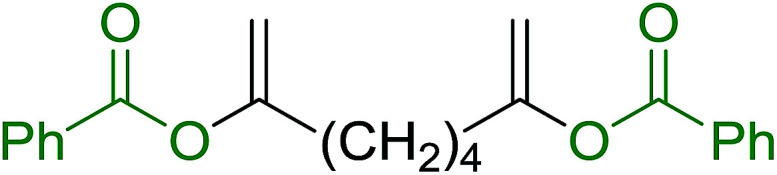	84
6	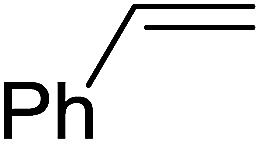	N_2_CHCO_2_Et	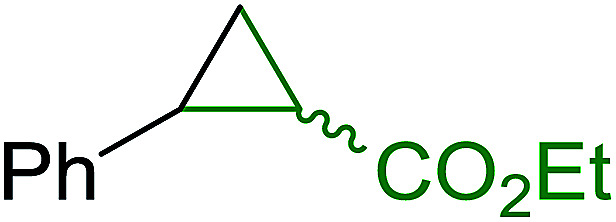	71
7	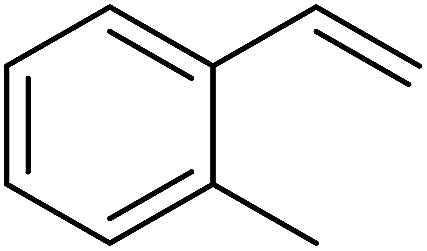	N_2_CHCO_2_Et	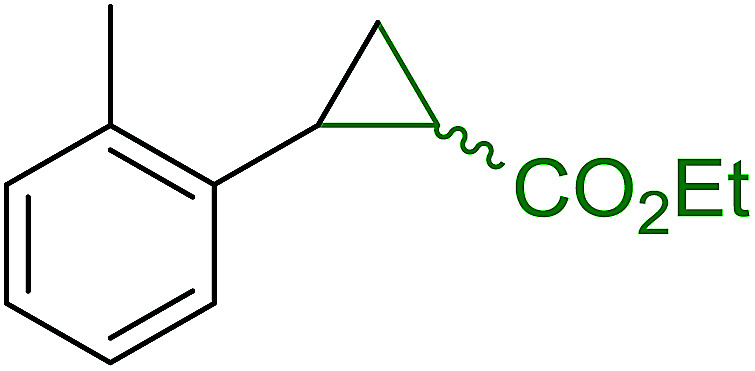	82

a Reagents and conditions: entries 1–5, 1 : 1.3 : 0.01 molar ratio of formic acid : alkyne : [Ru-(*p*-cumene)Cl_2_(PS-TPP)], toluene, reflux, 15 h; entries 6 and 7, 1 : 1.3 : 0.01 molar ratio of ethyl diazoacetate : alkene : [Ru-(*p*-cumene)Cl_2_(PS-TPP)], CH_2_Cl_2_, 60 °C, 4 h.

### 7.6. Catalytic oxidation of benzylic and allylic alcohols and acid anhydride synthesis with PS-TPP–cobalt complex

PS-TPP has also been used by Leadbeater to attach the homogeneous transition metal catalyst CoCl_2_(PPh_3_)_2_ (104) and assess its ability in the catalytic oxidation of alcohols to aldehydes and ketones and the synthesis of acid anhydrides (Table 48). Immobilization of the cobalt catalyst was achieved by agitating a mixture of CoCl_2_(PPh_3_)_2_ and the functionalized phosphine resin 1 (1 : 1.7 molar ratio) in CH_2_Cl_2_ overnight.^148^ The catalyst loading was estimated to be approximately 2.4 mmol g^−1^ of resin. The resulting complex 105 (1 mol%) was used to selectively oxidize primary and secondary benzylic alcohols as well as allylic alcohols using *tert*-butylhydroperoxide as oxidant (entries 1–4). Aliphatic alcohols remained unaffected under the reaction conditions. The group demonstrated that the polymeric support had little effect on the product yield compared to the homogeneous cobalt complex. The supported catalyst was also investigated in the coupling of acid chlorides and carboxylic acids. Several asymmetrically substituted acid anhydrides were prepared in good yields (entries 5–7).

**Table 48:** Catalytic oxidation of alcohols and acid anhydride preparation^a^

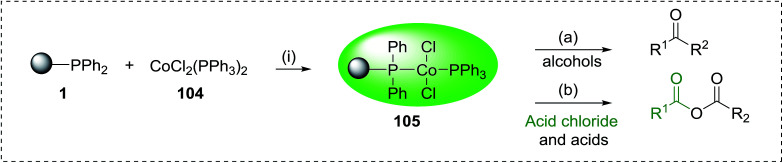			
Entry	Substrate	Product	Yield (%)
1	BnOH	PhCHO	86
2	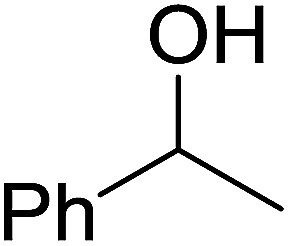	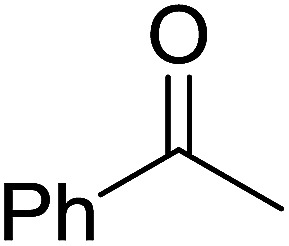	91
3	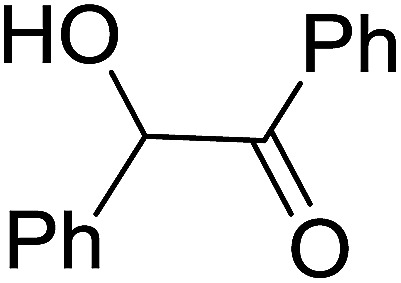	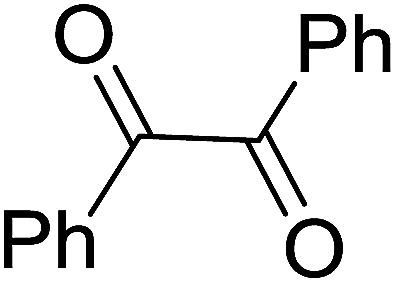	75
4	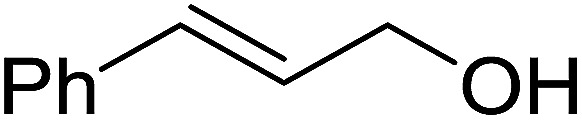	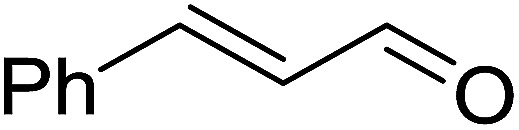	70
5	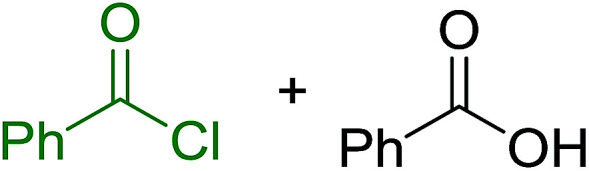	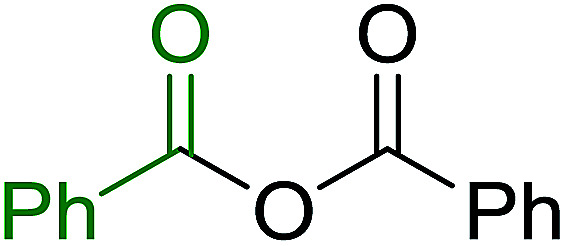	87
6	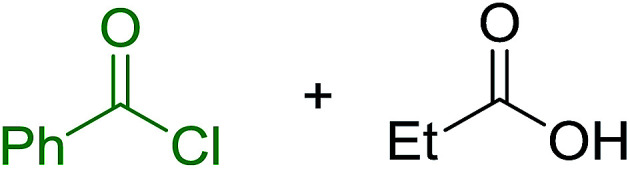	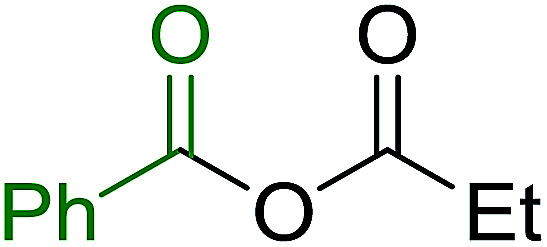	78
7	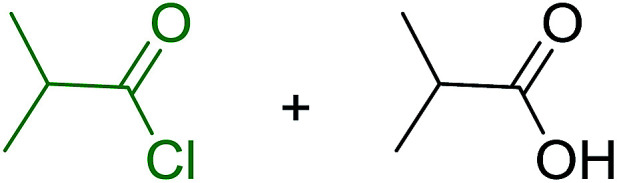	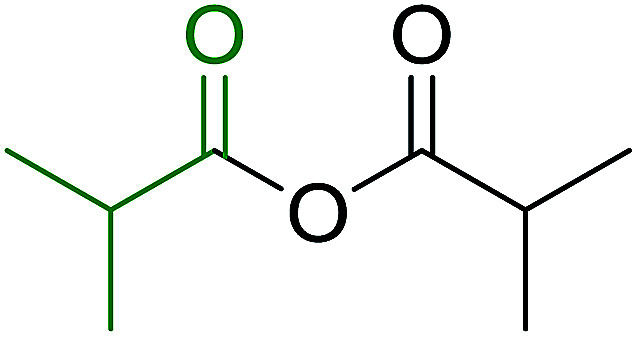	86

a Reagents and conditions: (i) 1.7 : 1 molar ratio of PS-TPP : CoCl_2_(PPh_3_)_2_, CH_2_Cl_2_, RT, overnight; entries 1–4, path a, 1 : 2 : 0.01 molar ratio of alcohol : *tert*-butylhydroperoxide : PS-TPP–CoCl_2_(PPh_3_), CH_2_Cl_2_, reflux, 4 h.; entries 5–7, path b, 1 : 1 : 0.01 molar ratio of acid chloride : carboxylic acid : PS-TPP–CoCl_2_(PPh_3_), CH_2_Cl_2_, 40 °C, 4 h.

### 7.7. Polystyrene-bound triphenylphosphine gold(i) catalysts; synthesis of furans, pyrroles, and oxazolidines

Cationic gold(i) complexes exhibit significant affinity to carbon–carbon multiple bonds and have become particularly attractive catalysts because they are resistant to moisture and air. Additionally, they display a high catalytic reactivity, often requiring a small loading to drive a wide range of transformations, such as nucleophilic reactions and intramolecular cyclizations.^149^ Akai *et al.* described the synthesis of polystyrene-bound triphenylphosphine-gold(i) cationic complexes by first treating PS-TPP with (Me_2_S)AuCl (1 : 1) in CH_2_Cl_2_ at room temperature to give the immobilized gold compound PS-PPh_2_AuCl.^150^ The polymer resin mesh size was critical where the most effective coordination was achieved with mesh size 100–200. Subsequent addition of 1 molar equivalent of AgOTf or AgNTf_2_ to PS-PPh_2_AuCl generated the cationic catalysts PS-PPh_2_Au^+^OTf^−^ and PS-PPh_2_Au^+^NTf^−^ (106), respectively. The two cationic catalysts were used in the rapid synthesis of furans and pyrroles 108*via* the intramolecular cyclization of unsaturated 1,2-diols or 1,2-aminoalcohols 107. Table 49 shows some representative cyclization products obtained with catalyst 106, where similar yields were also observed when PS-PPh_2_Au^+^OTf^−^ was used.

**Table 49:** Synthesis of furans and pyrroles using PS-PPh_2_Au^+^NTf^−^ complex^a^

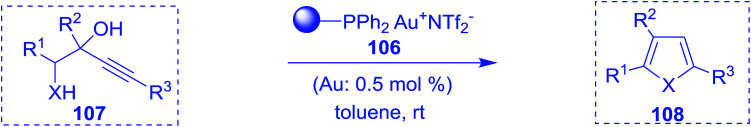					
Entry	R^1^	R^2^	R^3^	X	Yield (%)
1	H	Me	(CH_2_)_2_Ph	O	98
2	H	Me	2-Thienyl	O	88
3	–(CH_2_)_4_–		Ph	O	95
4	H	H	Ph	O	92
5	H	Me	(CH_2_)_2_Ph	NBoc	79

a Reagents and conditions: 1 : 0.005 molar ratio of PS-PPh_2_Au^+^NTf^−^ : substrate, toluene, RT, 2 h.

**Scheme 28:**
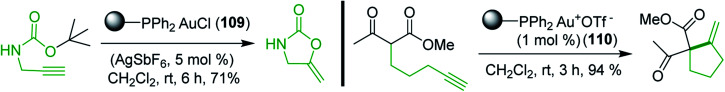
Au-catalyzed carbocycle and oxazolidine-forming reactions.

Other types of Au-catalyzed cyclizations such as carbocycle and oxazolidine-forming reactions also proceeded successfully with PS-PPh_2_Au^+^NTf^−^ (110) and the polymer-bound pre-catalyst PS-PPh_2_AuCl (109) (Scheme 28).

### 7.8. Polymer-supported monodentate phosphine complexes

#### 7.8.1. Trost's PS-PPh_2_-Pd(PPh_3_)_3_ catalyst; allylic acetates substitution

**Scheme 29:**
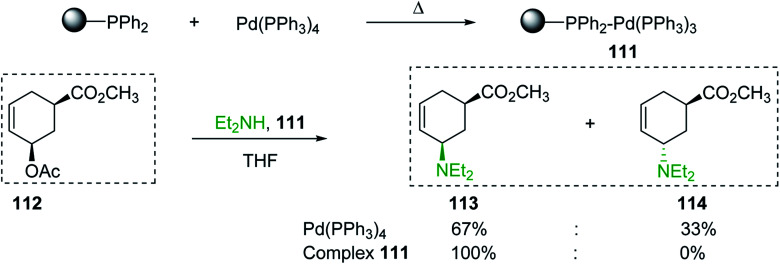
Trost's PS-PPh_2_-Pd(PPh_3_)_3_ catalyst; stereospecific amination of allyl acetates.

Palladium complexes have been used as catalysts in cross coupling reactions to affect valuable C–C bond-forming transformations. Pittman *et al.* were first to attach palladium complexes to PS-TPP and use them in the oligomerization reaction of 1,3-butadiene to give mixture of products.^151^ Keinan and Trost were the first to report the use of the polymer-supported analogue, 111, of the well-known palladium complex Pd(PPh_3_)_4_, in clean cross coupling reactions.^152^ The protocol involves heating a solution of the latter parent metal complex with PS-PPh_2_ (1) to generate complex 111 containing between 1.5–2 mol% Pd (Scheme 29). Since it was reported in the 1970's, 111 has found many useful applications in catalysis, although displaying differing selectivity in certain cases compared to the homogeneous analogue Pd(PPh_3_)_4_. For instance, when complex 111 was used as a catalyst for the reaction of the allylic acetate *cis*-3-acetoxy-5-carbomethoxy-1-cyclohexene (112) with Et_2_NH, the *cis* substitution product 109 was produced with net retention of stereochemistry due to steric steering (Scheme 29). However, the use of non-supported Pd(PPh_3_)_4_ afforded a mixture of *cis*-3-diethylamino-5-carbomethoxy-l-cyclohexene (113) and its *trans* diastereomer 114 which had inverted configuration. Apparently, the pathway leading to inversion of configuration was not possible due to the inability of the amine nucleophile to co-ordinate to the polymer supported palladium complex 111. Trost exploited the availability of this clean retention pathway for nitrogen nucleophiles for the development of a convenient isoquinuclidine synthesis.^152^

#### 7.8.2. Hallberg ‘s PS-PPh_2_-Pd(Cl)_2_PPh_3_ catalyst; Heck arylation

**Scheme 30:**
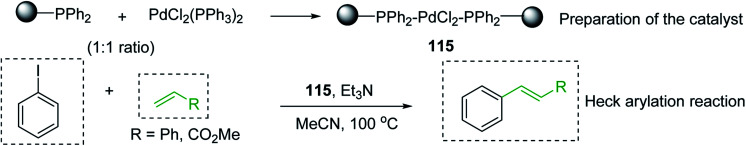
Hallberg's PS-PPh_2_-Pd(Cl)_2_PPh_3_ catalyst and its application in the Heck arylation.

Hallberg and co-workers^153^ have reported supported analogues of PdCl_2_(PPh_3_)_2_ from PS-PPh_2_ and PdCl_2_(PhCN)_2_ by reacting the two components (Scheme 30). Interestingly, the group prepared supported complexes with Pd : P ratios of 1 : 1, 1 : 2, 1 : 3 and 1 : 4 and discovered that coordination of the metal to the resin changes with metal loading, complex 115 being formed at low metal loading. The complexes were investigated in the Heck arylation of methyl acrylate and styrene with iodo- and bromobenzene (Scheme 30). The supported complex 115 with low PS-PPh_2_ : Pd ratio was found to be most effective for the arylation reactions of the aryl iodide substrates, whereas the complexes with high PS-PPh_2_ : Pd ratios worked best with the aryl bromoanalogues. In general, the rates of reaction of the latter analogues were reported to be much slower than those obtained with homogeneous analogues, reaction durations being days rather than hours.

#### 7.8.3. Palladium-catalyzed cyanation of aryl triflates and aryl halides using polymer-supported triphenylphosphine

A method for the expeditious cyanation of aryl triflates and aryl halides using a heterogeneous palladium catalyst prepared from PS-TPP as the ligand and palladium(ii) acetate has been demonstrated by Srivastava *et al.*^154,155^ In this methodology, a variety of aryl triflates and aryl halides bearing both electron-withdrawing and electron-donating groups were successfully cross-coupled with zinc cyanide under microwave as well as under conventional heating conditions (Table 50). While both reaction conditions offered similar yields for aryl triflates (91–98%), microwave-induced cyanation required much shorter times (2–3 minutes) for the completion of reaction whereas thermal conditions needed 1.5–3 hours. On the other hand, aryl halides required relatively longer microwave heating (30–50 min) time for complete reaction. It is noted that complete conversion of the triflate to the corresponding nitrile product was possible with as little as 3 mol% of PS-TPP catalyst and 1 equivalent of Zn(CN)_2_. This is noteworthy since similar transformations require much higher loading of both catalyst and cyanation reagent. By comparison, aryl halides required 7 mol% of PS-TPP catalyst.

**Table 50:** Cyanation of aryl triflates and aryl halides^a^

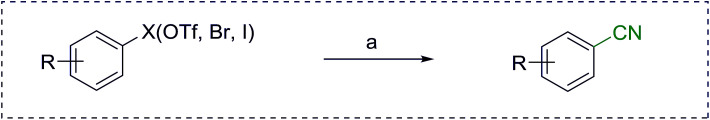				
Entry	Aryl triflate or halide	Aryl nitrile	Yield (%), microwave irradiation	Yield (%), thermal heating
1	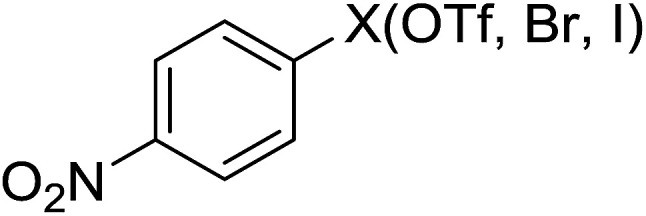	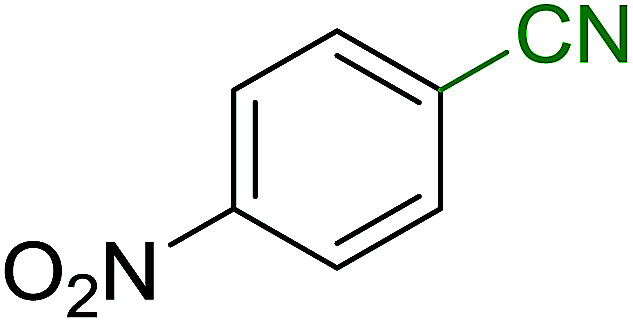	X = OTf: 92	X = OTf: 94
			X = I: 99	—
			X = Br: 95	—
2	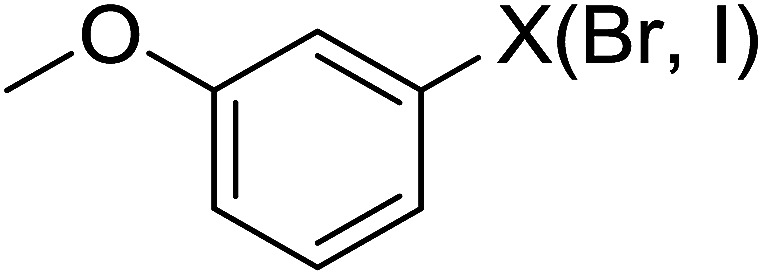	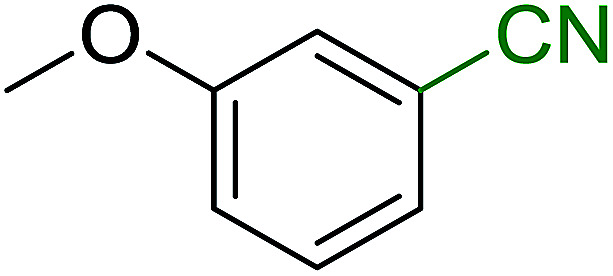	X = I: 92	—
			X = Br: 98	—
3	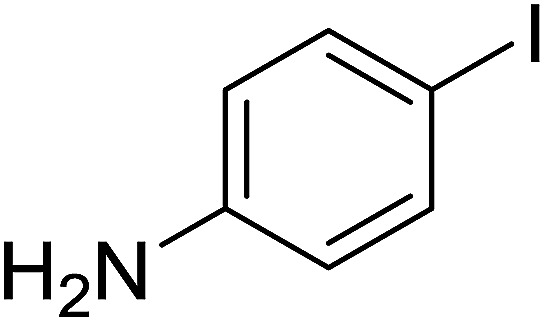	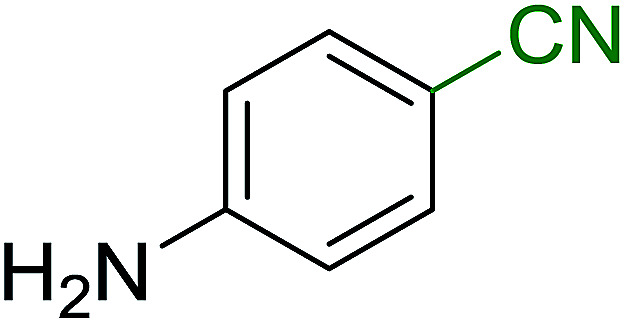	92	—
4	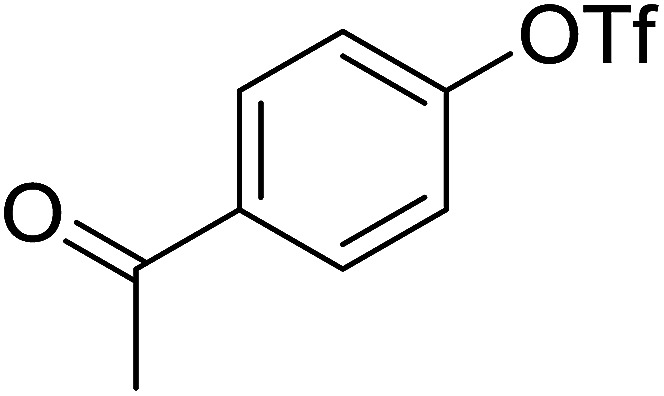	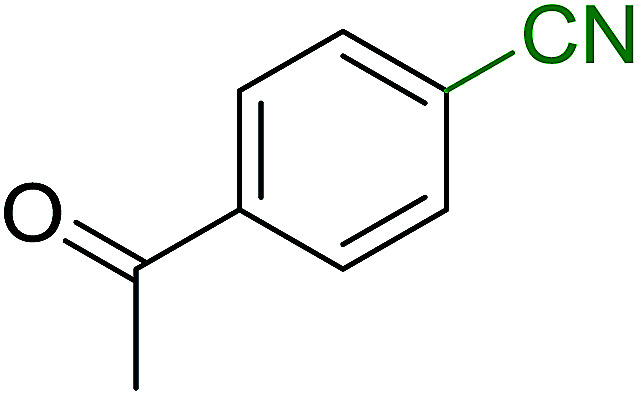	96	98
5	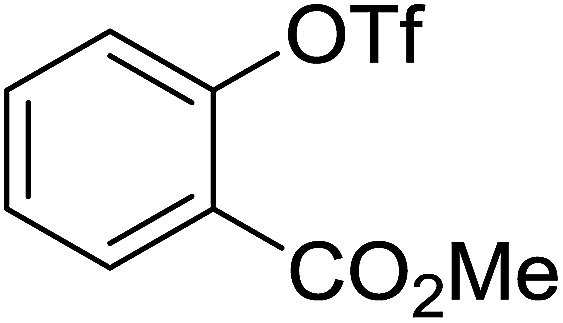	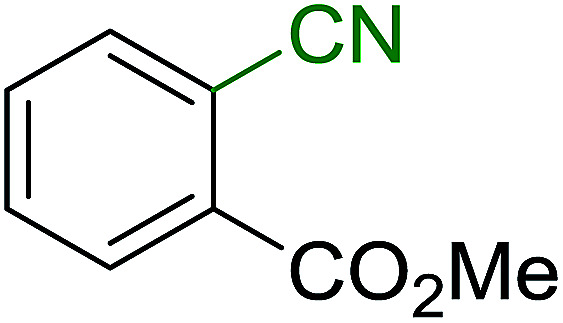	94	93
6	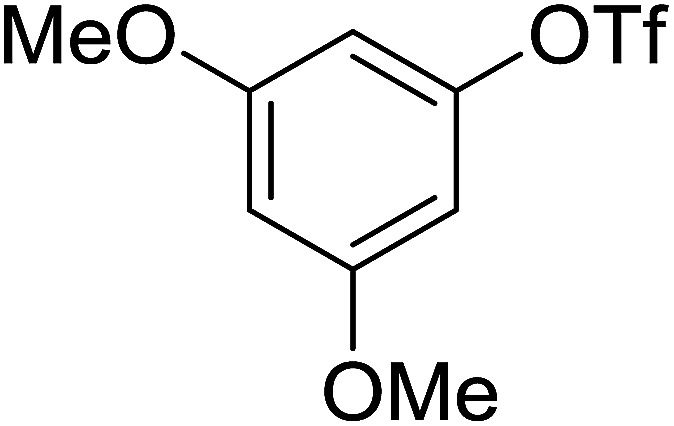	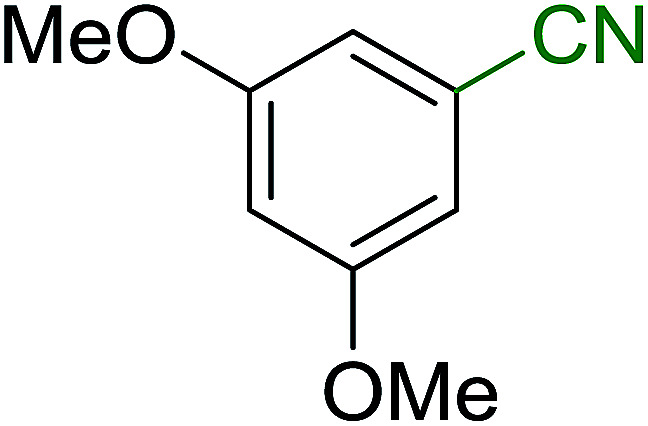	92	92
7	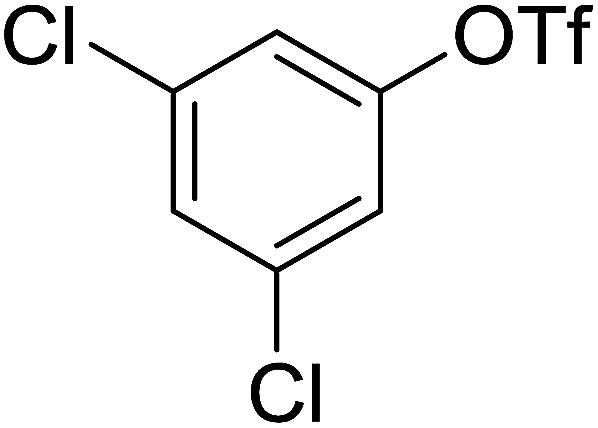	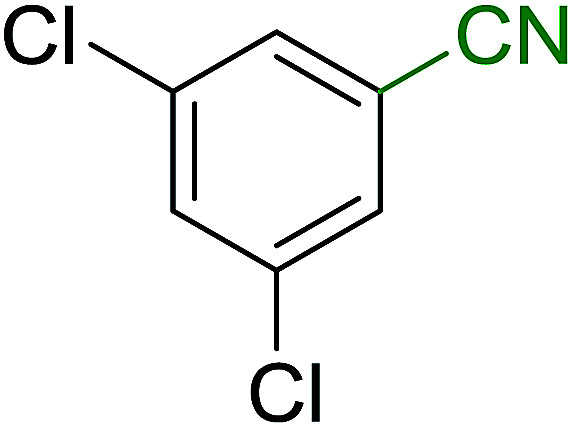	94	93
8	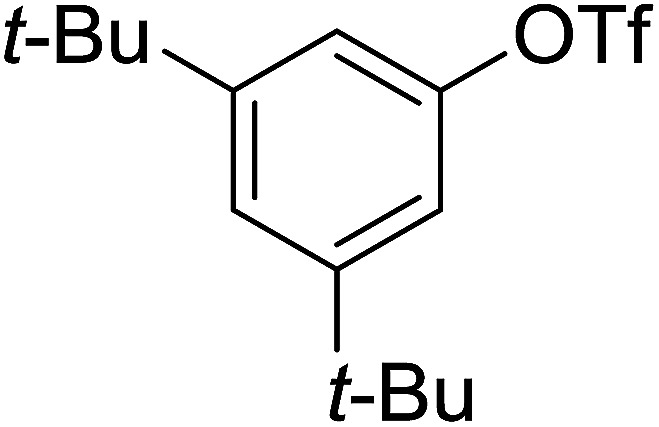	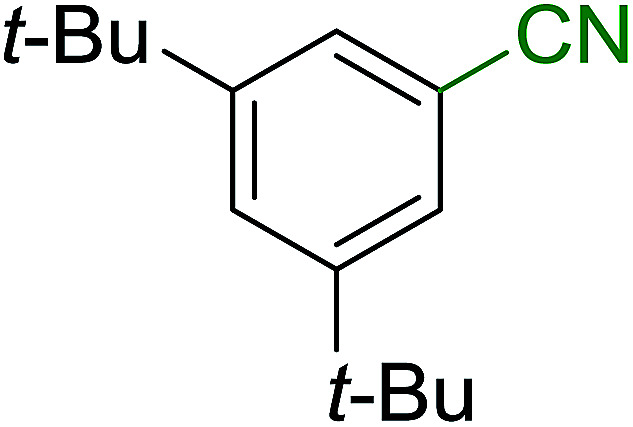	89	93
9	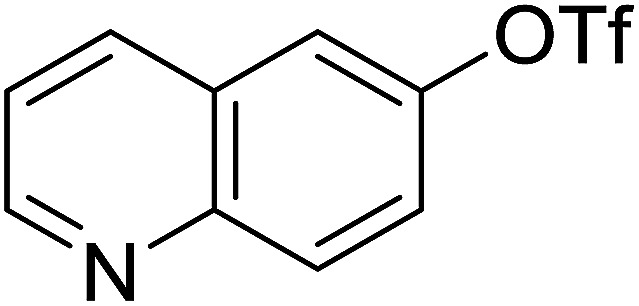	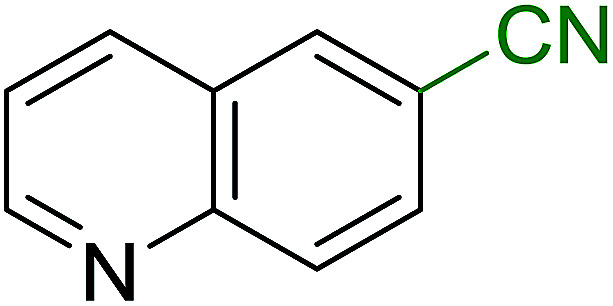	90	91

a Reagents and conditions: PS-Ph_3_P (3 mol%), Pd(OAc)_2_ (7 mol%), Zn(CN)_2_ (1 molar equivalent), DMF, microwave irradiation (2–3 min for aryl triflates; 30–50 min for aryl halides at 140 °C); thermal heating conditions (1.5–3 h).

#### 7.8.4. Allylation of carboxylic acids, alcohols, and amines using PS-TPP–palladium complex

Allyl esters, allyl ethers, and allyl amines are useful functional groups with wide utility in organic synthesis.^156^ Bhanage *et al.* reported an effective heterogeneous catalytic methodology for the allylation of various *N*- and *O*-pronucleophiles with 1-phenyl-1-propyne as the allylating agent and PS-TPP–Pd as the heterogeneous catalyst (Table 51).^157,158^ Preparation of the PS-TPP–Pd complex involved refluxing a mixture of PS-TPP (5 molar equivalents) as the heterogeneous ligand and Pd(OAc)_2_ as the catalyst precursor in toluene for 20 min. Various reaction parameters for the allylation reaction such as catalyst loading, time, temperature, solvent, and molar ratio of reagents were investigated and optimized. Highest yields were obtained when the allylating agent (1-phenyl-1-propyne), the pronucleophile (amine, alcohol, carboxylic acid), benzoic acid, and Pd(OAc)/PS-TPP were used in a 1 : 1.2 : 10 mol% : 10 mol% and refluxed for 6–18 h in toluene. As shown in Table 51, the allylation protocol was successful with various *N*- and *O*-pronucleophiles. Interestingly, carboxylic acids (entries 6–8) were more effective as substrates for the allylation reaction than alcohols (entries 4 and 5). Weaker *N*-pronucleophiles such as those containing electron deficient anilines (entries 1 and 2) or are sterically hindered (entry 3) were well tolerated giving the desired allyl amines in good to high yields.

**Table 51:** Allylation of carboxylic acids, alcohols, and amines^a^

Entry	Pronucleophiles	Allylated products	Yield (%)
1	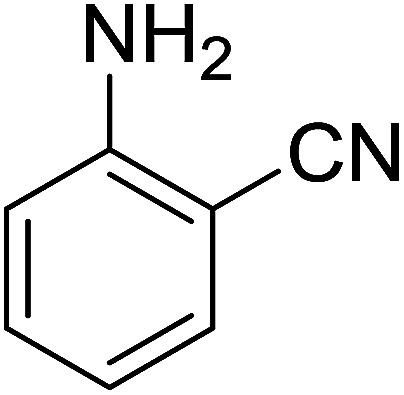	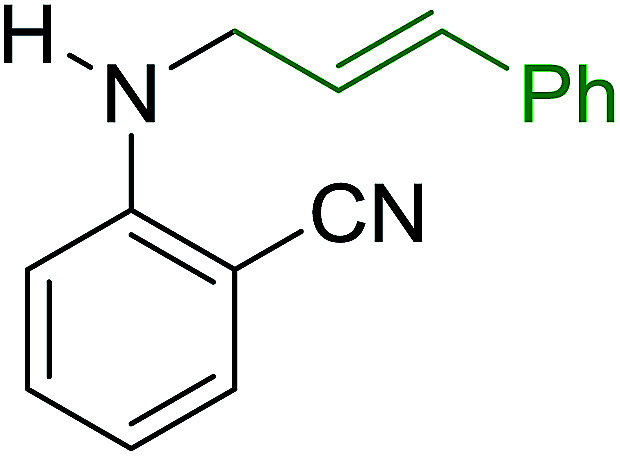	82
2	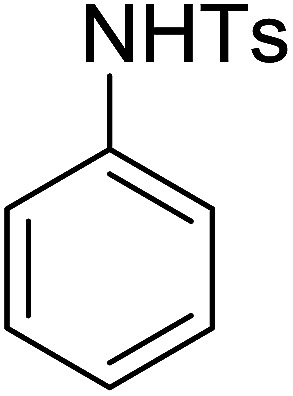	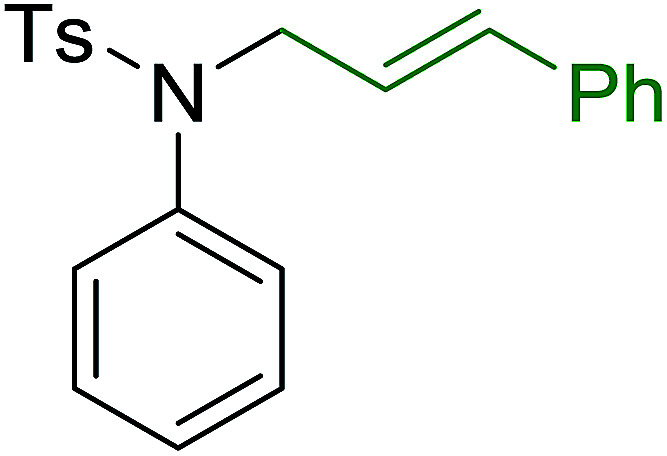	85
3	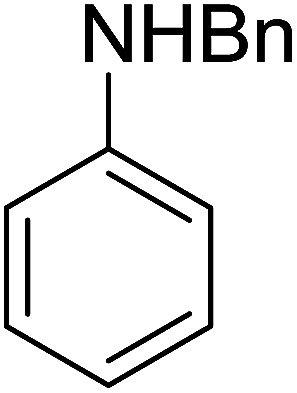	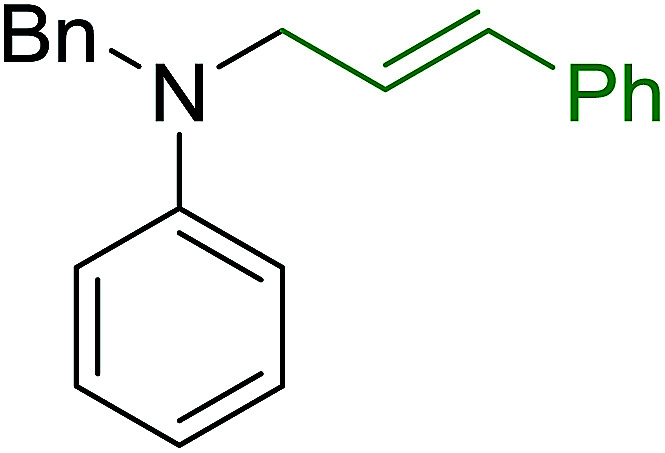	84
4	BnOH	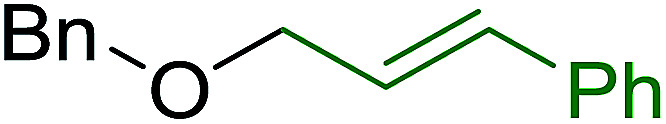	82
5	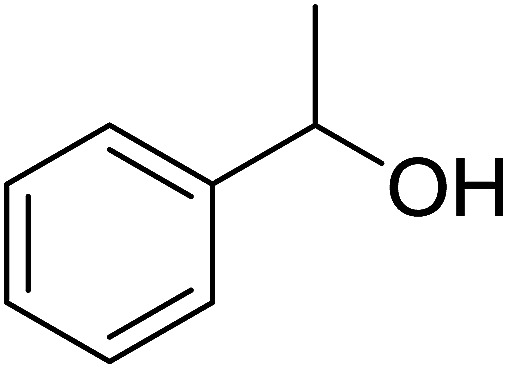	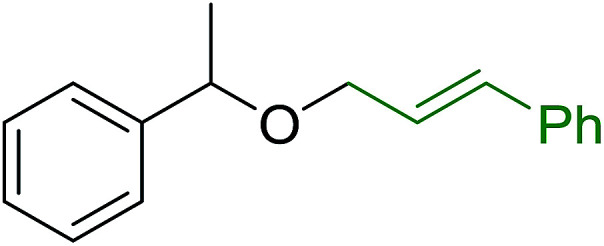	80
6	MeCOOH	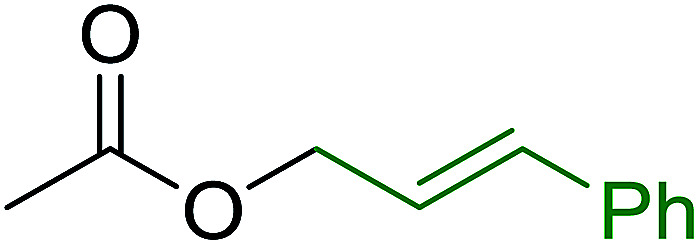	85
7	PhCOOH	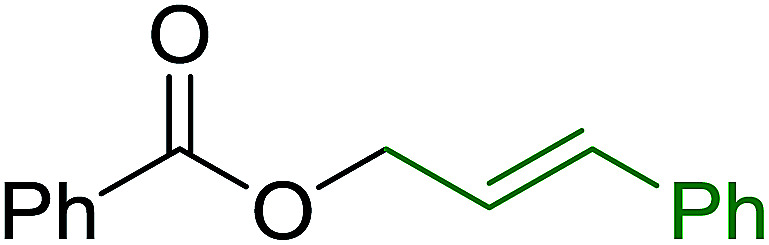	92
8	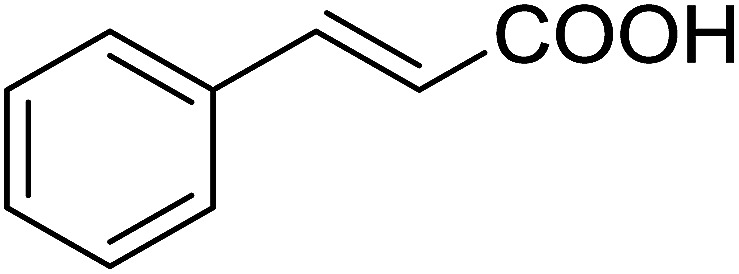	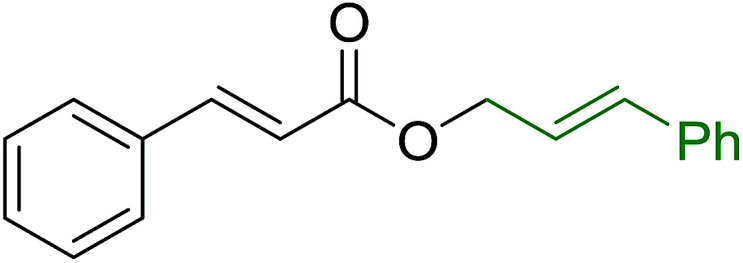	95

a Reagents and conditions: 1 : 1.2 : 0.1 : 0.1 molar ratios of 1-phenyl-1-propyne : pronucleophile (amine, alcohol, carboxylic acid) : benzoic acid : PS-TPP (5 equiv. to Pd(OAc)), toluene, 110 °C, 6–18 h.

## 8. Conclusion

Solid-phase synthesis continues to evolve as it offers important features such as removal of the product by filtration of the solid resin, easy handling, reduced side reactions, and recyclability of the solid matrix for repeated use. Although Ph_3_P is ranked as one of the worst atom-economic reagents known, polymer-supported triphenylphosphine has found many applications. This is because the commonly encountered problems in solution-phase chemistry involving Ph_3_P such as removal of excess Ph_3_P, Ph_3_P-complexes, and the by-product Ph_3_PO can be easily avoided with PS-TPP. Moreover, the byproduct PS-TPPO can be reduced to PS-TPP by treatment with trichlorosilane. In the past few decades since its first preparation in 1971, PS-TPP (1) has demonstrated wide applicability and its many proven applications have been well documented. Surprisingly, no comprehensive review has been dedicated to the versatile reagent as it has been often briefly mentioned in reviews of wider scope. The reagent has since been successfully used in the Mitsunobu reactions, the Staudinger reaction, and for the preparation of PS-TPP–halophosphorane complexes, Wittig reagents, and as a ligand for palladium, cobalt, ruthenium, gold, and rhodium complexes. The heavy metal catalysts proved more air stable and were utilized in transfer hydrogenation and hydrocarbon oxidation reactions, Pauson–Khand-, Nicholas-, and Heck reactions. Others have also used it for the mono-olefination of symmetrical dialdehydes, preparation of vinyl ethers, thioethers, isocyanates, dipeptides, acid chlorides, alkyl halides, amides, amines, dibromoalkenes, halohydrins, and esters. Furthermore, formylation of primary and secondary alcohols, as well as acetalization of carbonyl compounds has been demonstrated. The reagent has also been useful in the synthesis of heterocycles such as 2-phenylbenzothiazoles, 3-aminoindole-2-carbonitriles, 1,3,4-thiadiazole-2,5-dicarbonitrile, thiazole-2,4,5-tricarbonitrile, 1,2,4-oxadiazoles, vinylthio-, vinylsulfinyl-, vinylsulfonyl- and vinylketo-benzofuroxans and benzofurazans, and β-lactams. The reagent also has proven valuable for the reduction of steroidal ozonides, isomerization of *E*/*Z* mixtures of nitro olefins, and has been used occasionally as a linker for solid phase synthesis. Finally, the reagent was elegantly applied in the total synthesis of a small library of palmarumycin CP_1_ analogs and the total synthesis of epothilone A. PS-TPP demonstrated high success in a wide range of reaction types and rendered the purification process much more facile because the liberated phosphine oxide byproduct remains attached to the resin. Based on its past performance, it is likely that the reagent will make its debut in yet to be seen novel transformations.

## Conflicts of interest

There are no conflict of interest to declare.

## Supplementary Material
